# Internal migration and health in South Africa: determinants of healthcare utilisation in a young adult cohort

**DOI:** 10.1186/s12889-021-10590-6

**Published:** 2021-03-20

**Authors:** Carren Ginsburg, Mark A. Collinson, F. Xavier Gómez-Olivé, Mark Gross, Sadson Harawa, Mark N. Lurie, Keith Mukondwa, Chantel F. Pheiffer, Stephen Tollman, Rebecca Wang, Michael J. White

**Affiliations:** 1 Medical Research Council/Wits Rural Public Health and Health Transitions Research Unit (Agincourt), School of Public Health, Faculty of Health Sciences, University of the Witwatersrand, 27 St Andrews Road, Parktown, Johannesburg, 2193 South Africa; 2 Department of Science and Innovation/ Medical Research Council, South African Population Research Infrastructure Network, Johannesburg, South Africa; 3 Population Studies and Training Centre, Brown University, Providence, RI, USA; 4 Department of Epidemiology, Brown University, School of Public Health, Providence, RI, USA; 5 INDEPTH Network, Accra, Ghana

**Keywords:** Internal migration, Migrants, Healthcare utilisation, South Africa, Health and demographic surveillance system

## Abstract

**Background:**

In South Africa, human geographic mobility is high as people engage in both permanent and temporary relocation, predominantly from rural to urban areas. Such mobility can compromise healthcare access and utilisation. The objective of this paper is to explore healthcare utilisation and its determinants in a cohort of internal migrants and permanent residents (non-migrants) originating from the Agincourt sub-district in South Africa’s rural northeast.

**Methods:**

A 5-year cohort study of 3800 individuals aged 18 to 40 commenced in 2017. Baseline data have been collected from 1764 Agincourt residents and 1334 temporary, mostly urban-based, migrants, and are analysed using bivariate analyses, logistic and multinomial regression models, and propensity score matching analysis.

**Results:**

Health service utilisation differs sharply by migrant status and sex. Among those with a chronic condition, migrants had 0.33 times the odds of non-migrants to have consulted a health service in the preceding year, and males had 0.32 times the odds of females of having used health services. Of those who utilised services, migration status was further associated with the type of healthcare utilised, with 97% of non-migrant rural residents having accessed government facilities, while large proportions of migrants (31%) utilised private health services or consulted traditional healers (25%) in migrant destinations. The multinomial logistic regression analysis indicated that, in the presence of controls, migrants had 8.12 the relative risk of non-migrants for utilising private healthcare (versus the government-services-only reference category), and 2.40 the relative risk of non-migrants for using a combination of public and private sector facilities. These findings of differential utilisation hold under statistical adjustment for relevant controls and for underlying propensity to migrate.

**Conclusions:**

Migrants and non-migrants in the study population in South Africa were found to utilise health services differently, both in overall use and in the type of healthcare consulted. The study helps improve upon the limited stock of knowledge on how migrants interface with healthcare systems in low and middle-income country settings. Findings can assist in guiding policies and programmes to be directed more effectively to the populations most in need, and to drive locally adapted approaches to universal health coverage.

**Supplementary Information:**

The online version contains supplementary material available at 10.1186/s12889-021-10590-6.

## Background

With increasing levels of international and internal migration in low- and middle-income countries, the health implications of mobility are a growing focus of attention. As countries seek to fulfil the targets set out in the United Nations Sustainable Development Goals, the need to consider geographically mobile populations in planning and policy has been emphasised [1]. In recognition of the sparse evidence on the migration and health relationship, development agencies, policy makers and the research community have been called upon to urgently address these knowledge gaps [2]. Understanding the dynamics of migration and health both regionally and globally is a public health priority. It is imperative in moving towards universal health coverage that mobile individuals are incorporated into policy and planning [3].

Internal migration, the movement of people within a country’s borders, and urbanisation have been proceeding more rapidly in Africa than many other regions. Africa’s urban population is expected to increase from 43 to 59% by the year 2050 [4], while the intensities and types of movements occurring within African countries are diverse and multifaceted [5, 6]. Comparable data on levels of internal migration in Africa are limited, with South Africa and Zambia displaying higher levels of internal migration among countries in the Southern African region [6]. Within South Africa, geographic mobility is prevalent as people engage in both permanent relocation, as well as circular and temporary movement. Circular migration was historically connected with the Apartheid system of movement control, where black South Africans, who were recruited to work in mines and urban centres, were restricted from permanently settling in these areas [7]. This resulted in members of the work force, typically male, oscillating between urban work places and rural permanent homes [8]. Indeed South and Southern Africa’s economic foundation was built on both internal and cross-border labour migration, with migrant remittances providing significant support to origin households and communities [9]. These interconnections between urban and rural areas have persisted post-Apartheid. In contemporary South Africa, the prevalence of internal migration, which is largely labour related, far exceeds that of cross-border movement, with the most recent population census indicating 5% of the population had moved within the country in the 5 years preceding the census, compared with 1% of the population having immigrated from outside of the country’s borders [10]. Internal migration in South Africa may take on multiple forms and is undertaken by a diverse range of individuals. Recent analysis of South Africa’s 2011 population census highlights age, gender and education as key individual-level predictors and correlates of internal migration [11]. Employment and job seeking are often drivers of such movement, with municipalities that have higher unemployment levels experiencing relatively higher levels of out-migration [11]. Internal migration is most commonly undertaken by young adults and internal migration streams, while still predominantly male, are becoming increasingly feminised [11, 12]. While the largest proportion of movements occurring internally in South Africa involve a net distribution toward more urban settlement types, there remain strong, continuing inter-connections between rural and urban areas of the country [10]. Interregional migration and urbanisation are typically associated with individual and societal socioeconomic improvement; yet, any migration poses a challenge to the planning of health and social systems, which is often premised on a stable catchment population. The temporary and circulatory nature of contemporary internal movements exacerbates this challenge. It is therefore important to gain a better understanding of the levels and trends of internal mobility in the country, as well as the impacts of such mobility on productivity, health and wellbeing.

The relationship between migration and health is complex and presents methodological challenges. The health status of migrants may differ from that of non-migrants prior to migrating, at the time of migration and thereafter, making it difficult to disentangle selection effects and the direct effects of migration [13, 14]. Indeed many comparisons of migrants and non-migrants (or urban and rural residents), while identifying differentials at a point in time, are insufficiently attentive to these selection mechanisms. Furthermore, the event of migration itself may produce health changes at key stages of the life course, often attributed to the stress of relocation or the action of repeated movements. Following relocation, migrants are often exposed to a different social, environmental and health regime [15]. These have been described as disruption effects that occur around the time of migration [16–18]. It follows that mobility, which results in an altered set of circumstances, may compromise healthcare access and continuity of care for individuals requiring treatment for chronic conditions.

South Africa is a key setting in which to investigate these issues, not only because of the widespread manifestation of migration within the country, but also because the migration-health relationship in South Africa is likely to be a harbinger for other societies in transition in sub-Saharan Africa. Coupled with high levels of internal mobility, South Africa is experiencing an ongoing infectious disease (ID) burden with an estimated 19% of the adult population HIV positive [19]. At the same time a growing burden of non-communicable diseases (NCD) has been observed [20, 21]. South Africa’s internal migrants have a significantly higher burden of HIV compared with non-migrants and are at higher risk of HIV acquisition [22, 23]. A longitudinal study of premature mortality among internal migrants from the African Health Research Institute and the Agincourt Health and Demographic Surveillance Systems (HDSS) revealed that return migrants to these HDSS areas had a four times higher risk of mortality from AIDS/TB and NCD as compared with permanent residents, suggesting a marked mortality disadvantage among migrants [24]. Whether suffering from a non-communicable or infectious disease (or increasingly both), individuals with chronic conditions require ongoing treatment and regular medical follow-up [25]. However, many remain undiagnosed, commence treatment later than recommended, or are unable to adhere to long-term treatment, resulting in poor health outcomes [26–28]. Migration, particularly migration of a temporary nature as is prevalent in South Africa, can compromise adherence to and continuity of healthcare. However, not enough is known about issues concerning healthcare access and utilisation among migrants in the country. Such information is vital to South Africa’s overall policy goal of achieving universal health coverage [29].

Barriers to healthcare utilisation have been investigated to a limited extent among non-migrant populations in South Africa, with issues regarding the perceived quality of public healthcare, costs associated with private healthcare, and migration status being highlighted as challenges [30–32]. A few small specialised surveys or qualitative studies of cross-border migrants have identified barriers and difficulties in accessing services, and these studies are strongly suggestive of the issues that arise for internal migrants as well. Deficient access to information, language barriers, and negative interactions with healthcare providers all may discourage health seeking [33–37]. Consequently, migrant populations appear less likely to engage with the healthcare system [38]. South Africa’s present health system does not adequately address such challenges of access amongst mobile populations [39, 40]. While emphasis is often placed on cross-border migrants, the fact that internal migrants are large in number, and are themselves often moving substantial distances to destinations that may be socioeconomically and linguistically very different from their origin, argues further for attention to this group.

To respond to the urgent need for a strengthened knowledge base, this paper examines self-reported health and healthcare utilisation among internal migrants and rural-based permanent residents (i.e. non-migrants) originating from the Agincourt HDSS in South Africa’s rural northeast. The paper examines the profile of migrants compared with non-migrants to provide insight into the demographic, socioeconomic and health dimensions upon which migrants are selected. It further aims to identify the determinants of healthcare utilisation by migration status in the presence of appropriate statistical controls, and adjusting for the underlying propensity to migrate. We hypothesise that migrants are less likely to access health services compared to non-migrants, and that factors such as sex (gender), employment status and migration geography may contribute to differential health service use.

## Methods

### Study population

**Fig. 1:**
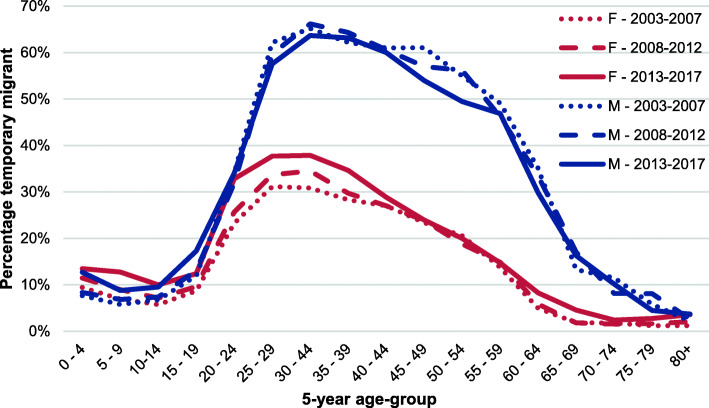
Density of temporary migration (shown as percentage temporary migrant) in the Agincourt study population, in three successive five-year historical periods: 2003–2007, 2008–2012, 2013–2017

The study uses data from the Agincourt HDSS, which is located in the Bushbuckridge district, Mpumalanga province, situated about 500 km north east from Johannesburg, South Africa’s main metropolis. The Agincourt HDSS was established in 1992 and has monitored all births, deaths and in- and out-migrations taking place within the 400 km^2^ (km) study site since inception. The surveillance population currently comprises 116,000 people living in 31 villages [41]. Included in the population under surveillance are temporary migrants, defined as household members who are away from home for more than 6 months in the previous year, but retain significant links to their origin households [10]. The HDSS method documents individuals moving out of the HDSS origin areas, and captures their return to the HDSS origin area where applicable. The majority of Agincourt temporary migrants relocate to urban areas of the Gauteng province (location of Johannesburg) where they are more likely to find employment opportunities [10, 42], while others move shorter distances to areas 50 to 150 km away from the Agincourt HDSS. Figure 1 shows the age-sex profile of temporary migration in the surveillance population. The age-group that has the highest likelihood of temporary migration is 18 to 40 years, for both sexes. Three things stand out from the figure. There is a high level of male temporary migration, with over 60% of men aged between 30 and 44 years participating in a temporary migration lifestyle. This implies, by our definition, that they spend a majority of time away from home and residing at the migration destination. Secondly, while male migration likelihood is high, the level shows a declining trend over time for adults. Thirdly, the trend of temporary migration for young adult women is rapidly increasing over time. The percentage of female temporary migrants in the age group 25 to 44 years was 31% in 2003, and rose to 38% in 2017. This implies that young women are increasingly migrating from rural populations to work and school opportunities in urban areas.

### Fieldwork methods

Following a successful pilot study undertaken in 2012 to inform on the value and feasibility of following migrants using a surveillance platform [43], a 5-year cohort study of 3800 individuals aged 18 to 40 commenced in 2017. The Migrant Health Follow-Up Study (MHFUS) aims to better understand relationships between migration, urbanisation, and health in a transition setting through following-up migrants who leave the Agincourt study area, usually to access employment. The cohort was randomly selected using a simple random sample of 18 to 40 year-olds from the Agincourt HDSS longitudinal research platform, and it successfully enrolled 3098 individuals into wave 1 of the longitudinal survey. The age group 18–40 was chosen to emphasise younger individuals, and to capture the age range and associated health and social transitions in ages where temporary migration is most prevalent. At the time of enrolment, the cohort included people at different stages of practise regarding migration, with some cohort members living away from their areas of origin (while maintaining contact with their origin households) having already undertaken a temporary migration, and others permanently resident in the Agincourt sub-district. The study is designed to follow all individuals regardless of subsequent migration (residence) status, therefore capturing any new and return migrants over time, and retaining permanent out-movers in the sample. Similarly, changes in residence among migrants in destination areas will also be documented. The study will constitute 5 waves of cohort observation that will be facilitated through a combination of face-to-face interview (in waves 1 and 4 of the study), and telephone interviews (in waves 2, 3 and 5 of the study). This paper reports baseline results from wave 1 of the study comprising face-to-face interviews that were conducted in 2018.

In this first phase of the study, a fieldwork team visited the HDSS-located origin household of each randomly-selected respondent to collect information on the current location of the respondent, and their telephone contact details. Respondents either resided in the HDSS study area, or had migrated elsewhere. In the case of non-HDSS residents (temporary migrants), the team were able to collect information on their whereabouts by making first contact with their family households. Information about the respondent’s location was used to split fieldwork operations into two fieldwork teams. One team focused on conducting face-to-face interviews with study participants in and around the Agincourt sub-district, and the other team conducted interviews with migrant respondents located in the Gauteng province and other areas more distant from the Agincourt study site. Using mobile phones to make appointments with study participants, a mobile fieldwork team administered interviews at respondents’ migrant’s places of work or residence. Face-to-face interviews were successfully conducted with 2464 members of the cohort (79.5%), and 634 interviews were conducted over the telephone (20.5%).

Our sample represents 82% of 3800 individuals originally drawn from the HDSS database. Of the 702 individuals who were not interviewed, 52% either refused to participate or missed a number of appointment attempts; 22% were untraceable (the origin households had moved or dissolved); 15% had migrated permanently in earlier years of the study and were not contactable through an origin household; 6% were ineligible for participation in the study due to disability or other incapacitation; 1% were determined to be outside of the eligible study population age range, and 4% had died.

Interviews were conducted with a standardised tablet-based questionnaire collected and managed using REDCap electronic data capture tools hosted at the University of the Witwatersrand [44, 45]. The questionnaire, developed for the MHFUS, included mostly closed-ended questions on social and economic life, health and wellbeing, as well as traditional demographic indicators. Productivity and livelihoods were examined with questions on educational status, employment and remittance sending. Individual health status was ascertained through questions about respondents’ perceptions of their general health in the year prior to the survey, any prior diagnosis of a chronic condition, and details about utilisation of health services. The questionnaire probed healthcare-seeking behaviours by asking respondents whether they had used any health services in the past year, and requesting information on the type of services utilised, and whether public or private sector. Migrants were asked about services used in migrant destinations, while non-migrants responded on the use of services in or around their places of origin. The full questionnaire for wave 1 of the MHFUS is available in Supplementary File 1.

### Statistical analysis

Descriptive statistics are used in bivariate analyses to compare migrants with permanent residents (non-migrants) of the HDSS. Migrant status is analysed as a dichotomous variable to contrast individuals living in the study area who had not migrated with those who were living outside of the study area at the time of interview. Differences between migrants and non-migrants are tested using χ^2^ and t-tests where applicable. Expanding to multivariate analyses, a logistic regression model predicting migrant status is estimated to examine the profile of migrants and non-migrants according to demographic, socioeconomic, and health characteristics. This sheds light on the characteristics that are selective for migration status in this population.

In the first step of the regression analysis of healthcare utilisation, binary logistic regression models are estimated to investigate predictors of service use, conditional on having received a prior diagnosis. In the second step, we conduct a multinomial logistic regression analysis to investigate the association between migration status, the demographic and socioeconomic predictor variables, and the *type* of service used in year prior to the survey. In this analysis of the polytomous outcome, we distinguish among three categories: “government only”, “private only”, “both government and private”. Categories are mutually exclusive, and the multinomial logit model allows us to estimate the relative risk of one particular outcome compared to a base condition (reference), while also simultaneously estimating the relative risk of other possible outcomes. For both health service use itself and then for type of service used, we augment the conventional regression approach with a propensity score matching (PSM) approach to assess the impact of migrant status. The propensity score approach allows us to conceptualise migration as a treatment (within a causal modelling framework) and estimate the average treatment effect of migration among statistically adjusted equivalent groups. In applying these matching techniques, we confirmed the presence of satisfactory overlap in the propensity distribution across groups and made use of a single match per observation. Alternative models that adjusted for non-response based on our original surveillance system sample draw gave nearly identical results to those presented here. Substantive interpretations would not differ. All statistical analyses were performed using STATA version 14.2 [46].

## Results

### Descriptive results

The socio-demographic and health characteristics of the study participants are presented in Table 1, with HDSS residents (non-migrants) contrasted with migrants. The mean age of participants was 28.3, with migrants being slightly older than non-migrants (29.0 and 27.8 years respectively, *p* < 0.001). Migrants were significantly more likely to be male (57.9%), while 55.5% of non-migrants were female (*p* < 0.001). On average, migrants were more highly educated than non-migrants: the majority of migrants (75.6%) had completed high school or attainted a post-school qualification, compared with 50.9% on non-migrants (*p* < 0.001). Migrants were also more likely to be employed at the time of the interview (66.3%), while the largest proportion of non-migrants were unemployed and looking for work (46.1%, *p* < 0.001). The majority of both migrants (65.3%) and non-migrants (59.9%) who were employed indicated that they had permanent positions, while 16.0% of non-migrants indicated that their work was irregular as compared with 7.1% of migrants (*p* < 0.001, details not shown). The type of employment largely differed for migrants and residents, and by sex. In an examination of occupation for those employed (details not shown) we find that non-migrant females were most commonly doing domestic or cleaning work (22.3%), while the highest proportion of migrant females were employed in retail/sales work (18.5%). Non-migrant males were commonly employed in the construction industry or as drivers (29.6%), while 14.7% were employed as skilled workers (plumbers, mechanics or electricians). Similarly, 14.9% of migrant males were employed as skilled workers, while 11.5% were employed in the mines, and 8.9% as drivers. These employment patterns are a close match to the patterns reported for the whole surveillance population, amidst a rising aspiration for employment, in both sexes, despite high and rising levels of unemployment [12].

**Table 1:** Profile of migrants and non-migrants

	Full Cohort		Non-Migrant		Migrant		***p***-value
	(***n*** = 3098)		(***n*** = 1764)		(***n*** = 1334)		
	n	%	n	%	n	%	
Age							
Mean (SD)	28.3 (5.8)		27.8 (6.0)		29.0 (5.3)		*p* < 0.001
Min, Max	18, 41		18, 41		18, 41		
Sex							
Male	1558	50.3	785	44.5	773	57.9	*p* < 0.001
Female	1540	49.7	979	55.5	561	42.1	
Education Status							
Primary school or lower	135	4.4	106	6.0	29	2.2	*p* < 0.001
High school incomplete	1056	34.1	760	43.1	296	22.2	
Matric or post school	1906	61.5	898	50.9	1008	75.6	
Missing	1	0.0	0	0.0	1	0. 1	
Employment status							
Not in labour force	586	18.9	408	23.1	178	13.3	*p* < 0.001
Unemployed	1085	35.0	813	46.1	272	20.4	
Employed	1427	46.1	543	30.8	884	66.3	
Self-reported health							
Poor/ average	145	4.7	70	4.0	75	5.6	*p* < 0.05
Good	2951	95.3	1692	95.9	1259	94.4	
Missing	2	0.1	2	0.1	0	0.0	
Self-reported HIV							
Positive	234	7.5	185	10.5	49	3.7	*p* < 0.001
Negative	2864	92.5	1579	89.5	1285	96.3	
Ever Diagnosed with a chronic illness							
Yes	336	10.8	261	14.8	75	5.6	*p* < 0.001
No	2759	89.1	1500	85.0	1259	94.4	
Missing	3	0.1	3	0.2	0	0.0	
Chronic Medication (If ever diagnosed)							
Yes	292	86.9	230	88.1	62	82.7	NS
No	43	12.8	30	11.5	13	17.3	
Missing	1	0.3	1	0.4	0	0.0	
Used health services in the past year							
Yes	1607	51.9	991	56.2	616	46.2	*p* < 0.001
No	1488	48.0	771	43.7	717	53.7	
Missing	3	0.1	2	0.1	1	0.1	

**Fig. 2:**
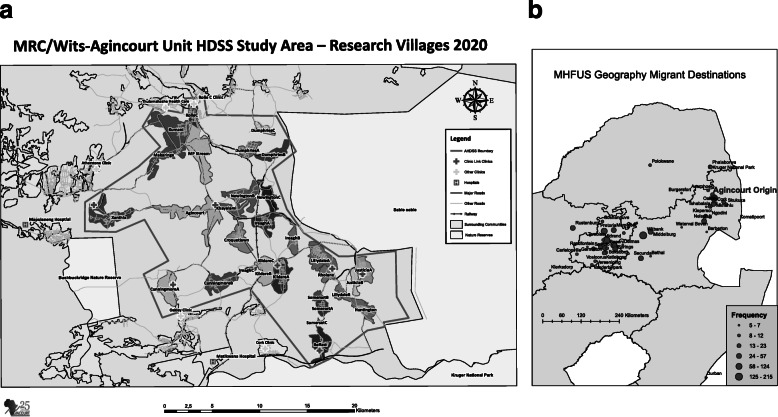
**a** The Agincourt HDSS is located in the sub-district of Bushbuckridge, Mpumalanga province South Africa and is situated about 500 km north-east of Johannesburg (Map generated by ArcGIS version 10.4.1), **b** The map, generated using ArcGIS Desktop 10.8.1, indicates the distribution of migrant destinations

The geographical distribution of migrants’ destinations from the Agincourt study site is presented in Fig. 2. Distance assignments were made on the basis of kilometres between the Agincourt origin area (from the Agincourt field office) (Fig. 2a) and the migrant’s current place of residence using existing roads (Fig. 2b). The largest proportion of migrants (*n* = 735, 55.1%) had moved distances in excess of 400 km from their origin households, and most were located within the Gauteng province. A substantial proportion of migrants had relocated to distances of 150 to 400 km away from their origin villages (*n* = 342, 25.6%), while 19.3% (*n* = 257) had moved to mostly rural areas within 150 km from the study site.

Participants were asked about their perceptions of their general health. A higher proportion of migrants (5.6%) reported that they felt their health was average to poor (rather than good or very good) as compared with non-migrants (4.0%, *p* < 0.05). In relation to self-reported HIV status, a larger proportion of non-migrants reported a positive diagnosis (10.5%) compared to 3.7% of migrants (*p* < 0.001). The cohort were asked about manifest chronic conditions, with 14.8% of non-migrants and 5.6% of migrants indicating having received a diagnosis (conditions include hypertension, diabetes, HIV, TB) (*p* < 0.001). Of those who were diagnosed, 82.7% of migrants and 88.1% of non-migrants indicated that they were taking chronic medication (*p* = NS). In the year prior to the survey, fewer migrants (46.2%) compared with non-migrants (56.2%) indicated that they had used any health services (*p* < 0.001).

**Fig. 3:**
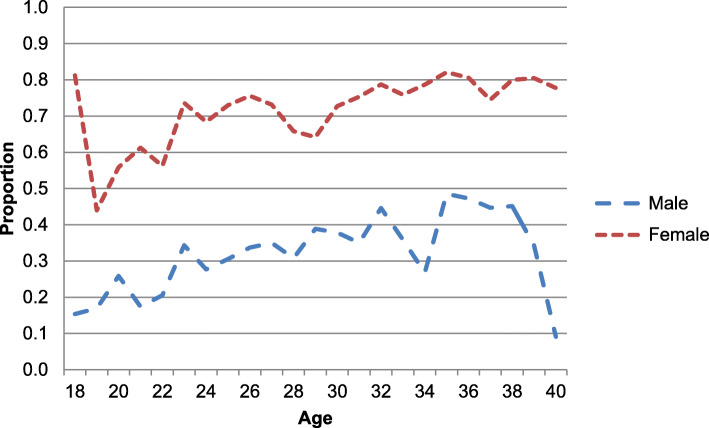
Healthcare utilisation by age and sex, *n* = 3095

**Fig. 4:**
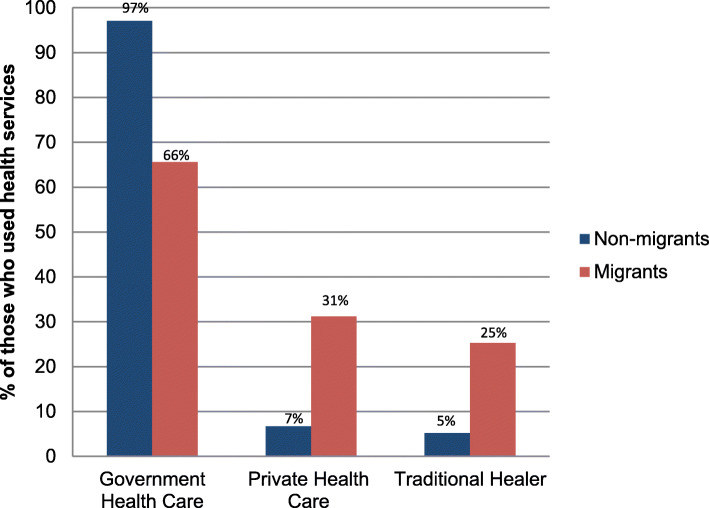
Healthcare utilisation by migrant status (for those who used services in the past year), *n* = 1607

Patterns of health service utilisation were found to differ significantly by sex (Fig. 3). In the year before the survey, females of all ages (71.0%) were more likely than males (33.1%) to have utilised healthcare services (*p* < 0.001). The majority of non-migrants who had utilised health services in the year prior to their interview visited government facilities (97.1%), while only 65.6% of migrants who had used services, had used of government facilities (Fig. 4) (*p* < 0.001). In addition, migrants were much more likely than non-migrants to have visited a private health clinic (31.2% of migrants who had used services compared to 6.7% of non-migrants, *p* < 0.001) or traditional/spiritual healer (25.3% of migrants compared to 5.2% of non-migrants, *p* < 0.001).

### Multivariate models

**Fig. 5:**
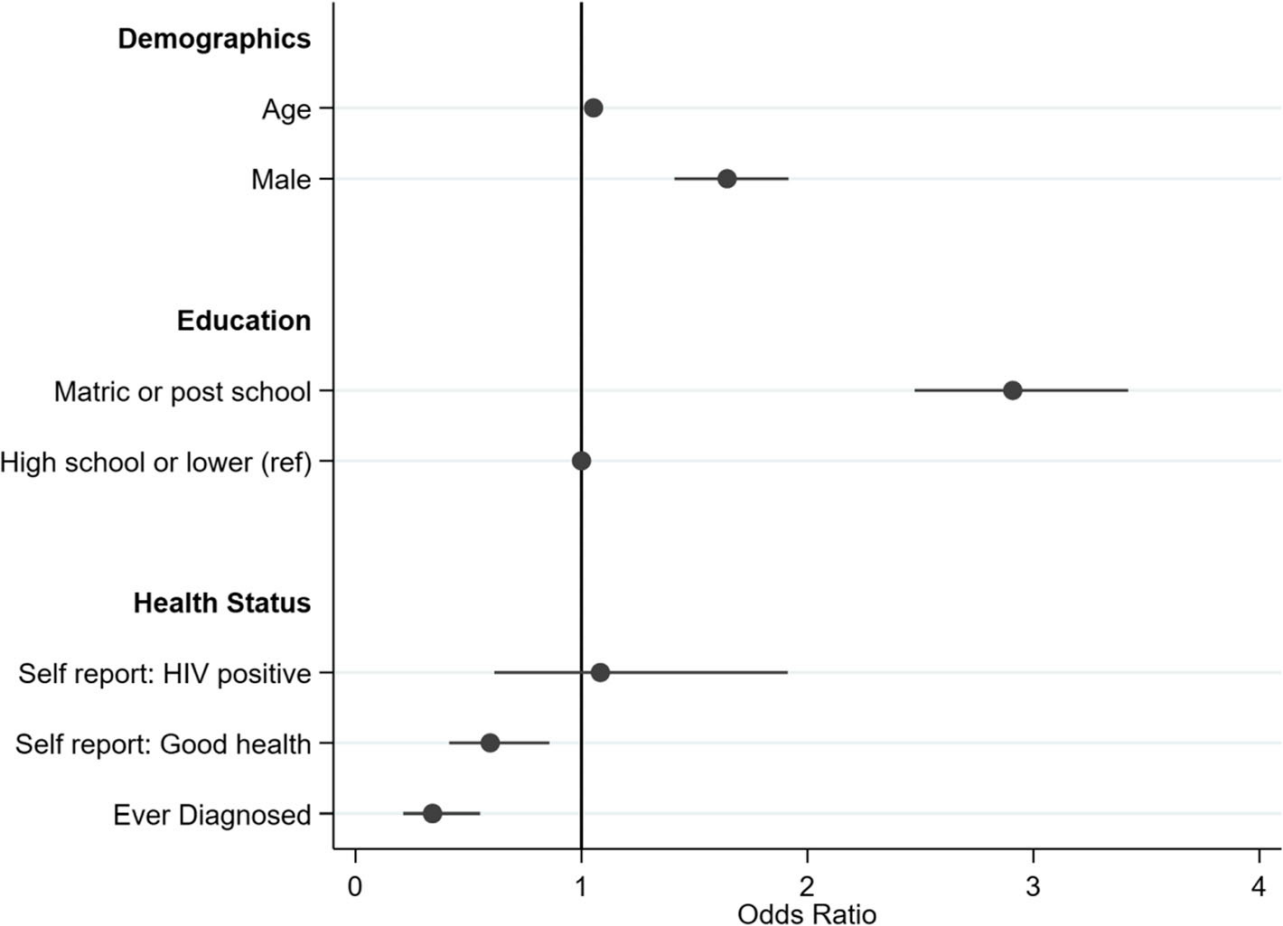
Logistic regression: profile of migrants ((*n* = 3094, LR χ_(6)_^2^ = 356.63, *p* = 0.000)

**Fig. 6:**
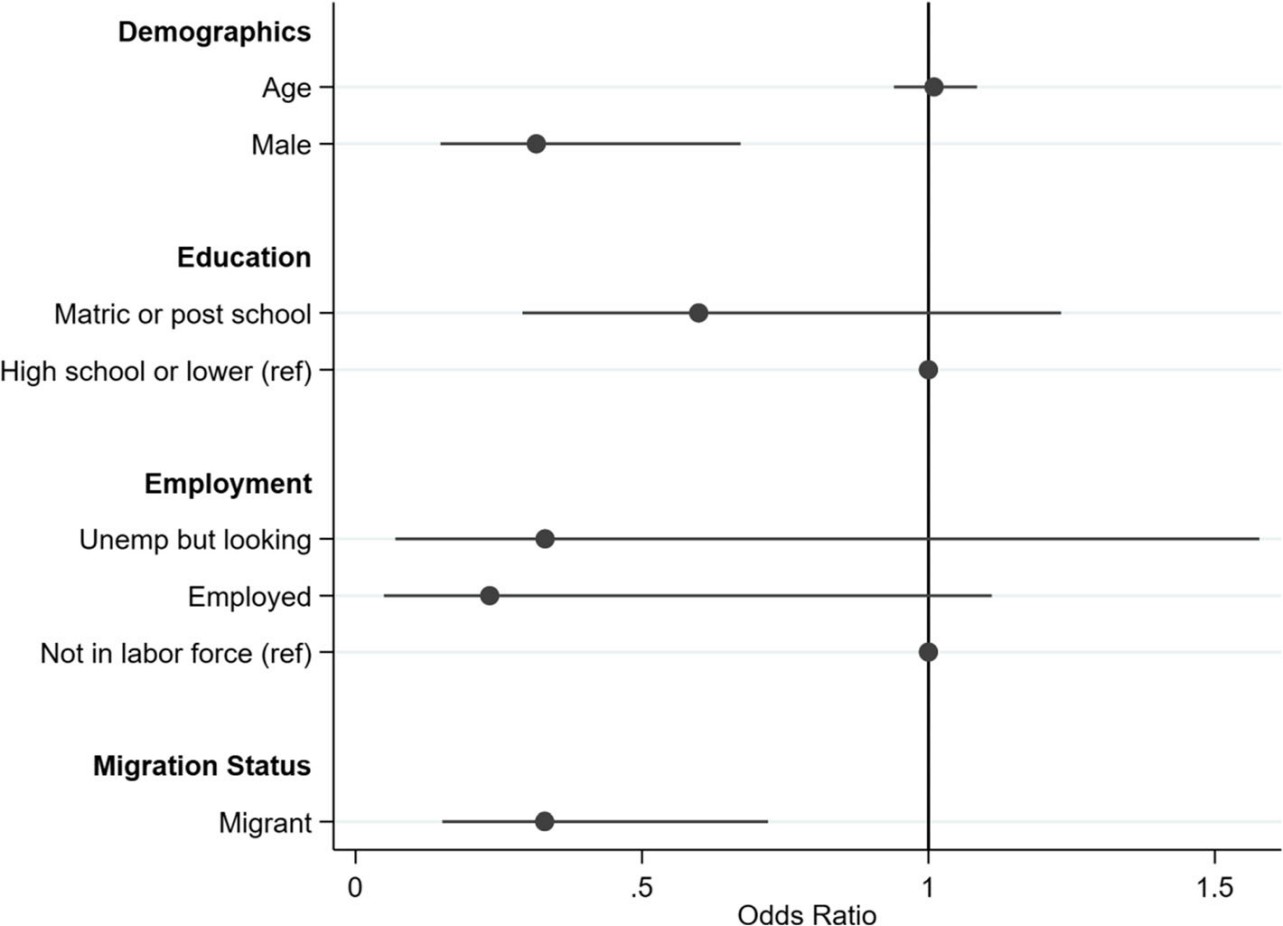
Logistic regression: health service use by migrant status among those with a prior diagnosis of a chronic condition (*n* = 335, LR χ_(6)_^2^ = 29.49, *p* = 0.000)

Figures 5 and 6 report results of multiple variable regression analyses predicting migration status and healthcare service use, respectively. The binary logistic regression on migrant status sheds initial light on the selectivity of migration, and it provides indicators of variation by demographic, education and health status characteristics. The coefficient plot is presented in Fig. 5.^1^ The odds of males migrating were 1.64 times that of females (CI: 1.4 1.9, *p* < 0.001), while the odds of migration among those with completed high school or a post school qualification were 2.91 times those of cohort members with incomplete high school, or lower levels of education (CI: 2.5 3.4, *p* < 0.001). Migrants had 0.34 times the odds of non-migrants of having been diagnosed with a chronic condition (CI: 0.2 0.6, *p* < 0.001), and were also 0.60 times as likely as non-migrants to have reported good or very good general health (CI: 0.4 0.9, *p* < 0.01).

Figure 6 presents results of a logistic regression analysis of healthcare service use among those diagnosed with a chronic condition. Among those with chronic conditions, males had 0.32 times the odds of females of having used health services (CI: 0.2 0.7, *P* < 0.01), while migrants had 0.33 times the odds of non-migrants to have consulted a health service (CI: 0.2 0.7, *P* < 0.001). These sharply differing outcomes for migrants and non-migrants are reinforced by propensity score matching analysis. Our propensity score model returns an average treatment effect (ATE) of − 0.15 (*p* = 0.031) on the logit outcome (equivalent to an odds ratio of 0.86), pointing to lower health care service use among migrants. The PSM analysis indicates that a portion of the health utilisation differential between migrants and non-migrants is due to their heterogeneity in composition, but even after all efforts to remove such selection effects, a significant difference persists in their utilisation of care.

The results of the multinomial logistic regression analysis of the *type* of service used conditional on having used a healthcare service in the previous year are presented in Table 2. The type of facility used by migrants differed significantly from non-migrants across the three outcomes analysed simultaneously: (1) government facilities only, (2) private facilities only and (3) both government and private facilities. Migrant behaviour is revealed to be substantially different from non-migrants, even in the presence of demographic controls. Using government services only as a reference category, migrants had 8.12 the relative risk of non-migrants for utilising private healthcare in the presence of controls (CI: 4.9 13.5, *P* < 0.001), and 2.40 the relative risk of non-migrants for using a combination of public and private sector facilities (CI: 1.5 3.7, *P* < 0.001). Males who utilised services were more likely than females to have visited private services exclusively, as compared to government facilities (relative risk ratio (RRR) =2.5; CI: 1.7 3.7, *P* < 0.001). Those with higher levels of education had 5.24 (CI: 3.0 9.1, *P* < 0.001) and 3.20 (CI: 1.9 5.3, *P* < 0.001) times the relative risk of those with incomplete high schooling for accessing either private facilities only or a mix of private and government facilities respectively, as compared with government services.

**Table 2:** Multinomial logistic regression model: type of healthcare services used for those who used services

Base: Government only	Private only			Both government and private		
	RRR	SE	95% CI	RRR	SE	95% CI
Age	1.05*	0.02	(1.01 1.10)	1.03	0.02	(0.99 1.08)
Sex						
Male	2.52***	0.51	(1.70 3.74)	1.25	0.28	(0.80 1.95)
Female (Ref)						
Education status						
Matric or post school	5.24***	1.47	(3.02 9.09)	3.20***	0.84	(1.91 5.33)
High school or lower (Ref)						
Employment status						
Unemployed	0.72	0.31	(0.31 1.65)	0.65	0.22	(0.34 1.27)
Employed	1.89	0.72	(0.90 4.00)	1.02	0.34	(0.53 1.97)
Not in labour force (Ref)						
Migrant status						
Migrant	8.11***	2.11	(4.88 13.50)	2.40***	0.54	(1.53 3.74)
Non-migrant (Ref)						
Constant	0.00***	0.00	(0.00 0.01)	0.01***	0.01	(0.00 0.04)

*n* = 1520, * *p* < 0.05; **p < 0.01; ***p < 0.001

LR χ_(12)_^2^ = 350.63; *p* = 0.000; Pseudo R^2^ = 0.20

To investigate the implications of a matching approach to this outcome, we again made use of propensity score models. We formulated a binary model as a choice between use of any private health care services (solely or mixed) versus use of government services only, for those individuals who used services, as before. Here the implicit causal effect of being a migrant was very strong in favouring private or mixed care, with an average treatment effect of 0.20 (*p* < 0.001) on the untransformed logit (equivalent to an odds ratio of 1.22). We also examined PSM models for the binary choices between private care only versus government only (ATE 0.18, *p* < 0.001) and mixed care versus government only (ATE 0.10, *p* = 0.001). These statistical results not only corroborate those of Table 2, but they also suggest that among otherwise equivalent persons, the experience of migration favours seeking health care through the private sector, even to the exclusion of the government sector.

## Discussion

Internal migration, largely a labour-related activity, incorporates a substantial proportion of South Africa’s working population who contribute directly to the economic base of the country, and the livelihoods of rural households and communities. Securing quality public healthcare for internal migrants will make a substantial contribution to their ongoing health and productivity and thus, indirectly, to the wellbeing of their origin communities and society. This is the broad motivation of the Migrant Health Follow-Up Study on which this paper is based. This analysis of baseline data from the MHFUS adds to the limited knowledge about the health of internal migrants, and offers important insights on how they interact with the healthcare system in South Africa. This is particularly pertinent to current South African discourse and engagements around the planning and implementation of National Health Insurance which aims to provide quality and accessible to health care to all [29, 47].

The study was designed to include both migrants and permanent residents of the rural sub-district population in order to examine the demographic, socioeconomic and health dimensions upon which migrants are selected. In keeping with general selectivity findings about migration in other parts of the world, migrants in this cohort of 18 to 40 year-olds are more likely to be male, and have relatively higher levels of education compared to non-migrants [48, 49]. At the same time, the large proportion of female migrants in the cohort (41% of migrants), resonates with the period trends observed in the Agincourt surveillance population as a whole, and elsewhere in South Africa, concerning the increasing feminisation of internal, labour migration [50].

With respect to selection on health-related characteristics, our multivariate results point to important variation in the conditions associated with who becomes a migrant. Individuals who received a diagnosis of a chronic condition and those who reported a positive HIV status are also more likely to be migrants. Such results suggest a positive selection (favouring healthier individuals) in the migration process. Nevertheless, migrants’ self-rated health was lower compared to non-migrant participants alluding to the possible disruptive effects of movement on perceived health, or the effects of increased expectations and a change in the reference category to a more advantaged (urban) population. Such health assessments reflect a combination of individual characteristics and expectations, prior health experiences and engagement with health services systems, and an individual’s reference group [51]. This observed difference in self-rated health highlights the important ways the migration process may interact with these factors. Our subsequent statistical modelling (for use of services and for source of care) recognises this selectivity and is designed to compensate for it and retrieve informative estimates in the manner of an experimental intervention.

Our results notably show that migrants and non-migrants utilised health services differently, both in overall use and in the type of healthcare consulted. These findings of differential utilisation hold under statistical adjustment for relevant controls and for underlying propensity. Non-migrants were significantly more likely to have accessed health services in the preceding year as compared with migrants. Among those in our sample with a diagnosis of a chronic condition, non-migrants were again more likely than migrants to have sought health services. This highlights possible barriers to access where migrants with chronic conditions may not follow up on their healthcare as readily as non-migrants. Reasons reported by those who failed to access treatment suggest that time constraints, being treated poorly on a previous visit, and congestion at health facilities in urban areas may translate into lower levels of service use. Additionally, health service utilisation was far more common in females (both migrant and non-migrant) compared with males. This is consistent with findings from other studies that have examined patterns of health service use by gender in South Africa specifically and Southern Africa generally [40, 52], as well as in other high-income country contexts [53].

A strong finding of the study is the difference between migrants and non-migrants in the type of health services they accessed. There are a number of possible explanations for why migrants appear to use more private health services as well as traditional healers. Private services may be more readily available in urban areas, and are sometimes provided by large companies; in addition, migrants - more likely to be employed - may have more resources to direct towards healthcare and choose a private rather than government provider. Both private healthcare facilities and traditional healers are more expensive than government healthcare facilities, suggesting that migrants may be able to pay more for the convenience and time-efficiency of private care [54]. Research conducted on the use of traditional healers in the Agincourt sub-district shows that traditional healers treat a wide range of illnesses which suggests that cultural familiarity provides a reason for migrants seeking treatment from traditional healers, potentially influenced by type of condition [55]. Another possible reason for more frequent use of private health services and traditional healers among migrants relates to challenges in accessing public health services at the destination place (these may include navigating the urban setting, transportation, and time constraints). Knowledge about public health services seems better in the place of origin, and going to a public health clinic is more likely done from home. Finally, the difference between migrant and non-migrants in the types of services accessed may be reflective of the limited healthcare options available to rural residents. These findings lay the ground work for qualitative investigations of health seeking behaviour and experiences of utilising services, which will be nested in future waves of the MHFUS.

Migrants accessing private healthcare at their migration destinations will likely need to traverse both public and private health systems and/or re-engage with rural public health systems on return home to rural origin areas, all of which increases the risk of disruption in care. Of further importance are those 48% of the study participants who make no use of health services, since they may be particularly susceptible to illness, including HIV. Poverty, geographical constraints, and high transportation costs, and in some cases, combinations of these barriers all contribute towards ultimate health service use or lack thereof [31, 47, 56, 57]. These results raise questions about the perceived quality of care, a possible lack of information on public health services or the ability of those who are employed, and better resourced, having wider set of healthcare options. Detailed information of the kinds of conditions for which migrants and non-migrants seek help when presenting to public versus private sector providers, as well as further detail on the distances travelled and reasons for not seeking care are questions that we aim to explore in subsequent rounds of the study, and through qualitative research methods.

Following-up mobile populations is challenging; and in the present study we acknowledge limitations relating to small losses to follow-up among individuals who may be particularly mobile and/or differ from the interviewed participants in relation to particular characteristics. As much as the survey instrument focused on capturing a broad range of aspects on migration, the high levels of mobility and circularity encountered in this study population challenged aspects of the research design, especially capturing detailed geography of repeated visits to different health providers. We further recognise that there are multiple approaches to classifying migrants (in relation to distance and length of residence in a destination) and these will be expanded in further analyses.

Longitudinal studies can show how migration and urbanisation influence risk factors for health conditions and access to treatment. While our study setting draws on a specific district-sized origin population, the social behaviour we observe is indicative of broader patterns throughout the region, with lessons for health transitions underway in other parts. The extent to which rural households are linked to urban-dwelling temporary migrants is not well known. It is not illuminated by the South African national census, which gives a snapshot of where people reside on census night. Many single-person or small households enumerated in urban settings are likely to be members of rural households situated elsewhere, to which they will return in times of leave or ill-health [24, 58]. The high prevalence of temporary migration from rural households in northeast South Africa, especially for young adults, illustrated with respect to the Agincourt HDSS population, reflects a typical pattern for rural Southern Africa, yet this population remains less visible and their health challenges insufficiently understood.

## Conclusions

This paper, based on data from the first wave of the MHFUS, contributes new evidence to improve our understanding of the migration and health relationship through the analysis of determinants of health service utilisation amongst internal migrants and permanent residents of a typical South African rural sub-district. Migrants and non-migrants in the study population were found to utilise health services differently, even in the presence of multiple statistical controls, with migrants interacting less readily with the health system as compared to non-migrants, and making far greater use of private sector facilities over public health services. These findings, which will be enhanced in future longitudinal follow-up rounds, offer important insights on how migrants interface with healthcare systems in transitioning contexts like South Africa. As such, the study assists in providing evidence to support the development of health and social policy to provide effective healthcare for internal migrants as part of developing locally adapted approaches to universal health coverage. Achieving the intent of the legal, political and health-systems development processes underway concerning National Health Insurance in South Africa can be greatly enhanced by recognising the as-yet-unmet needs of South Africa’s sizeable internal migrant community.

## Supplementary Information

**Additional file 1.**

## Data Availability

Data from the Migrant Health Follow-Up Study are available from the corresponding author on reasonable request. Agincourt Health and Demographic Surveillance Systems data are available through SAPRIN URL <http://saprin.mrc.ac.za/> and <http://saprindata.samrc.ac.za/>.

## Abbreviations

AIDS: Acquired immune deficiency syndrome
ATE: Average treatment effect
HDSS: Health and Demographic Surveillance System
HIV: Human immunodeficiency virus
ID: Infectious disease
Km: Kilometre
MHFUS: Migrant Health Follow-Up Study
NCD: Non-communicable disease
NS: Not significant
PSM: Propensity score matching
TB: Tuberculosis
RRR: Relative risk ratio

## Footnotes

1 We elected to exclude employment status from the final models, because of the strong collinearity between migration and employment, and the fact that these baseline data could not be temporally ordered.
