# Supplementary material for: Internal migration and health in South Africa: determinants of healthcare utilisation in a young adult cohort
Source: BMC Public Health. 2021 Mar 20;21:554. doi: 10.1186/s12889-021-10590-6 (PMC7981972; doi:10.1186/s12889-021-10590-6)
Supplement: Supplementary file 1 — Additional file 1. [file 12889_2021_10590_MOESM1_ESM.pdf]

UNIVERSITY OF THE WITWATERSRAND/BROWN UNIVERSITY

MIGRANT HEALTH FOLLOW-UP STUDY

Wave 1 Questionnaire

The following is the print version of the questionnaire as generated by the Research Electronic Data Capture software, REDCap, hosted at the University of the Witwatersrand.

Pre Interview

record_id Record ID	__________________________________
	__________________________________
Interviewer Name	◯<br>◯<br>◯<br>◯<br>◯<br>◯<br>◯<br>◯<br>◯<br>◯<br>◯
	__________________________________
Confirm that [respondent_id] is the correct respondent ID	◯ Yes<br>◯ No
Sample Type	◯ Untraceable
respondent_name	__________________________________
Confirm the respondent's name is [respondent_name].	◯ Yes<br>◯ No
If the person you are talking to is not [respondent_name], terminate the interview and locate the correct respondent.	
respondent_sex	__________________________________
Confirm the respondent's sex is [respondent_sex].	◯ Yes<br>◯ No
If the person you are talking to is not [respondent_sex], terminate the interview and locate the correct respondent.	
respondent_dob	__________________________________
Confirm the respondent's date of birth is [respondent_dob].	◯ Yes<br>◯ No
If the person you are talking to was not born on [respondent_dob], you may be talking to the wrong person. Confirm the identity of the respondent. If it is not the correct respondent, terminate the interview and locate the correct respondent.	

If you are sure the respondent is the correct respondent but their date of birth is inaccurate, please enter their actual date of birth here:	________________________
Respondent's origin location	________________________<br>(To be pre-populated from DSS data)
Where is the respondent located? What province?	◯ Mpumalanga<br>◯ Gauteng<br>◯ Limpopo<br>◯ Free State<br>◯ KwaZulu Natal<br>◯ Eastern Cape<br>◯ Western Cape<br>◯ Northern Cape<br>◯ North West<br>◯ Other
Please specify:	________________________
Where within Mpumalanga?	◯ Agincourt study site<br>◯ Barberton<br>◯ Bushbuckridge<br>◯ Dwarsloop<br>◯ Game park Mpumalanga<br>◯ Gemu / Hazyview<br>◯ Graskop/ Pilgrim's rest<br>◯ Kangwane (former homeland)<br>◯ Kiepersol<br>◯ Komatipoort<br>◯ Machadodorp<br>◯ Malelane<br>◯ Mapulaneng / Bushbuckridge<br>◯ Marite / Alexandria<br>◯ Matsulu<br>◯ Middleburg<br>◯ Mkhuhlu<br>◯ Nelspruit<br>◯ Pienaar (Nelspruit)<br>◯ Sabie<br>◯ Thulamahashe<br>◯ White River<br>◯ Witbank<br>◯ Zwelitsha<br>◯ Other Mpumalanga<br>◯ Don't know
Please specify:	________________________

Where within Gauteng

- ◯ Akasia
- ◯ Alberton
- ◯ Alexandra
- ◯ Atteridgeville
- ◯ Babelegi
- ◯ Bapsfontein
- ◯ Bashewa
- ◯ Baviaanspoort
- ◯ Bekkersdal
- ◯ Benoni
- ◯ Bhongweni
- ◯ Blue Hills
- ◯ Boipatong
- ◯ Boksburg
- ◯ Bon Accord
- ◯ Bonamanzi Marina and Country Club
- ◯ Bophelong
- ◯ Boschkop
- ◯ Bosplaas Mathabe
- ◯ Bothasgeluk
- ◯ Brakpan
- ◯ Breswol
- ◯ Bronkhorstspruit
- ◯ Bultfontein
- ◯ Carletonville
- ◯ Centurion
- ◯ Chartwell
- ◯ Cheetah Park
- ◯ Chief A Luthuli Park
- ◯ City of Johannesburg NU
- ◯ Clayville
- ◯ Cullinan
- ◯ Dainfern
- ◯ Daveyton
- ◯ Deelkraal Gold Mine
- ◯ Derdepoort
- ◯ Devon A
- ◯ Devon B
- ◯ Diepsloot
- ◯ Dilopye
- ◯ Donkerhoek
- ◯ Doornfontein
- ◯ Doornkraal
- ◯ Downbern
- ◯ Drie Ziek
- ◯ Duduza
- ◯ Dukathole
- ◯ East Driefontein
- ◯ East Village
- ◯ Ebony Park
- ◯ Edenvale
- ◯ Eersterust
- ◯ Ekangala
- ◯ Ekurhuleni NU
- ◯ Elandsfontein
- ◯ Elandsrand
- ◯ Elandsridge
- ◯ Elsburg Gold Mine
- ◯ Emfuleni NU
- ◯ Endicott
- ◯ Ennerdale
- ◯ Etwatwa
- ◯ Evaton
- ◯ Farmall
- ◯ Fochville
- ◯ Ga-Rankuwa
- ◯ Geluksdal
- ◯ Germiston
- ◯ Glen Harvie

◯ Golden Gardens
◯ Goudvlakte West
◯ Green Park
◯ Grootfontein
◯ Grootvlei
◯ Haakdoornboom
◯ Hallgate
◯ Hammanskraal
◯ Harry Gwala
◯ Hartebeesfontein
◯ Hebron
◯ Heidelberg
◯ Hillshaven
◯ Holfontein
◯ Impumelelo
◯ Itsoseng
◯ Ivory Park
◯ Johandeo
◯ Johannesburg
◯ Kaalfontein
◯ Kagiso
◯ Kameeldrift
◯ Kameelfontein
◯ Kameelkraal
◯ Kanana
◯ Kanana Park
◯ Katlehong
◯ Keinfontein
◯ Kekana Garden
◯ Kempton Park
◯ Khutsong
◯ Kleinfontein
◯ Kokosi
◯ Krugersdorp
◯ Kungwini Part 2
◯ Kwa-Thema
◯ Lakeside
◯ Langaville
◯ Lanseria
◯ Laudium
◯ Lawley
◯ Leeufontein
◯ Leeuport
◯ Lehae
◯ Lenasia
◯ Lenasia South
◯ Lenz
◯ Lesedi NU
◯ Letsatsing
◯ Lindelani Village
◯ Lucky 7
◯ Mabopane
◯ Magaliesburg
◯ Majaneng
◯ Makanyaneng
◯ Malatjie
◯ Mamello
◯ Mamelodi
◯ Mandela Village
◯ Marokolong
◯ Mashemong
◯ Mayibuye
◯ Merafong City NU
◯ Meyerton
◯ Midrand
◯ Midvaal NU
◯ Millgate Farm
◯ Mogale City NU
◯ Mohlakeng
◯ Mooiplaas
◯ Munsieville

◯ Nellmapius
◯ New Eersterus
◯ Nigel
◯ Nooitgecht
◯ Nufcor
◯ Oberholzer
◯ Olievenhoutbos
◯ Onverwacht
◯ Orange Farm
◯ Orient Hills
◯ Pebble Rock Golf Village
◯ Phomolong
◯ Poortjie
◯ Pretoria
◯ Rabie Ridge
◯ Ramotse
◯ Randburg
◯ Randfontein
◯ Randfontein NU
◯ Randvaal
◯ Ratanda
◯ Rayton
◯ Refilwe
◯ Rethabiseng
◯ Rietfontein
◯ Rietvallei
◯ Roodeplaat
◯ Roodepoort
◯ Sable Hills Waterfront Estate
◯ Sandton
◯ Saulsville
◯ Seberuberung
◯ Sebokeng
◯ Sharpeville
◯ Simunye
◯ Sonstraal
◯ Soshanguve
◯ Southdene
◯ Soutpan
◯ Soweto
◯ Spaarwater
◯ Springs
◯ Stinkwater
◯ Stretford
◯ Suurman
◯ Temba
◯ Tembisa
◯ The Carousel Casino and Entertainment World
◯ The Hill
◯ The Village
◯ Thembisile
◯ Thinasonke
◯ Tierpoort
◯ Tokoza
◯ Tsakane
◯ Tsebe
◯ Tshepiso
◯ Tshepisong
◯ Tshwane NU
◯ Tweedracht
◯ Tweefontein
◯ Vaal Marina
◯ Vaal Oewer
◯ Vaalbank
◯ Vanderbijlpark
◯ Vastfontein
◯ Venterspost
◯ Vereeniging
◯ Viskuil
◯ Vlakfontein
◯ Vosloorus

◯ Wageenbietjieskop
◯ Walkerville
◯ Wallis Haven
◯ Wallmannsthal
◯ Waterpan
◯ Waterval
◯ Waterworks
◯ Wattville
◯ Wedela
◯ Welverdiend
◯ West Village
◯ West-Driefontein
◯ Western Areas Gold Mine
◯ Western Deep Levels Mine
◯ Westonaria
◯ Westonaria NU
◯ Winterveld
◯ Wolwekrans
◯ Zakariyya Park
◯ Zenzele
◯ Zevenfontein
◯ Zithobeni
◯ Zwartkop
◯ Zwavelpoort
◯ Other
◯ Don't Know

Please specify:

____

Where within Limpopo?

◯ Aganang
◯ Ba-Phalaborwa
◯ Bela-Bela
◯ Blouberg
◯ Elias Motsoaledi
◯ Fetakgomo
◯ Greater Giyani
◯ Greater Letaba
◯ Greater Marble Hall
◯ Greater Tubatse
◯ Greater Tzaneen
◯ Lepelle-Nkumpi
◯ Lephalale
◯ Makhado
◯ Makhuduthamaga
◯ Malamulele
◯ Maruleng
◯ Modimolle
◯ Mogalakwena
◯ Molemole
◯ Mookgopong
◯ Musina
◯ Polokwane
◯ Thabazimbi
◯ Thulamela
◯ Other
◯ Don't know

Please specify:

____

Where within Western Cape?

____

Where within Eastern Cape?	__________________________________
Where within North West?	__________________________________
Where within KwaZulu Natal?	__________________________________
Where within Northern Cape?	__________________________________
Where within Free State?	__________________________________
Is it rural or urban?	◯ Rural<br>◯ Urban (city or town)<br>◯ Don't know
Latitude	__________________________________
Longitude	__________________________________
Where is the interview taking place?	◯ Respondent's home<br>◯ Respondent's place of work<br>◯ A location near the respondent's home<br>◯ A location near the respondent's work<br>◯ Telephone Interview<br>◯ Other
Please specify:	__________________________________

Consent

	______________________
interview_consent Has the participant read, signed, and dated the informed consent for the interview?	◯ Yes<br>◯ No
interview_consent_no For our own purposes, do you mind telling us why you have chosen to not sign the informed consent?	______________________
anthro_consent Has the participant read, signed, and dated the informed consent for the collection of the anthropometric measures?	◯ Yes<br>◯ No
dbs_consent Has the participant read, signed, and dated the informed consent for the collection of the dry blood spot to test for blood sugar	◯ Yes<br>◯ No
dbs_consent Has the participant read, signed, and dated the informed consent for the collection of the dry blood spot to test for HIV	◯ Yes<br>◯ No
hiv_referral Has the participant indicated they want a referral for HIV testing?	◯ Yes<br>◯ No
Self-test provided?	◯ Yes<br>◯ No
Has the participant informed consent been signed and dated by fieldworker?	◯ Yes<br>◯ No
Has the participant been given the information sheet?	◯ Yes<br>◯ No

Education and Employment

____

Form Status

Interview date and start time ______________________________

Now I am going to ask you some questions about your educational history

Are you currently enrolled in school/completing any studies/training/certificates?
- ◯ Yes
- ◯ No
- ◯ Don't know

In what level of education are you currently engaged?
- ◯ Std 1/Grade 3
- ◯ Std 2/Grade 4
- ◯ Std 3/Grade 5
- ◯ Std 4/Grade 6
- ◯ Std 5/Grade 7
- ◯ Std 6/Grade 8
- ◯ Std 7/Grade 9
- ◯ Std 8/Grade 10
- ◯ Std 9/Grade 11
- ◯ Std 10/Matric
- ◯ Technicon- Incomplete
- ◯ Technicon-Complete
- ◯ University - Incomplete
- ◯ University - Complete
- ◯ Adult Basic Education and Training Level 1 (ABET 1)
- ◯ ABET 2
- ◯ ABET 3
- ◯ ABET 4
- ◯ National Qualification Framework Level 1 (NQF 1)
- ◯ NQF 2
- ◯ NQF 3
- ◯ NQF 4
- ◯ Other certificate or diploma
- ◯ Other
- ◯ Don't know

Please specify ______________________________

Are these studies full or part-time?
- ◯ Full-time
- ◯ Part-time
- ◯ Don't know

What is the highest level of education you have completed?	◯ Std 1/Grade 3<br>◯ Std 2/Grade 4<br>◯ Std 3/Grade 5<br>◯ Std 4/Grade 6<br>◯ Std 5/Grade 7<br>◯ Std 6/Grade 8<br>◯ Std 7/Grade 9<br>◯ Std 8/Grade 10<br>◯ Std 9/Grade 11<br>◯ Std 10/Matric<br>◯ Technicon- Incomplete<br>◯ Technicon-Complete<br>◯ University - Incomplete<br>◯ University - Complete<br>◯ Adult Basic Education and Training Level 1 (ABET 1)<br>◯ ABET 2<br>◯ ABET 3<br>◯ ABET 4<br>◯ National Qualification Framework Level 1 (NQF 1)<br>◯ NQF 2<br>◯ NQF 3<br>◯ NQF 4<br>◯ Other certificate or diploma<br>◯ Other<br>◯ Don't know
Please specify	______________________________
Have you passed matric?	◯ Yes<br>◯ No

Now I want to ask you about work. Work is any activity that brings money and other resources into the household from outside and includes formal and informal work and trading.

What is your employment status?	◯ Unemployed, not looking for work<br>◯ Unemployed, looking for work<br>◯ Employed full time, formal sector<br>◯ Employed part time, formal sector<br>◯ Employed full time, informal sector<br>◯ Employed part time, informal sector<br>◯ Self-employed, formal sector<br>◯ Self-employed, informal sector

What type of work are you doing?	◯ Farm work<br>◯ Domestic work<br>◯ Construction work<br>◯ Security work<br>◯ Cleaning work<br>◯ Small business owner<br>◯ Mine work<br>◯ Teacher<br>◯ Traditional healer<br>◯ Health sector (formal)<br>◯ Game farm/game reserve (e.g. ranger)<br>◯ Driver<br>◯ Skilled worker (e.g. plumber, mechanic, electrician)<br>◯ Cook/ chef/ catering<br>◯ Unskilled worker (e.g. general labourer)<br>◯ Artisan (e.g. carpenter, wood carver, weaver)<br>◯ Waiter/ barman<br>◯ Informal selling<br>◯ Small business assistant<br>◯ Clerical and office work<br>◯ Cattle herder<br>◯ Sewing, hairdressing, baking, brewing<br>◯ Police, soldier, fireman<br>◯ Petrol attendant<br>◯ Timber, sawmill, poles<br>◯ Gardening services<br>◯ Fieldworker - NGO or university<br>◯ Art, craft, photography, fashion design<br>◯ Senior administrator, manager, professional<br>◯ Priest/pastor<br>◯ Retail/clerk/sales<br>◯ Student<br>◯ Other<br>◯ Don't know<br>(If unsure of how to classify, select "Other")
Please specify	__________________________________
Is your employment permanent, fixed/period or contract work, or occasional/irregular?	◯ Permanent<br>◯ Fixed period/Contract work<br>◯ Occasional/Irregular<br>◯ Don't know
On average, how many hours do you work in a typical week?	◯ 1-10<br>◯ 11-20<br>◯ 21-30<br>◯ 31-40<br>◯ 41-50<br>◯ 51-60<br>◯ 61-70<br>◯ 71-80<br>◯ 81 or more

How much did you (as an individual) earn in total last month?	◯ No income<br>◯ R1 - R400<br>◯ R401 - R800<br>◯ R 801 - R1 600<br>◯ R1 601 - R3 200<br>◯ R3 201 - R6 400<br>◯ R6 401 - R12 800<br>◯ R12 801 - R19 200<br>◯ R19 201 - R25 600<br>◯ R25 601 - R38 400<br>◯ R38 401 - R51 200<br>◯ R51 201 - R76 800<br>◯ R76 801 - R102 400<br>◯ R102 401 - R153 600<br>◯ R153 601 - R204 800<br>◯ R204 801 - R500 000<br>◯ More than R500 000<br>◯ Don't know<br>◯ Respondent refused<br>(This means take home salary)

Mark as "Complete" and select "Save and go to next instrument" when complete

Current Residence

CURRENT RESIDENCE

I want to ask you some questions about your residence and employment history. We will start with your current place of residence.

Where do you currently live? Please be specific: ______________________

If possible, collect the address or detailed location of the respondent's current residence: ______________________

What province is [current_residence] in?
- ◯ Mpumalanga
- ◯ Gauteng
- ◯ Limpopo
- ◯ Free State
- ◯ KwaZulu Natal
- ◯ Eastern Cape
- ◯ Western Cape
- ◯ Northern Cape
- ◯ North West
- ◯ Other country

Please specify: ______________________

Where within Mpumalanga is [current_residence]?

- ◯ Agincourt study site
- ◯ Barberton
- ◯ Bushbuckridge
- ◯ Dwarsloop
- ◯ Game park Mpumalanga
- ◯ Gemu / Hazyview
- ◯ Graskop/ Pilgrim's rest
- ◯ Kangwane (former homeland)
- ◯ Kiepersol
- ◯ Komatipoort
- ◯ Machadodorp
- ◯ Malelane
- ◯ Mapulaneng / Bushbuckridge
- ◯ Marite / Alexandria
- ◯ Matsulu
- ◯ Middleburg
- ◯ Mkhuhlu
- ◯ Nelspruit
- ◯ Pienaar (Nelspruit)
- ◯ Sabie
- ◯ Thulamahashe
- ◯ White River
- ◯ Witbank
- ◯ Zwelitsha
- ◯ Other Mpumalanga
- ◯ Don't know

Please specify: ______________________________

Where within Gauteng is [current_residence]?

◯ Akasia
◯ Alberton
◯ Alexandra
◯ Atteridgeville
◯ Babelegi
◯ Bapsfontein
◯ Bashewa
◯ Baviaanspoort
◯ Bekkersdal
◯ Benoni
◯ Bhongweni
◯ Boipatong
◯ Boksburg
◯ Bon Accord
◯ Bophelong
◯ Boschkop
◯ Bosplaas Mathabe
◯ Bothasgeluk
◯ Brakpan
◯ Breswol
◯ Bronkhorstspruit
◯ Bultfontein
◯ Carletonville
◯ Centurion
◯ Chartwell
◯ Cheetah Park
◯ Chief A Luthuli Park
◯ City of Johannesburg
◯ Clayville
◯ Cullinan
◯ Dainfern
◯ Daveyton
◯ Deelkraal Gold Mine
◯ Derdepoort
◯ Devon A
◯ Devon B
◯ Diepsloot
◯ Dilopye
◯ Donkerhoek
◯ Doornfontein
◯ Doornkraal
◯ Downbern
◯ Drie Ziek
◯ Duduza
◯ Dukathole
◯ East Driefontein
◯ East Village
◯ Ebony Park
◯ Edenvale
◯ Eersterust
◯ Ekangala
◯ Ekurhuleni
◯ Elandsfontein
◯ Elandsrand
◯ Elandsridge
◯ Elsburg Gold Mine
◯ Emfuleni
◯ Endicott
◯ Ennerdale
◯ Etwatwa
◯ Evaton
◯ Farmall
◯ Fochville
◯ Ga-Rankuwa
◯ Geluksdal
◯ Germiston
◯ Glen Harvie
◯ Golden Gardens
◯ Goudvlakte West

◯ Green Park
◯ Grootfontein
◯ Grootvlei
◯ Haakdoornboom
◯ Hallgate
◯ Hammanskraal
◯ Harry Gwala
◯ Hartebeesfontein
◯ Hebron
◯ Heidelberg
◯ Hillshaven
◯ Impumelelo
◯ Itsoseng
◯ Ivory Park
◯ Johandeo
◯ Johannesburg
◯ Kaalfontein
◯ Kagiso
◯ Kameeldrift
◯ Kameelfontein
◯ Kameelkraal
◯ Kanana
◯ Kanana Park
◯ Katlehong
◯ Keinfontein
◯ Kekana Garden
◯ Kempton Park
◯ Khutsong
◯ Kleinfontein
◯ Kokosi
◯ Krugersdorp
◯ Kungwini Part 2
◯ Kwa-Thema
◯ Lakeside
◯ Langaville
◯ Lanseria
◯ Laudium
◯ Lawley
◯ Leeufontein
◯ Leeuport
◯ Lehae
◯ Lenasia
◯ Lenasia South
◯ Lenz
◯ Lesedi
◯ Letsatsing
◯ Lindelani Village
◯ Mabopane
◯ Magaliesburg
◯ Majaneng
◯ Makanyaneng
◯ Malatjie
◯ Mamello
◯ Mamelodi
◯ Mandela Village
◯ Marokolong
◯ Mashemong
◯ Mayibuye
◯ Merafong City
◯ Meyerton
◯ Midrand
◯ Midvaal
◯ Millgate Farm
◯ Mogale City
◯ Mohlakeng
◯ Mooiplaas
◯ Munsieville
◯ Nellmapius
◯ New Eersterus
◯ Nigel
◯ Nooitgecht

◯ Nufcor
◯ Oberholzer
◯ Olievenhoutbos
◯ Onverwacht
◯ Orange Farm
◯ Orient Hills
◯ Other Gauteng
◯ Pebble Rock Golf Village
◯ Phomolong
◯ Poortjie
◯ Pretoria
◯ Rabie Ridge
◯ Ramotse
◯ Randburg
◯ Randfontein
◯ Randvaal
◯ Ratanda
◯ Rayton
◯ Refilwe
◯ Rethabiseng
◯ Rietfontein
◯ Rietvallei
◯ Roodeplaat
◯ Roodepoort
◯ Sable Hills Waterfront Estate
◯ Sandton
◯ Saulsville
◯ Seberuberung
◯ Sebokeng
◯ Sharpeville
◯ Simunye
◯ Sonstraal
◯ Soshanguve
◯ Southdene
◯ Soutpan
◯ Soweto
◯ Spaarwater
◯ Springs
◯ Stinkwater
◯ Stretford
◯ Suurman
◯ Temba
◯ Tembisa
◯ The Hill
◯ The Village
◯ Thembisile
◯ Thinasonke
◯ Tierpoort
◯ Tokoza
◯ Tsakane
◯ Tsebe
◯ Tshepiso
◯ Tshepisong
◯ Tshwane
◯ Tweedracht
◯ Tweefontein
◯ Vaal Marina
◯ Vaal Oewer
◯ Vaalbank
◯ Vanderbijlpark
◯ Vastfontein
◯ Venterspost
◯ Vereeniging
◯ Viskuil
◯ Vlakfontein
◯ Vosloorus
◯ Wageenbietjieskop
◯ Walkerville
◯ Wallis Haven
◯ Wallmannsthal
◯ Waterpan

- ◯ Waterval
- ◯ Waterworks
- ◯ Wattville
- ◯ Wedela
- ◯ Welverdiend
- ◯ West Village
- ◯ West-Driefontein
- ◯ Western Areas Gold Mine
- ◯ Western Deep Levels Mine
- ◯ Westonaria
- ◯ Winterveld
- ◯ Wolwekrans
- ◯ Zakariyya Park
- ◯ Zenzele
- ◯ Zithobeni
- ◯ Zwartkop
- ◯ Zwavelpoort
- ◯ Other Gauteng
- ◯ Don't know

Please specify: ________________________________

Where within Limpopo is [current_residence]?

- ◯ Aganang
- ◯ Ba-Phalaborwa
- ◯ Bela-Bela
- ◯ Blouberg
- ◯ Elias Motsoaledi
- ◯ Fetakgomo
- ◯ Greater Giyani
- ◯ Greater Letaba
- ◯ Greater Marble Hall
- ◯ Greater Tubatse
- ◯ Greater Tzaneen
- ◯ Lepelle-Nkumpi
- ◯ Lephalale
- ◯ Makhado
- ◯ Makhuduthamaga
- ◯ Malamulele
- ◯ Maruleng
- ◯ Modimolle
- ◯ Mogalakwena
- ◯ Molemole
- ◯ Mookgopong
- ◯ Musina
- ◯ Polokwane
- ◯ Thabazimbi
- ◯ Thulamela
- ◯ Other
- ◯ Don't know

Please specify: ________________________________

Where within Western Cape is [current_residence]? ________________________________

Where within Eastern Cape is [current_residence]? ________________________________

Where within North West is [current_residence]? ________________________________

Where within KwaZulu Natal is [current_residence]?	__________________________________
Where within Northern Cape is [current_residence]?	__________________________________
Where within Free State is [current_residence]?	__________________________________
Is it rural or urban?	◯ Rural<br>◯ Urban (city or town)<br>◯ Don't know<br>(Rural meaning locations like villages, agricultural areas, or game farms)
When did you start living here? Enter MONTH	◯ January<br>◯ February<br>◯ March<br>◯ April<br>◯ May<br>◯ June<br>◯ July<br>◯ August<br>◯ September<br>◯ October<br>◯ November<br>◯ December<br>◯ Don't know

When did you start living here? Enter YEAR	◯ 2019<br>◯ 2018<br>◯ 2017<br>◯ 2016<br>◯ 2015<br>◯ 2014<br>◯ 2013<br>◯ 2012<br>◯ 2011<br>◯ 2010<br>◯ 2009<br>◯ 2008<br>◯ 2007<br>◯ 2006<br>◯ 2005<br>◯ 2004<br>◯ 2003<br>◯ 2002<br>◯ 2001<br>◯ 2000<br>◯ 1999<br>◯ 1998<br>◯ 1997<br>◯ 1996<br>◯ 1995<br>◯ 1994<br>◯ 1993<br>◯ 1992<br>◯ 1991<br>◯ 1990<br>◯ 1989<br>◯ 1988<br>◯ 1987<br>◯ 1986<br>◯ 1985<br>◯ 1984<br>◯ 1983<br>◯ 1982<br>◯ 1981<br>◯ 1980<br>◯ Don't Know
In what type of place do you live?	◯ Commercial farm<br>◯ Game farm/nature reserve<br>◯ Mine<br>◯ Military base<br>◯ City (inner)<br>◯ City (suburb)<br>◯ Town<br>◯ Village (trust land)<br>◯ Informal settlement (city)<br>◯ Informal settlement (rural)<br>◯ Township (urban)<br>◯ Township (rural)<br>◯ Other<br>◯ Don't know
Please specify	__________________________

What type of dwelling do you live in?	◯ House, brick or concrete structure on a separate stand<br>◯ Traditional dwelling, hut or structure made of traditional materials<br>◯ Flat or apartment in a block of flats/complex<br>◯ Cluster house in a complex<br>◯ Townhouse<br>◯ Semi-detached house not in a complex<br>◯ House, flat or room separate from main dwelling in backyard<br>◯ Informal dwelling or shack in backyard<br>◯ Informal dwelling NOT in backyard, e.g. in informal squatter settlement or on a farm<br>◯ Room or flat which is part of main dwelling or property<br>◯ Caravan or tent<br>◯ Unit in a retirement home or barracks etc.<br>◯ Other<br>◯ Don't know
Please specify	__________________________________
Not including yourself, how many household members are there at this residence?	__________________________________<br>(For these questions we mean where the respondent is CURRENTLY LIVING. By household members, we mean people living in the same physical structure as the respondent, who they share most meals with and/or share resources with.)
FOR FIELDWORKER: Is the repsondent's origin location ([q_1_5]) the same as their current residence ([current_residence])?	◯ Yes<br>◯ No
Are you the head of the household?	◯ Yes<br>◯ No<br>(The head of household is a person who is responsible for generating and managing the largest part of the household income)
Who is the current head of the household?	__________________________________<br>(For these questions we meant where the respondent is CURRENTLY LIVING. The head of household is a person who is responsible for generating and managing the largest part of the household income)
How many household members are under 18 years old?	__________________________________
How many household members are over 18 years old?	__________________________________<br>(This should include the respondent)
	__________________________________
Note to interviewer: Total is [q_4_9_4]. Total should be [q_4_9_3] .	__________________________________

Mark as "Complete" and select "Save and go to next instrument" when complete.

H H Roster

Repeat for all [q_4_7] other household members

HOUSEHOLD ROSTER

(1) Name of household member: ________________

Start with the head of the household (if not the respondent) and then those closest to the respondent. Include the name and surname of all household members.

(1) Is [q_5_11] the household head?
- ◯ Yes
- ◯ No

(1) Is [q_5_11] male or female?
- ◯ Male
- ◯ Female

(1) What is [q_5_11]'s relation to you?
- ◯ Mother
- ◯ Father
- ◯ Brother
- ◯ Sister
- ◯ Spouse/partner/boyfriend/girlfriend
- ◯ Son
- ◯ Daughter
- ◯ Maternal Grandmother
- ◯ Maternal Grandfather
- ◯ Paternal Grandmother
- ◯ Paternal Grandfather
- ◯ Maternal Aunt
- ◯ Maternal Uncle
- ◯ Paternal Aunt
- ◯ Paternal Uncle
- ◯ Niece
- ◯ Nephew
- ◯ Friend
- ◯ Countrymen/Co-worker
- ◯ Mother-in-law
- ◯ Father-in-law
- ◯ Brother-in-law
- ◯ Sister-in-law
- ◯ Son-in-law
- ◯ Daughter-in-law
- ◯ Other

(1) Please specify: ________________

(1) What is [q_5_11]'s occupation?	◯ None/Unemployed (receive social grant)<br>◯ None/Unemployed (do not receive social grant)<br>◯ Farm work<br>◯ Domestic work<br>◯ Construction work<br>◯ Security work<br>◯ Cleaning work<br>◯ Small business owner<br>◯ Mine work<br>◯ Teacher<br>◯ Traditional healer<br>◯ Health sector (formal)<br>◯ Game farm/game reserve (e.g. ranger)<br>◯ Driver<br>◯ Skilled worker (e.g. plumber, mechanic, electrician)<br>◯ Cook/ chef/ catering<br>◯ Unskilled worker (e.g. general labourer)<br>◯ Artisan (e.g. carpenter, wood carver, weaver)<br>◯ Waiter/ barman<br>◯ Informal selling<br>◯ Small business assistant<br>◯ Clerical and office work<br>◯ Cattle herder<br>◯ Sewing, hairdressing, baking, brewing<br>◯ Police, soldier, fireman<br>◯ Petrol attendant<br>◯ Timber, sawmill, poles<br>◯ Gardening services<br>◯ Fieldworker - NGO or university<br>◯ Art, craft, photography, fashion design<br>◯ Senior administrator, manager, professional<br>◯ Priest/pastor<br>◯ Retail/clerk/sales<br>◯ Student<br>◯ Other<br>◯ Don't know
(1) Please specify:	______________________________
(1) How old is [q_5_11]?	______________________________
(2) Name of household member:	______________________________<br>(Include Name and Surname.)
(2) Is [q_5_12] the household head?	◯ Yes<br>◯ No
(2) Is [q_5_12] male or female?	◯ Male<br>◯ Female

(2) What is [q_5_12]'s relation to you?

- ◯ Mother
- ◯ Father
- ◯ Brother
- ◯ Sister
- ◯ Spouse/partner/boyfriend/girlfriend
- ◯ Son
- ◯ Daughter
- ◯ Maternal Grandmother
- ◯ Maternal Grandfather
- ◯ Paternal Grandmother
- ◯ Paternal Grandfather
- ◯ Maternal Aunt
- ◯ Maternal Uncle
- ◯ Paternal Aunt
- ◯ Paternal Uncle
- ◯ Niece
- ◯ Nephew
- ◯ Friend
- ◯ Countrymen/Co-worker
- ◯ Mother-in-law
- ◯ Father-in-law
- ◯ Brother-in-law
- ◯ Sister-in-law
- ◯ Son-in-law
- ◯ Daughter-in-law
- ◯ Other

(2) Please specify:

____

(2) What is [q_5_12]'s occupation?	◯ None/Unemployed (receive social grant)<br>◯ None/Unemployed (do not receive social grant)<br>◯ Farm work<br>◯ Domestic work<br>◯ Construction work<br>◯ Security work<br>◯ Cleaning work<br>◯ Small business owner<br>◯ Mine work<br>◯ Teacher<br>◯ Traditional healer<br>◯ Health sector (formal)<br>◯ Game farm/game reserve (e.g. ranger)<br>◯ Driver<br>◯ Skilled worker (e.g. plumber, mechanic, electrician)<br>◯ Cook/ chef/ catering<br>◯ Unskilled worker (e.g. general labourer)<br>◯ Artisan (e.g. carpenter, wood carver, weaver)<br>◯ Waiter/ barman<br>◯ Informal selling<br>◯ Small business assistant<br>◯ Clerical and office work<br>◯ Cattle herder<br>◯ Sewing, hairdressing, baking, brewing<br>◯ Police, soldier, fireman<br>◯ Petrol attendant<br>◯ Timber, sawmill, poles<br>◯ Gardening services<br>◯ Fieldworker - NGO or university<br>◯ Art, craft, photography, fashion design<br>◯ Senior administrator, manager, professional<br>◯ Priest/pastor<br>◯ Retail/clerk/sales<br>◯ Other<br>◯ Don't know
(2) Please specify:	__________________________
(2) How old is [q_5_12]?	__________________________
(3) Name of household member:	__________________________<br>(Include Name and Surname.)
(3) Is [q_5_13] the household head?	◯ Yes<br>◯ No
(3) Is [q_5_13] male or female?	◯ Male<br>◯ Female

(3) What is [q_5_13]'s relation to you?

- ◯ Mother
- ◯ Father
- ◯ Brother
- ◯ Sister
- ◯ Spouse/partner/boyfriend/girlfriend
- ◯ Son
- ◯ Daughter
- ◯ Maternal Grandmother
- ◯ Maternal Grandfather
- ◯ Paternal Grandmother
- ◯ Paternal Grandfather
- ◯ Maternal Aunt
- ◯ Maternal Uncle
- ◯ Paternal Aunt
- ◯ Paternal Uncle
- ◯ Niece
- ◯ Nephew
- ◯ Friend
- ◯ Countrymen/Co-worker
- ◯ Mother-in-law
- ◯ Father-in-law
- ◯ Brother-in-law
- ◯ Sister-in-law
- ◯ Son-in-law
- ◯ Daughter-in-law
- ◯ Other

(3) Please specify: ____________________________

(3) What is [q_5_13]'s occupation?	◯ None/Unemployed (receive social grant)<br>◯ None/Unemployed (do not receive social grant)<br>◯ Farm work<br>◯ Domestic work<br>◯ Construction work<br>◯ Security work<br>◯ Cleaning work<br>◯ Small business owner<br>◯ Mine work<br>◯ Teacher<br>◯ Traditional healer<br>◯ Health sector (formal)<br>◯ Game farm/game reserve (e.g. ranger)<br>◯ Driver<br>◯ Skilled worker (e.g. plumber, mechanic, electrician)<br>◯ Cook/ chef/ catering<br>◯ Unskilled worker (e.g. general labourer)<br>◯ Artisan (e.g. carpenter, wood carver, weaver)<br>◯ Waiter/ barman<br>◯ Informal selling<br>◯ Small business assistant<br>◯ Clerical and office work<br>◯ Cattle herder<br>◯ Sewing, hairdressing, baking, brewing<br>◯ Police, soldier, fireman<br>◯ Petrol attendant<br>◯ Timber, sawmill, poles<br>◯ Gardening services<br>◯ Fieldworker - NGO or university<br>◯ Art, craft, photography, fashion design<br>◯ Senior administrator, manager, professional<br>◯ Priest/pastor<br>◯ Retail/clerk/sales<br>◯ Student<br>◯ Other<br>◯ Don't know
(3) Please specify:	______________________________
(3) How old is [q_5_13]?	______________________________
(4) Name of household member:	______________________________<br>(Include Name and Surname.)
(4) Is [q_5_14] the household head?	◯ Yes<br>◯ No
(4) Is [q_5_14] male or female?	◯ Male<br>◯ Female

(4) What is [q_5_14]'s relation to you?	◯ Mother<br>◯ Father<br>◯ Brother<br>◯ Sister<br>◯ Spouse/partner/boyfriend/girlfriend<br>◯ Son<br>◯ Daughter<br>◯ Maternal Grandmother<br>◯ Maternal Grandfather<br>◯ Paternal Grandmother<br>◯ Paternal Grandfather<br>◯ Maternal Aunt<br>◯ Maternal Uncle<br>◯ Paternal Aunt<br>◯ Paternal Uncle<br>◯ Niece<br>◯ Nephew<br>◯ Friend<br>◯ Countrymen/Co-worker<br>◯ Mother-in-law<br>◯ Father-in-law<br>◯ Brother-in-law<br>◯ Sister-in-law<br>◯ Son-in-law<br>◯ Daughter-in-law<br>◯ Other
(4) Please specify:	______________________________

(4) What is [q_5_14]'s occupation?	◯ None/Unemployed (receive social grant)<br>◯ None/Unemployed (do not receive social grant)<br>◯ Farm work<br>◯ Domestic work<br>◯ Construction work<br>◯ Security work<br>◯ Cleaning work<br>◯ Small business owner<br>◯ Mine work<br>◯ Teacher<br>◯ Traditional healer<br>◯ Health sector (formal)<br>◯ Game farm/game reserve (e.g. ranger)<br>◯ Driver<br>◯ Skilled worker (e.g. plumber, mechanic, electrician)<br>◯ Cook/ chef/ catering<br>◯ Unskilled worker (e.g. general labourer)<br>◯ Artisan (e.g. carpenter, wood carver, weaver)<br>◯ Waiter/ barman<br>◯ Informal selling<br>◯ Small business assistant<br>◯ Clerical and office work<br>◯ Cattle herder<br>◯ Sewing, hairdressing, baking, brewing<br>◯ Police, soldier, fireman<br>◯ Petrol attendant<br>◯ Timber, sawmill, poles<br>◯ Gardening services<br>◯ Fieldworker - NGO or university<br>◯ Art, craft, photography, fashion design<br>◯ Senior administrator, manager, professional<br>◯ Priest/pastor<br>◯ Retail/clerk/sales<br>◯ Student<br>◯ Other<br>◯ Don't know
(4) Please specify:	__________________________
(4) How old is [q_5_14]?	__________________________
(5) Name of household member:	__________________________<br>(Include Name and Surname.)
(5) Is [q_5_15] the household head?	◯ Yes<br>◯ No
(5) Is [q_5_15] male or female?	◯ Male<br>◯ Female

(5) What is [q_5_15]'s relation to you?

- ◯ Mother
- ◯ Father
- ◯ Brother
- ◯ Sister
- ◯ Spouse/partner/boyfriend/girlfriend
- ◯ Son
- ◯ Daughter
- ◯ Maternal Grandmother
- ◯ Maternal Grandfather
- ◯ Paternal Grandmother
- ◯ Paternal Grandfather
- ◯ Maternal Aunt
- ◯ Maternal Uncle
- ◯ Paternal Aunt
- ◯ Paternal Uncle
- ◯ Niece
- ◯ Nephew
- ◯ Friend
- ◯ Countrymen/Co-worker
- ◯ Mother-in-law
- ◯ Father-in-law
- ◯ Brother-in-law
- ◯ Sister-in-law
- ◯ Son-in-law
- ◯ Daughter-in-law
- ◯ Other

(5) Please specify: ______________________________

(5) What is [q_5_15]'s occupation?	◯ None/Unemployed (receive social grant)<br>◯ None/Unemployed (do not receive social grant)<br>◯ Farm work<br>◯ Domestic work<br>◯ Construction work<br>◯ Security work<br>◯ Cleaning work<br>◯ Small business owner<br>◯ Mine work<br>◯ Teacher<br>◯ Traditional healer<br>◯ Health sector (formal)<br>◯ Game farm/game reserve (e.g. ranger)<br>◯ Driver<br>◯ Skilled worker (e.g. plumber, mechanic, electrician)<br>◯ Cook/ chef/ catering<br>◯ Unskilled worker (e.g. general labourer)<br>◯ Artisan (e.g. carpenter, wood carver, weaver)<br>◯ Waiter/ barman<br>◯ Informal selling<br>◯ Small business assistant<br>◯ Clerical and office work<br>◯ Cattle herder<br>◯ Sewing, hairdressing, baking, brewing<br>◯ Police, soldier, fireman<br>◯ Petrol attendant<br>◯ Timber, sawmill, poles<br>◯ Gardening services<br>◯ Fieldworker - NGO or university<br>◯ Art, craft, photography, fashion design<br>◯ Senior administrator, manager, professional<br>◯ Priest/pastor<br>◯ Retail/clerk/sales<br>◯ Student<br>◯ Other<br>◯ Don't know
(5) Please specify:	__________________________________
(5) How old is [q_5_15]?	__________________________________
(6) Name of household member:	__________________________________<br>(Include Name and Surname.)
(6) Is [q_5_16] the household head?	◯ Yes<br>◯ No
(6) Is [q_5_16] male or female?	◯ Male<br>◯ Female

(6) What is [q_5_16]'s relation to you?

- ◯ Mother
- ◯ Father
- ◯ Brother
- ◯ Sister
- ◯ Spouse/partner/boyfriend/girlfriend
- ◯ Son
- ◯ Daughter
- ◯ Maternal Grandmother
- ◯ Maternal Grandfather
- ◯ Paternal Grandmother
- ◯ Paternal Grandfather
- ◯ Maternal Aunt
- ◯ Maternal Uncle
- ◯ Paternal Aunt
- ◯ Paternal Uncle
- ◯ Niece
- ◯ Nephew
- ◯ Friend
- ◯ Countrymen/Co-worker
- ◯ Mother-in-law
- ◯ Father-in-law
- ◯ Brother-in-law
- ◯ Sister-in-law
- ◯ Son-in-law
- ◯ Daughter-in-law
- ◯ Other

(6) Please specify: ______________________________

(6) What is [q_5_16]'s occupation?	◯ None/Unemployed (receive social grant)<br>◯ None/Unemployed (do not receive social grant)<br>◯ Farm work<br>◯ Domestic work<br>◯ Construction work<br>◯ Security work<br>◯ Cleaning work<br>◯ Small business owner<br>◯ Mine work<br>◯ Teacher<br>◯ Traditional healer<br>◯ Health sector (formal)<br>◯ Game farm/game reserve (e.g. ranger)<br>◯ Driver<br>◯ Skilled worker (e.g. plumber, mechanic, electrician)<br>◯ Cook/ chef/ catering<br>◯ Unskilled worker (e.g. general labourer)<br>◯ Artisan (e.g. carpenter, wood carver, weaver)<br>◯ Waiter/ barman<br>◯ Informal selling<br>◯ Small business assistant<br>◯ Clerical and office work<br>◯ Cattle herder<br>◯ Sewing, hairdressing, baking, brewing<br>◯ Police, soldier, fireman<br>◯ Petrol attendant<br>◯ Timber, sawmill, poles<br>◯ Gardening services<br>◯ Fieldworker - NGO or university<br>◯ Art, craft, photography, fashion design<br>◯ Senior administrator, manager, professional<br>◯ Priest/pastor<br>◯ Retail/clerk/sales<br>◯ Other<br>◯ Don't know
(6) Please specify:	______________________________
(6) How old is [q_5_16]?	______________________________
(7) Name of household member:	______________________________<br>(Include Name and Surname.)
(7) Is [q_5_17] the household head?	◯ Yes<br>◯ No
(7) Is [q_5_17] male or female?	◯ Male<br>◯ Female

(7) What is [q_5_17]'s relation to you?

- ◯ Mother
- ◯ Father
- ◯ Brother
- ◯ Sister
- ◯ Spouse/partner/boyfriend/girlfriend
- ◯ Son
- ◯ Daughter
- ◯ Maternal Grandmother
- ◯ Maternal Grandfather
- ◯ Paternal Grandmother
- ◯ Paternal Grandfather
- ◯ Maternal Aunt
- ◯ Maternal Uncle
- ◯ Paternal Aunt
- ◯ Paternal Uncle
- ◯ Niece
- ◯ Nephew
- ◯ Friend
- ◯ Countrymen/Co-worker
- ◯ Mother-in-law
- ◯ Father-in-law
- ◯ Brother-in-law
- ◯ Sister-in-law
- ◯ Son-in-law
- ◯ Daughter-in-law
- ◯ Other

(7) Please specify:

____

(7) What is [q_5_17]'s occupation?	◯ None/Unemployed (receive social grant)<br>◯ None/Unemployed (do not receive social grant)<br>◯ Farm work<br>◯ Domestic work<br>◯ Construction work<br>◯ Security work<br>◯ Cleaning work<br>◯ Small business owner<br>◯ Mine work<br>◯ Teacher<br>◯ Traditional healer<br>◯ Health sector (formal)<br>◯ Game farm/game reserve (e.g. ranger)<br>◯ Driver<br>◯ Skilled worker (e.g. plumber, mechanic, electrician)<br>◯ Cook/ chef/ catering<br>◯ Unskilled worker (e.g. general labourer)<br>◯ Artisan (e.g. carpenter, wood carver, weaver)<br>◯ Waiter/ barman<br>◯ Informal selling<br>◯ Small business assistant<br>◯ Clerical and office work<br>◯ Cattle herder<br>◯ Sewing, hairdressing, baking, brewing<br>◯ Police, soldier, fireman<br>◯ Petrol attendant<br>◯ Timber, sawmill, poles<br>◯ Gardening services<br>◯ Fieldworker - NGO or university<br>◯ Art, craft, photography, fashion design<br>◯ Senior administrator, manager, professional<br>◯ Priest/pastor<br>◯ Retail/clerk/sales<br>◯ Other<br>◯ Don't know
(7) Please specify:	__________________________________
(7) How old is [q_5_17]?	__________________________________
(8) Name of household member:	__________________________________<br>(Include Name and Surname.)
(8) Is [q_5_18] the household head?	◯ Yes<br>◯ No
(8) Is [q_5_18] male or female?	◯ Male<br>◯ Female

(8) What is [q_5_18]'s relation to you?

- ◯ Mother
- ◯ Father
- ◯ Brother
- ◯ Sister
- ◯ Spouse/partner/boyfriend/girlfriend
- ◯ Son
- ◯ Daughter
- ◯ Maternal Grandmother
- ◯ Maternal Grandfather
- ◯ Paternal Grandmother
- ◯ Paternal Grandfather
- ◯ Maternal Aunt
- ◯ Maternal Uncle
- ◯ Paternal Aunt
- ◯ Paternal Uncle
- ◯ Niece
- ◯ Nephew
- ◯ Friend
- ◯ Countrymen/Co-worker
- ◯ Mother-in-law
- ◯ Father-in-law
- ◯ Brother-in-law
- ◯ Sister-in-law
- ◯ Son-in-law
- ◯ Daughter-in-law
- ◯ Other

(8) Please specify: ______________________________

(8) What is [q_5_18]'s occupation?	◯ None/Unemployed (receive social grant)<br>◯ None/Unemployed (do not receive social grant)<br>◯ Farm work<br>◯ Domestic work<br>◯ Construction work<br>◯ Security work<br>◯ Cleaning work<br>◯ Small business owner<br>◯ Mine work<br>◯ Teacher<br>◯ Traditional healer<br>◯ Health sector (formal)<br>◯ Game farm/game reserve (e.g. ranger)<br>◯ Driver<br>◯ Skilled worker (e.g. plumber, mechanic, electrician)<br>◯ Cook/ chef/ catering<br>◯ Unskilled worker (e.g. general labourer)<br>◯ Artisan (e.g. carpenter, wood carver, weaver)<br>◯ Waiter/ barman<br>◯ Informal selling<br>◯ Small business assistant<br>◯ Clerical and office work<br>◯ Cattle herder<br>◯ Sewing, hairdressing, baking, brewing<br>◯ Police, soldier, fireman<br>◯ Petrol attendant<br>◯ Timber, sawmill, poles<br>◯ Gardening services<br>◯ Fieldworker - NGO or university<br>◯ Art, craft, photography, fashion design<br>◯ Senior administrator, manager, professional<br>◯ Priest/pastor<br>◯ Retail/clerk/sales<br>◯ Other<br>◯ Don't know
(8) Please specify:	__________________________
(8) How old is [q_5_18]?	__________________________
(9) Name of household member:	__________________________<br>(Include Name and Surname.)
(9) Is [q_5_19] the household head?	◯ Yes<br>◯ No
(9) Is [q_5_19] male or female?	◯ Male<br>◯ Female

(9) What is [q_5_19]'s relation to you?

- ◯ Mother
- ◯ Father
- ◯ Brother
- ◯ Sister
- ◯ Spouse/partner/boyfriend/girlfriend
- ◯ Son
- ◯ Daughter
- ◯ Maternal Grandmother
- ◯ Maternal Grandfather
- ◯ Paternal Grandmother
- ◯ Paternal Grandfather
- ◯ Maternal Aunt
- ◯ Maternal Uncle
- ◯ Paternal Aunt
- ◯ Paternal Uncle
- ◯ Niece
- ◯ Nephew
- ◯ Friend
- ◯ Countrymen/Co-worker
- ◯ Mother-in-law
- ◯ Father-in-law
- ◯ Brother-in-law
- ◯ Sister-in-law
- ◯ Son-in-law
- ◯ Daughter-in-law
- ◯ Other

(9) Please specify: ______________________________

(9) What is [q_5_19]'s occupation?	◯ None/Unemployed (receive social grant)<br>◯ None/Unemployed (do not receive social grant)<br>◯ Farm work<br>◯ Domestic work<br>◯ Construction work<br>◯ Security work<br>◯ Cleaning work<br>◯ Small business owner<br>◯ Mine work<br>◯ Teacher<br>◯ Traditional healer<br>◯ Health sector (formal)<br>◯ Game farm/game reserve (e.g. ranger)<br>◯ Driver<br>◯ Skilled worker (e.g. plumber, mechanic, electrician)<br>◯ Cook/ chef/ catering<br>◯ Unskilled worker (e.g. general labourer)<br>◯ Artisan (e.g. carpenter, wood carver, weaver)<br>◯ Waiter/ barman<br>◯ Informal selling<br>◯ Small business assistant<br>◯ Clerical and office work<br>◯ Cattle herder<br>◯ Sewing, hairdressing, baking, brewing<br>◯ Police, soldier, fireman<br>◯ Petrol attendant<br>◯ Timber, sawmill, poles<br>◯ Gardening services<br>◯ Fieldworker - NGO or university<br>◯ Art, craft, photography, fashion design<br>◯ Senior administrator, manager, professional<br>◯ Priest/pastor<br>◯ Retail/clerk/sales<br>◯ Other<br>◯ Don't know
(9) Please specify:	__________________________
(9) How old is [q_5_19]?	__________________________
(10) Name of household member:	__________________________<br>(Include Name and Surname.)
(10) Is [q_5_110] the household head?	◯ Yes<br>◯ No
(10) Is [q_5_110] male or female?	◯ Male<br>◯ Female

(10) What is [q_5_110]'s relation to you?

- ◯ Mother
- ◯ Father
- ◯ Brother
- ◯ Sister
- ◯ Spouse/partner/boyfriend/girlfriend
- ◯ Son
- ◯ Daughter
- ◯ Maternal Grandmother
- ◯ Maternal Grandfather
- ◯ Paternal Grandmother
- ◯ Paternal Grandfather
- ◯ Maternal Aunt
- ◯ Maternal Uncle
- ◯ Paternal Aunt
- ◯ Paternal Uncle
- ◯ Niece
- ◯ Nephew
- ◯ Friend
- ◯ Countrymen/Co-worker
- ◯ Mother-in-law
- ◯ Father-in-law
- ◯ Brother-in-law
- ◯ Sister-in-law
- ◯ Son-in-law
- ◯ Daughter-in-law
- ◯ Other

---

(10) Please specify: ______________________________

(10) What is [q_5_110]'s occupation?	◯ None/Unemployed (receive social grant)<br>◯ None/Unemployed (do not receive social grant)<br>◯ Farm work<br>◯ Domestic work<br>◯ Construction work<br>◯ Security work<br>◯ Cleaning work<br>◯ Small business owner<br>◯ Mine work<br>◯ Teacher<br>◯ Traditional healer<br>◯ Health sector (formal)<br>◯ Game farm/game reserve (e.g. ranger)<br>◯ Driver<br>◯ Skilled worker (e.g. plumber, mechanic, electrician)<br>◯ Cook/ chef/ catering<br>◯ Unskilled worker (e.g. general labourer)<br>◯ Artisan (e.g. carpenter, wood carver, weaver)<br>◯ Waiter/ barman<br>◯ Informal selling<br>◯ Small business assistant<br>◯ Clerical and office work<br>◯ Cattle herder<br>◯ Sewing, hairdressing, baking, brewing<br>◯ Police, soldier, fireman<br>◯ Petrol attendant<br>◯ Timber, sawmill, poles<br>◯ Gardening services<br>◯ Fieldworker - NGO or university<br>◯ Art, craft, photography, fashion design<br>◯ Senior administrator, manager, professional<br>◯ Priest/pastor<br>◯ Retail/clerk/sales<br>◯ Other<br>◯ Don't know
(10) Please specify:	__________________________
(10) How old is [q_5_110]?	__________________________
(11) Name of household member:	__________________________<br>(Include Name and Surname.)
(11) Is [q_5_111] the household head?	◯ Yes<br>◯ No
(11) Is [q_5_111] male or female?	◯ Male<br>◯ Female

(11) What is [q_5_111]'s relation to you?

- ◯ Mother
- ◯ Father
- ◯ Brother
- ◯ Sister
- ◯ Spouse/partner/boyfriend/girlfriend
- ◯ Son
- ◯ Daughter
- ◯ Maternal Grandmother
- ◯ Maternal Grandfather
- ◯ Paternal Grandmother
- ◯ Paternal Grandfather
- ◯ Maternal Aunt
- ◯ Maternal Uncle
- ◯ Paternal Aunt
- ◯ Paternal Uncle
- ◯ Niece
- ◯ Nephew
- ◯ Friend
- ◯ Countrymen/Co-worker
- ◯ Mother-in-law
- ◯ Father-in-law
- ◯ Brother-in-law
- ◯ Sister-in-law
- ◯ Son-in-law
- ◯ Daughter-in-law
- ◯ Other

(11) Please specify: ____________________

(11) What is [q_5_111]'s occupation?	◯ None/Unemployed (receive social grant)<br>◯ None/Unemployed (do not receive social grant)<br>◯ Farm work<br>◯ Domestic work<br>◯ Construction work<br>◯ Security work<br>◯ Cleaning work<br>◯ Small business owner<br>◯ Mine work<br>◯ Teacher<br>◯ Traditional healer<br>◯ Health sector (formal)<br>◯ Game farm/game reserve (e.g. ranger)<br>◯ Driver<br>◯ Skilled worker (e.g. plumber, mechanic, electrician)<br>◯ Cook/ chef/ catering<br>◯ Unskilled worker (e.g. general labourer)<br>◯ Artisan (e.g. carpenter, wood carver, weaver)<br>◯ Waiter/ barman<br>◯ Informal selling<br>◯ Small business assistant<br>◯ Clerical and office work<br>◯ Cattle herder<br>◯ Sewing, hairdressing, baking, brewing<br>◯ Police, soldier, fireman<br>◯ Petrol attendant<br>◯ Timber, sawmill, poles<br>◯ Gardening services<br>◯ Fieldworker - NGO or university<br>◯ Art, craft, photography, fashion design<br>◯ Senior administrator, manager, professional<br>◯ Priest/pastor<br>◯ Retail/clerk/sales<br>◯ Other<br>◯ Don't know
(11) Please specify:	__________________________
(11) How old is [q_5_111]?	__________________________
(12) Name of household member:	__________________________<br>(Include Name and Surname.)
(12) Is [q_5_112] the household head?	◯ Yes<br>◯ No
(12) Is [q_5_112] male or female?	◯ Male<br>◯ Female

(12) What is [q_5_112]'s relation to you?	◯ Mother<br>◯ Father<br>◯ Brother<br>◯ Sister<br>◯ Spouse/partner/boyfriend/girlfriend<br>◯ Son<br>◯ Daughter<br>◯ Maternal Grandmother<br>◯ Maternal Grandfather<br>◯ Paternal Grandmother<br>◯ Paternal Grandfather<br>◯ Maternal Aunt<br>◯ Maternal Uncle<br>◯ Paternal Aunt<br>◯ Paternal Uncle<br>◯ Niece<br>◯ Nephew<br>◯ Friend<br>◯ Countrymen/Co-worker<br>◯ Mother-in-law<br>◯ Father-in-law<br>◯ Brother-in-law<br>◯ Sister-in-law<br>◯ Son-in-law<br>◯ Daughter-in-law<br>◯ Other
(12) Please specify:	__________________________

(12) What is [q_5_112]'s occupation?

- ◯ None/Unemployed (receive social grant)
- ◯ None/Unemployed (do not receive social grant)
- ◯ Farm work
- ◯ Domestic work
- ◯ Construction work
- ◯ Security work
- ◯ Cleaning work
- ◯ Small business owner
- ◯ Mine work
- ◯ Teacher
- ◯ Traditional healer
- ◯ Health sector (formal)
- ◯ Game farm/game reserve (e.g. ranger)
- ◯ Driver
- ◯ Skilled worker (e.g. plumber, mechanic, electrician)
- ◯ Cook/ chef/ catering
- ◯ Unskilled worker (e.g. general labourer)
- ◯ Artisan (e.g. carpenter, wood carver, weaver)
- ◯ Waiter/ barman
- ◯ Informal selling
- ◯ Small business assistant
- ◯ Clerical and office work
- ◯ Cattle herder
- ◯ Sewing, hairdressing, baking, brewing
- ◯ Police, soldier, fireman
- ◯ Petrol attendant
- ◯ Timber, sawmill, poles
- ◯ Gardening services
- ◯ Fieldworker - NGO or university
- ◯ Art, craft, photography, fashion design
- ◯ Senior administrator, manager, professional
- ◯ Priest/pastor
- ◯ Retail/clerk/sales
- ◯ Other
- ◯ Don't know

---

(12) Please specify: __________________________________

---

(12) How old is [q_5_112]? __________________________________

When you've entered all [q_4_7] household members, select "Save and go to next instrument"

Residence History

____

RESIDENCE HISTORY

Have you lived anywhere besides the Agincourt study site since age 15 for a period of at least six months?

- ◯ Yes
- ◯ No
- ◯ Don't know

(Double check that this response is accurate.)

Now I want to ask you about your employment history. Not including your current work or employment status, how many other jobs have you held for wages or salary since the age of 15?

____

What was your main reason for moving to this place of residence?

- ◯ Job assignment/transfer
- ◯ Retrenchment
- ◯ Better employment opportunities
- ◯ Better accommodation prospects
- ◯ Better educational/training opportunities
- ◯ Closer to school or work
- ◯ Better public/social services
- ◯ Moved with family
- ◯ Joined family or friends
- ◯ Marriage
- ◯ Divorce
- ◯ Death of a family member
- ◯ Family responsibility
- ◯ Attraction to urban life style
- ◯ Evicted from previous residence
- ◯ Not able to pay rent
- ◯ Government resettlement
- ◯ Left to escape violence
- ◯ Other (specify)
- ◯ Dont know

Please specify

____

What was your main formal/informal occupation in the month BEFORE you moved?	◯ None<br>◯ Farm work<br>◯ Domestic work<br>◯ Construction work<br>◯ Security work<br>◯ Cleaning work<br>◯ Small business owner<br>◯ Mine work<br>◯ Teacher<br>◯ Traditional healer<br>◯ Health sector (formal)<br>◯ Game farm/game reserve (e.g. ranger)<br>◯ Driver<br>◯ Skilled worker (e.g. plumber, mechanic, electrician)<br>◯ Cook/ chef/ catering<br>◯ Unskilled worker (e.g. general labourer)<br>◯ Artisan (e.g. carpenter, wood carver, weaver)<br>◯ Waiter/ barman<br>◯ Informal selling<br>◯ Small business assistant<br>◯ Clerical and office work<br>◯ Cattle herder<br>◯ Sewing, hairdressing, baking, brewing<br>◯ Police, soldier, fireman<br>◯ Petrol attendant<br>◯ Timber, sawmill, poles<br>◯ Gardening services<br>◯ Fieldworker - NGO or university<br>◯ Art, craft, photography, fashion design<br>◯ Senior administrator, manager, professional<br>◯ Priest/pastor<br>◯ Retail/clerk/sales<br>◯ Other<br>◯ Don't know<br>(If unsure of how to classify, select "Other")
Please specify	__________________________________

What was your main formal/informal occupation in the month AFTER you moved?	◯ None<br>◯ Farm work<br>◯ Domestic work<br>◯ Construction work<br>◯ Security work<br>◯ Cleaning work<br>◯ Small business owner<br>◯ Mine work<br>◯ Teacher<br>◯ Traditional healer<br>◯ Health sector (formal)<br>◯ Game farm/game reserve (e.g. ranger)<br>◯ Driver<br>◯ Skilled worker (e.g. plumber, mechanic, electrician)<br>◯ Cook/ chef/ catering<br>◯ Unskilled worker (e.g. general labourer)<br>◯ Artisan (e.g. carpenter, wood carver, weaver)<br>◯ Waiter/ barman<br>◯ Informal selling<br>◯ Small business assistant<br>◯ Clerical and office work<br>◯ Cattle herder<br>◯ Sewing, hairdressing, baking, brewing<br>◯ Police, soldier, fireman<br>◯ Petrol attendant<br>◯ Timber, sawmill, poles<br>◯ Gardening services<br>◯ Fieldworker - NGO or university<br>◯ Art, craft, photography, fashion design<br>◯ Senior administrator, manager, professional<br>◯ Priest/pastor<br>◯ Retail/clerk/sales<br>◯ Other<br>◯ Don't know<br>(If unsure of how to classify, select "Other")
Please specify	__________________________________
Now I want to ask you about other places you have lived for a period of six months or more since age 15 years. Not including your current residence or [q_1_5] how many other places have you lived for a period of at least six months since you were age 15 years?	__________________________________<br>(Double check that this response is accurate.)

Mark as "Complete" and select "Save and go to next instrument" when complete.

Non Migrant Employment Loop

____

NON-MIGRANT EMPLOYMENT LOOP

Start with your most recent PREVIOUS job/work for pay/salary and repeat for each of the [q_6_2] job you've had since age 15.

(1) What was your main formal/informal occupation?

- ◯ None
- ◯ Farm work
- ◯ Domestic work
- ◯ Construction work
- ◯ Security work
- ◯ Cleaning work
- ◯ Small business owner
- ◯ Mine work
- ◯ Teacher
- ◯ Traditional healer
- ◯ Health sector (formal)
- ◯ Game farm/game reserve (e.g. ranger)
- ◯ Driver
- ◯ Skilled worker (e.g. plumber, mechanic, electrician)
- ◯ Cook/ chef/ catering
- ◯ Unskilled worker (e.g. general labourer)
- ◯ Artisan (e.g. carpenter, wood carver, weaver)
- ◯ Waiter/ barman
- ◯ Informal selling
- ◯ Small business assistant
- ◯ Clerical and office work
- ◯ Cattle herder
- ◯ Sewing, hairdressing, baking, brewing
- ◯ Police, soldier, fireman
- ◯ Petrol attendant
- ◯ Timber, sawmill, poles
- ◯ Gardening services
- ◯ Fieldworker - NGO or university
- ◯ Art, craft, photography, fashion design
- ◯ Senior administrator, manager, professional
- ◯ Priest/pastor
- ◯ Retail/clerk/sales
- ◯ Other
- ◯ Don't know

(If unsure of how to classify, select "Other")

(1) Please specify

____

(1) When did you START this job/work for pay/salary? MONTH	◯ January<br>◯ February<br>◯ March<br>◯ April<br>◯ May<br>◯ June<br>◯ July<br>◯ August<br>◯ September<br>◯ October<br>◯ November<br>◯ December<br>◯ Don't know
(1) When did you START this job/work for pay/salary? YEAR	◯ 2019<br>◯ 2018<br>◯ 2017<br>◯ 2016<br>◯ 2015<br>◯ 2014<br>◯ 2013<br>◯ 2012<br>◯ 2011<br>◯ 2010<br>◯ 2009<br>◯ 2008<br>◯ 2007<br>◯ 2006<br>◯ 2005<br>◯ 2004<br>◯ 2003<br>◯ 2002<br>◯ 2001<br>◯ 2000<br>◯ 1999<br>◯ 1998<br>◯ 1997<br>◯ 1996<br>◯ 1995<br>◯ 1994<br>◯ 1993<br>◯ 1992<br>◯ 1991<br>◯ 1990<br>◯ 1989<br>◯ 1988<br>◯ 1987<br>◯ 1986<br>◯ 1985<br>◯ 1984<br>◯ 1983<br>◯ 1982<br>◯ 1981<br>◯ 1980

(1) When did you END this job/work for pay/salary? MONTH	◯ January<br>◯ February<br>◯ March<br>◯ April<br>◯ May<br>◯ June<br>◯ July<br>◯ August<br>◯ September<br>◯ October<br>◯ November<br>◯ December<br>◯ Don't know
(1) When did you END this job/work for pay/salary? YEAR	◯ 2019<br>◯ 2018<br>◯ 2017<br>◯ 2016<br>◯ 2015<br>◯ 2014<br>◯ 2013<br>◯ 2012<br>◯ 2011<br>◯ 2010<br>◯ 2009<br>◯ 2008<br>◯ 2007<br>◯ 2006<br>◯ 2005<br>◯ 2004<br>◯ 2003<br>◯ 2002<br>◯ 2001<br>◯ 2000<br>◯ 1999<br>◯ 1998<br>◯ 1997<br>◯ 1996<br>◯ 1995<br>◯ 1994<br>◯ 1993<br>◯ 1992<br>◯ 1991<br>◯ 1990<br>◯ 1989<br>◯ 1988<br>◯ 1987<br>◯ 1986<br>◯ 1985<br>◯ 1984<br>◯ 1983<br>◯ 1982<br>◯ 1981<br>◯ 1980

(2) What was your main formal/informal occupation?	◯ None<br>◯ Farm work<br>◯ Domestic work<br>◯ Construction work<br>◯ Security work<br>◯ Cleaning work<br>◯ Small business owner<br>◯ Mine work<br>◯ Teacher<br>◯ Traditional healer<br>◯ Health sector (formal)<br>◯ Game farm/game reserve (e.g. ranger)<br>◯ Driver<br>◯ Skilled worker (e.g. plumber, mechanic, electrician)<br>◯ Cook/ chef/ catering<br>◯ Unskilled worker (e.g. general labourer)<br>◯ Artisan (e.g. carpenter, wood carver, weaver)<br>◯ Waiter/ barman<br>◯ Informal selling<br>◯ Small business assistant<br>◯ Clerical and office work<br>◯ Cattle herder<br>◯ Sewing, hairdressing, baking, brewing<br>◯ Police, soldier, fireman<br>◯ Petrol attendant<br>◯ Timber, sawmill, poles<br>◯ Gardening services<br>◯ Fieldworker - NGO or university<br>◯ Art, craft, photography, fashion design<br>◯ Senior administrator, manager, professional<br>◯ Priest/pastor<br>◯ Retail/clerk/sales<br>◯ Other<br>◯ Don't know<br>(If unsure of how to classify, select "Other")
(2) Please specify	______________________________
(2) When did you START this job/work for pay/salary? MONTH	◯ January<br>◯ February<br>◯ March<br>◯ April<br>◯ May<br>◯ June<br>◯ July<br>◯ August<br>◯ September<br>◯ October<br>◯ November<br>◯ December<br>◯ Don't know

(2) When did you START this job/work for pay/salary?
YEAR

- ◯ 2019
- ◯ 2018
- ◯ 2017
- ◯ 2016
- ◯ 2015
- ◯ 2014
- ◯ 2013
- ◯ 2012
- ◯ 2011
- ◯ 2010
- ◯ 2009
- ◯ 2008
- ◯ 2007
- ◯ 2006
- ◯ 2005
- ◯ 2004
- ◯ 2003
- ◯ 2002
- ◯ 2001
- ◯ 2000
- ◯ 1999
- ◯ 1998
- ◯ 1997
- ◯ 1996
- ◯ 1995
- ◯ 1994
- ◯ 1993
- ◯ 1992
- ◯ 1991
- ◯ 1990
- ◯ 1989
- ◯ 1988
- ◯ 1987
- ◯ 1986
- ◯ 1985
- ◯ 1984
- ◯ 1983
- ◯ 1982
- ◯ 1981
- ◯ 1980

(2) When did you END this job/work for pay/salary?
MONTH

- ◯ January
- ◯ February
- ◯ March
- ◯ April
- ◯ May
- ◯ June
- ◯ July
- ◯ August
- ◯ September
- ◯ October
- ◯ November
- ◯ December
- ◯ Don't know

(2) When did you END this job/work for pay/salary?
YEAR

- ◯ 2019
- ◯ 2018
- ◯ 2017
- ◯ 2016
- ◯ 2015
- ◯ 2014
- ◯ 2013
- ◯ 2012
- ◯ 2011
- ◯ 2010
- ◯ 2009
- ◯ 2008
- ◯ 2007
- ◯ 2006
- ◯ 2005
- ◯ 2004
- ◯ 2003
- ◯ 2002
- ◯ 2001
- ◯ 2000
- ◯ 1999
- ◯ 1998
- ◯ 1997
- ◯ 1996
- ◯ 1995
- ◯ 1994
- ◯ 1993
- ◯ 1992
- ◯ 1991
- ◯ 1990
- ◯ 1989
- ◯ 1988
- ◯ 1987
- ◯ 1986
- ◯ 1985
- ◯ 1984
- ◯ 1983
- ◯ 1982
- ◯ 1981
- ◯ 1980

(3) What was your main formal/informal occupation?	◯ None<br>◯ Farm work<br>◯ Domestic work<br>◯ Construction work<br>◯ Security work<br>◯ Cleaning work<br>◯ Small business owner<br>◯ Mine work<br>◯ Teacher<br>◯ Traditional healer<br>◯ Health sector (formal)<br>◯ Game farm/game reserve (e.g. ranger)<br>◯ Driver<br>◯ Skilled worker (e.g. plumber, mechanic, electrician)<br>◯ Cook/ chef/ catering<br>◯ Unskilled worker (e.g. general labourer)<br>◯ Artisan (e.g. carpenter, wood carver, weaver)<br>◯ Waiter/ barman<br>◯ Informal selling<br>◯ Small business assistant<br>◯ Clerical and office work<br>◯ Cattle herder<br>◯ Sewing, hairdressing, baking, brewing<br>◯ Police, soldier, fireman<br>◯ Petrol attendant<br>◯ Timber, sawmill, poles<br>◯ Gardening services<br>◯ Fieldworker - NGO or university<br>◯ Art, craft, photography, fashion design<br>◯ Senior administrator, manager, professional<br>◯ Priest/pastor<br>◯ Retail/clerk/sales<br>◯ Other<br>◯ Don't know<br>(If unsure of how to classify, select "Other")
(3) Please specify	__________________________________
(3) When did you START this job/work for pay/salary? MONTH	◯ January<br>◯ February<br>◯ March<br>◯ April<br>◯ May<br>◯ June<br>◯ July<br>◯ August<br>◯ September<br>◯ October<br>◯ November<br>◯ December<br>◯ Don't know

(3) When did you START this job/work for pay/salary?
YEAR

- ◯ 2019
- ◯ 2018
- ◯ 2017
- ◯ 2016
- ◯ 2015
- ◯ 2014
- ◯ 2013
- ◯ 2012
- ◯ 2011
- ◯ 2010
- ◯ 2009
- ◯ 2008
- ◯ 2007
- ◯ 2006
- ◯ 2005
- ◯ 2004
- ◯ 2003
- ◯ 2002
- ◯ 2001
- ◯ 2000
- ◯ 1999
- ◯ 1998
- ◯ 1997
- ◯ 1996
- ◯ 1995
- ◯ 1994
- ◯ 1993
- ◯ 1992
- ◯ 1991
- ◯ 1990
- ◯ 1989
- ◯ 1988
- ◯ 1987
- ◯ 1986
- ◯ 1985
- ◯ 1984
- ◯ 1983
- ◯ 1982
- ◯ 1981
- ◯ 1980

(3) When did you END this job/work for pay/salary?
MONTH

- ◯ January
- ◯ February
- ◯ March
- ◯ April
- ◯ May
- ◯ June
- ◯ July
- ◯ August
- ◯ September
- ◯ October
- ◯ November
- ◯ December
- ◯ Don't know

(3) When did you END this job/work for pay/salary?
YEAR

◯ 2019
◯ 2018
◯ 2017
◯ 2016
◯ 2015
◯ 2014
◯ 2013
◯ 2012
◯ 2011
◯ 2010
◯ 2009
◯ 2008
◯ 2007
◯ 2006
◯ 2005
◯ 2004
◯ 2003
◯ 2002
◯ 2001
◯ 2000
◯ 1999
◯ 1998
◯ 1997
◯ 1996
◯ 1995
◯ 1994
◯ 1993
◯ 1992
◯ 1991
◯ 1990
◯ 1989
◯ 1988
◯ 1987
◯ 1986
◯ 1985
◯ 1984
◯ 1983
◯ 1982
◯ 1981
◯ 1980

(4) What was your main formal/informal occupation?	◯ None<br>◯ Farm work<br>◯ Domestic work<br>◯ Construction work<br>◯ Security work<br>◯ Cleaning work<br>◯ Small business owner<br>◯ Mine work<br>◯ Teacher<br>◯ Traditional healer<br>◯ Health sector (formal)<br>◯ Game farm/game reserve (e.g. ranger)<br>◯ Driver<br>◯ Skilled worker (e.g. plumber, mechanic, electrician)<br>◯ Cook/ chef/ catering<br>◯ Unskilled worker (e.g. general labourer)<br>◯ Artisan (e.g. carpenter, wood carver, weaver)<br>◯ Waiter/ barman<br>◯ Informal selling<br>◯ Small business assistant<br>◯ Clerical and office work<br>◯ Cattle herder<br>◯ Sewing, hairdressing, baking, brewing<br>◯ Police, soldier, fireman<br>◯ Petrol attendant<br>◯ Timber, sawmill, poles<br>◯ Gardening services<br>◯ Fieldworker - NGO or university<br>◯ Art, craft, photography, fashion design<br>◯ Senior administrator, manager, professional<br>◯ Priest/pastor<br>◯ Retail/clerk/sales<br>◯ Other<br>◯ Don't know<br>(If unsure of how to classify, select "Other")
(4) Please specify	______________________________
(4) When did you START this job/work for pay/salary? MONTH	◯ January<br>◯ February<br>◯ March<br>◯ April<br>◯ May<br>◯ June<br>◯ July<br>◯ August<br>◯ September<br>◯ October<br>◯ November<br>◯ December<br>◯ Don't know

(4) When did you START this job/work for pay/salary?
YEAR

◯ 2019
◯ 2018
◯ 2017
◯ 2016
◯ 2015
◯ 2014
◯ 2013
◯ 2012
◯ 2011
◯ 2010
◯ 2009
◯ 2008
◯ 2007
◯ 2006
◯ 2005
◯ 2004
◯ 2003
◯ 2002
◯ 2001
◯ 2000
◯ 1999
◯ 1998
◯ 1997
◯ 1996
◯ 1995
◯ 1994
◯ 1993
◯ 1992
◯ 1991
◯ 1990
◯ 1989
◯ 1988
◯ 1987
◯ 1986
◯ 1985
◯ 1984
◯ 1983
◯ 1982
◯ 1981
◯ 1980

(4) When did you END this job/work for pay/salary?
MONTH

◯ January
◯ February
◯ March
◯ April
◯ May
◯ June
◯ July
◯ August
◯ September
◯ October
◯ November
◯ December
◯ Don't know

(4) When did you END this job/work for pay/salary? YEAR

◯ 2019
◯ 2018
◯ 2017
◯ 2016
◯ 2015
◯ 2014
◯ 2013
◯ 2012
◯ 2011
◯ 2010
◯ 2009
◯ 2008
◯ 2007
◯ 2006
◯ 2005
◯ 2004
◯ 2003
◯ 2002
◯ 2001
◯ 2000
◯ 1999
◯ 1998
◯ 1997
◯ 1996
◯ 1995
◯ 1994
◯ 1993
◯ 1992
◯ 1991
◯ 1990
◯ 1989
◯ 1988
◯ 1987
◯ 1986
◯ 1985
◯ 1984
◯ 1983
◯ 1982
◯ 1981
◯ 1980

(5) What was your main formal/informal occupation?	◯ None<br>◯ Farm work<br>◯ Domestic work<br>◯ Construction work<br>◯ Security work<br>◯ Cleaning work<br>◯ Small business owner<br>◯ Mine work<br>◯ Teacher<br>◯ Traditional healer<br>◯ Health sector (formal)<br>◯ Game farm/game reserve (e.g. ranger)<br>◯ Driver<br>◯ Skilled worker (e.g. plumber, mechanic, electrician)<br>◯ Cook/ chef/ catering<br>◯ Unskilled worker (e.g. general labourer)<br>◯ Artisan (e.g. carpenter, wood carver, weaver)<br>◯ Waiter/ barman<br>◯ Informal selling<br>◯ Small business assistant<br>◯ Clerical and office work<br>◯ Cattle herder<br>◯ Sewing, hairdressing, baking, brewing<br>◯ Police, soldier, fireman<br>◯ Petrol attendant<br>◯ Timber, sawmill, poles<br>◯ Gardening services<br>◯ Fieldworker - NGO or university<br>◯ Art, craft, photography, fashion design<br>◯ Senior administrator, manager, professional<br>◯ Priest/pastor<br>◯ Retail/clerk/sales<br>◯ Other<br>◯ Don't know<br>(If unsure of how to classify, select "Other")
(5) Please specify	__________________________________
(5) When did you START this job/work for pay/salary? MONTH	◯ January<br>◯ February<br>◯ March<br>◯ April<br>◯ May<br>◯ June<br>◯ July<br>◯ August<br>◯ September<br>◯ October<br>◯ November<br>◯ December<br>◯ Don't know

(5) When did you START this job/work for pay/salary?
YEAR

- 2019
- 2018
- 2017
- 2016
- 2015
- 2014
- 2013
- 2012
- 2011
- 2010
- 2009
- 2008
- 2007
- 2006
- 2005
- 2004
- 2003
- 2002
- 2001
- 2000
- 1999
- 1998
- 1997
- 1996
- 1995
- 1994
- 1993
- 1992
- 1991
- 1990
- 1989
- 1988
- 1987
- 1986
- 1985
- 1984
- 1983
- 1982
- 1981
- 1980

---

(5) When did you END this job/work for pay/salary?
MONTH

- January
- February
- March
- April
- May
- June
- July
- August
- September
- October
- November
- December
- Don't know

(5) When did you END this job/work for pay/salary?
YEAR

- ◯ 2019
- ◯ 2018
- ◯ 2017
- ◯ 2016
- ◯ 2015
- ◯ 2014
- ◯ 2013
- ◯ 2012
- ◯ 2011
- ◯ 2010
- ◯ 2009
- ◯ 2008
- ◯ 2007
- ◯ 2006
- ◯ 2005
- ◯ 2004
- ◯ 2003
- ◯ 2002
- ◯ 2001
- ◯ 2000
- ◯ 1999
- ◯ 1998
- ◯ 1997
- ◯ 1996
- ◯ 1995
- ◯ 1994
- ◯ 1993
- ◯ 1992
- ◯ 1991
- ◯ 1990
- ◯ 1989
- ◯ 1988
- ◯ 1987
- ◯ 1986
- ◯ 1985
- ◯ 1984
- ◯ 1983
- ◯ 1982
- ◯ 1981
- ◯ 1980

When you've completed [q_6_2] loops, select "Save and go to next instrument"

Residence History Loop

____

RESIDENCE HISTORY LOOP

Not including your current residence or your origin location/home village, begin with your previous place of residence and repeat for each of the [q_6_10] move(s) you made since you were 15

What province?

- ◯ Mpumalanga
- ◯ Gauteng
- ◯ Limpopo
- ◯ Free State
- ◯ KwaZulu Natal
- ◯ Eastern Cape
- ◯ Western Cape
- ◯ Northern Cape
- ◯ North West
- ◯ Other

Please specify: ______________________________

Where within Mpumalanga?

- ◯ Agincourt study site
- ◯ Barberton
- ◯ Bushbuckridge
- ◯ Dwarsloop
- ◯ Game park Mpumalanga
- ◯ Gemu / Hazyview
- ◯ Graskop/ Pilgrim's rest
- ◯ Kangwane (former homeland)
- ◯ Kiepersol
- ◯ Komatipoort
- ◯ Machadodorp
- ◯ Malelane
- ◯ Mapulaneng / Bushbuckridge
- ◯ Marite / Alexandria
- ◯ Matsulu
- ◯ Middleburg
- ◯ Mkhuhlu
- ◯ Nelspruit
- ◯ Pienaar (Nelspruit)
- ◯ Sabie
- ◯ Thulamahashe
- ◯ White River
- ◯ Witbank
- ◯ Zwelitsha
- ◯ Other Mpumalanga
- ◯ Don't know

Please specify: ______________________________

Where within Gauteng

- ◯ Akasia
- ◯ Alberton
- ◯ Alexandra
- ◯ Atteridgeville
- ◯ Babelegi
- ◯ Bapsfontein
- ◯ Bashewa
- ◯ Baviaanspoort
- ◯ Bekkersdal
- ◯ Benoni
- ◯ Bhongweni
- ◯ Blue Hills
- ◯ Boipatong
- ◯ Boksburg
- ◯ Bon Accord
- ◯ Bonamanzi Marina and Country Club
- ◯ Bophelong
- ◯ Boschkop
- ◯ Bosplaas Mathabe
- ◯ Bothasgeluk
- ◯ Brakpan
- ◯ Breswol
- ◯ Bronkhorstspruit
- ◯ Bultfontein
- ◯ Carletonville
- ◯ Centurion
- ◯ Chartwell
- ◯ Cheetah Park
- ◯ Chief A Luthuli Park
- ◯ City of Johannesburg NU
- ◯ Clayville
- ◯ Cullinan
- ◯ Dainfern
- ◯ Daveyton
- ◯ Deelkraal Gold Mine
- ◯ Derdepoort
- ◯ Devon A
- ◯ Devon B
- ◯ Diepsloot
- ◯ Dilopye
- ◯ Donkerhoek
- ◯ Doornfontein
- ◯ Doornkraal
- ◯ Downbern
- ◯ Drie Ziek
- ◯ Duduza
- ◯ Dukathole
- ◯ East Driefontein
- ◯ East Village
- ◯ Ebony Park
- ◯ Edenvale
- ◯ Eersterust
- ◯ Ekangala
- ◯ Ekurhuleni NU
- ◯ Elandsfontein
- ◯ Elandsrand
- ◯ Elandsridge
- ◯ Elsburg Gold Mine
- ◯ Emfuleni NU
- ◯ Endicott
- ◯ Ennerdale
- ◯ Etwatwa
- ◯ Evaton
- ◯ Farmall
- ◯ Fochville
- ◯ Ga-Rankuwa
- ◯ Geluksdal
- ◯ Germiston
- ◯ Glen Harvie

◯ Golden Gardens
◯ Goudvlakte West
◯ Green Park
◯ Grootfontein
◯ Grootvlei
◯ Haakdoornboom
◯ Hallgate
◯ Hammanskraal
◯ Harry Gwala
◯ Hartebeesfontein
◯ Hebron
◯ Heidelberg
◯ Hillshaven
◯ Holfontein
◯ Impumelelo
◯ Itsoseng
◯ Ivory Park
◯ Johandeo
◯ Johannesburg
◯ Kaalfontein
◯ Kagiso
◯ Kameeldrift
◯ Kameelfontein
◯ Kameelkraal
◯ Kanana
◯ Kanana Park
◯ Katlehong
◯ Other
◯ Keinfontein
◯ Kekana Garden
◯ Kempton Park
◯ Khutsong
◯ Kleinfontein
◯ Kokosi
◯ Krugersdorp
◯ Kungwini Part 2
◯ Kwa-Thema
◯ Lakeside
◯ Langaville
◯ Lanseria
◯ Laudium
◯ Lawley
◯ Leeufontein
◯ Leeuport
◯ Lehae
◯ Lenasia
◯ Lenasia South
◯ Lenz
◯ Lesedi NU
◯ Letsatsing
◯ Lindelani Village
◯ Lucky 7
◯ Mabopane
◯ Magaliesburg
◯ Majaneng
◯ Makanyaneng
◯ Malatjie
◯ Mamello
◯ Mamelodi
◯ Mandela Village
◯ Marokolong
◯ Mashemong
◯ Mayibuye
◯ Merafong City NU
◯ Meyerton
◯ Midrand
◯ Midvaal NU
◯ Millgate Farm
◯ Mogale City NU
◯ Mohlakeng
◯ Mooiplaas

◯ Munsieville
◯ Nellmapius
◯ New Eersterus
◯ Nigel
◯ Nooitgecht
◯ Nufcor
◯ Oberholzer
◯ Olievenhoutbos
◯ Onverwacht
◯ Orange Farm
◯ Orient Hills
◯ Pebble Rock Golf Village
◯ Phomolong
◯ Poortjie
◯ Pretoria
◯ Rabie Ridge
◯ Ramotse
◯ Randburg
◯ Randfontein
◯ Randfontein NU
◯ Randvaal
◯ Ratanda
◯ Rayton
◯ Refilwe
◯ Rethabiseng
◯ Rietfontein
◯ Rietvallei
◯ Roodeplaat
◯ Roodepoort
◯ Sable Hills Waterfront Estate
◯ Sandton
◯ Saulsville
◯ Seberuberung
◯ Sebokeng
◯ Sharpeville
◯ Simunye
◯ Sonstraal
◯ Soshanguve
◯ Southdene
◯ Soutpan
◯ Soweto
◯ Spaarwater
◯ Springs
◯ Stinkwater
◯ Stretford
◯ Suurman
◯ Temba
◯ Tembisa
◯ The Carousel Casino and Entertainment World
◯ The Hill
◯ The Village
◯ Thembisile
◯ Thinasonke
◯ Tierpoort
◯ Tokoza
◯ Tsakane
◯ Tsebe
◯ Tshepiso
◯ Tshepisong
◯ Tshwane NU
◯ Tweedracht
◯ Tweefontein
◯ Vaal Marina
◯ Vaal Oewer
◯ Vaalbank
◯ Vanderbijlpark
◯ Vastfontein
◯ Venterspost
◯ Vereeniging
◯ Viskuil
◯ Vlakfontein

◯ Vosloorus
◯ Wageenbietjieskop
◯ Walkerville
◯ Wallis Haven
◯ Wallmannsthal
◯ Waterpan
◯ Waterval
◯ Waterworks
◯ Wattville
◯ Wedela
◯ Welverdiend
◯ West Village
◯ West-Driefontein
◯ Western Areas Gold Mine
◯ Western Deep Levels Mine
◯ Westonaria
◯ Westonaria NU
◯ Winterveld
◯ Wolwekrans
◯ Zakariyya Park
◯ Zenzele
◯ Zevenfontein
◯ Zithobeni
◯ Zwartkop
◯ Zwavelpoort

Please specify:

____

Where within Limpopo?

◯ Aganang
◯ Ba-Phalaborwa
◯ Bela-Bela
◯ Blouberg
◯ Elias Motsoaledi
◯ Fetakgomo
◯ Greater Giyani
◯ Greater Letaba
◯ Greater Marble Hall
◯ Greater Tubatse
◯ Greater Tzaneen
◯ Lepelle-Nkumpi
◯ Lephalale
◯ Makhado
◯ Makhuduthamaga
◯ Malamulele
◯ Maruleng
◯ Modimolle
◯ Mogalakwena
◯ Molemole
◯ Mookgopong
◯ Musina
◯ Polokwane
◯ Thabazimbi
◯ Thulamela
◯ Other
◯ Don't know

Please specify:

____

Where within Western Cape?

____

Where within Eastern Cape? __________________________________

Where within North West? __________________________________

Where within KwaZulu Natal? __________________________________

Where within Northern Cape? __________________________________

Where within Free State? __________________________________

(1) Was it rural or urban?

- ◯ Rural
- ◯ Urban (city or town)
- ◯ Don't know

(Rural meaning locations like villages, agricultural areas, or game farms)

(1) When did you start living there? MONTH

- ◯ January
- ◯ February
- ◯ March
- ◯ April
- ◯ May
- ◯ June
- ◯ July
- ◯ August
- ◯ September
- ◯ October
- ◯ November
- ◯ December
- ◯ Don't know

(1) When did you start living there? YEAR

◯ 2019
◯ 2018
◯ 2017
◯ 2016
◯ 2015
◯ 2014
◯ 2013
◯ 2012
◯ 2011
◯ 2010
◯ 2009
◯ 2008
◯ 2007
◯ 2006
◯ 2005
◯ 2004
◯ 2003
◯ 2002
◯ 2001
◯ 2000
◯ 1999
◯ 1998
◯ 1997
◯ 1996
◯ 1995
◯ 1994
◯ 1993
◯ 1992
◯ 1991
◯ 1990
◯ 1989
◯ 1988
◯ 1987
◯ 1986
◯ 1985
◯ 1984
◯ 1983
◯ 1982
◯ 1981
◯ 1980
◯ Don't Know

(1) What was your main reason for moving to this place of residence?	◯ Job assignment/transfer<br>◯ Retrenchment<br>◯ Better employment opportunities<br>◯ Better accommodation prospects<br>◯ Better educational/training opportunities<br>◯ Closer to school or work<br>◯ Better public/social services<br>◯ Moved with family<br>◯ Joined family or friends<br>◯ Marriage<br>◯ Divorce<br>◯ Death of a family member<br>◯ Family responsibility<br>◯ Attraction to urban life style<br>◯ Evicted from previous residence<br>◯ Not able to pay rent<br>◯ Government resettlement<br>◯ Left to escape violence<br>◯ Other (specify)<br>◯ Dont know
(1) Please specify	__________________________________

(1) What was your main formal/informal occupation in the month BEFORE you moved?	◯ None<br>◯ Farm work<br>◯ Domestic work<br>◯ Construction work<br>◯ Security work<br>◯ Cleaning work<br>◯ Small business owner<br>◯ Mine work<br>◯ Teacher<br>◯ Traditional healer<br>◯ Health sector (formal)<br>◯ Game farm/game reserve (e.g. ranger)<br>◯ Driver<br>◯ Skilled worker (e.g. plumber, mechanic, electrician)<br>◯ Cook/ chef/ catering<br>◯ Unskilled worker (e.g. general labourer)<br>◯ Artisan (e.g. carpenter, wood carver, weaver)<br>◯ Waiter/ barman<br>◯ Informal selling<br>◯ Small business assistant<br>◯ Clerical and office work<br>◯ Cattle herder<br>◯ Sewing, hairdressing, baking, brewing<br>◯ Police, soldier, fireman<br>◯ Petrol attendant<br>◯ Timber, sawmill, poles<br>◯ Gardening services<br>◯ Fieldworker - NGO or university<br>◯ Art, craft, photography, fashion design<br>◯ Senior administrator, manager, professional<br>◯ Priest/pastor<br>◯ Retail/clerk/sales<br>◯ Student<br>◯ Other<br>◯ Don't know<br>(If unsure of how to classify, select "Other")
(1) Please specify	__________________________________

(1) What was your main formal/informal occupation in the month AFTER you moved?	◯ None<br>◯ Farm work<br>◯ Domestic work<br>◯ Construction work<br>◯ Security work<br>◯ Cleaning work<br>◯ Small business owner<br>◯ Mine work<br>◯ Teacher<br>◯ Traditional healer<br>◯ Health sector (formal)<br>◯ Game farm/game reserve (e.g. ranger)<br>◯ Driver<br>◯ Skilled worker (e.g. plumber, mechanic, electrician)<br>◯ Cook/ chef/ catering<br>◯ Unskilled worker (e.g. general labourer)<br>◯ Artisan (e.g. carpenter, wood carver, weaver)<br>◯ Waiter/ barman<br>◯ Informal selling<br>◯ Small business assistant<br>◯ Clerical and office work<br>◯ Cattle herder<br>◯ Sewing, hairdressing, baking, brewing<br>◯ Police, soldier, fireman<br>◯ Petrol attendant<br>◯ Timber, sawmill, poles<br>◯ Gardening services<br>◯ Fieldworker - NGO or university<br>◯ Art, craft, photography, fashion design<br>◯ Senior administrator, manager, professional<br>◯ Priest/pastor<br>◯ Retail/clerk/sales<br>◯ Student<br>◯ Other<br>◯ Don't know<br>(If unsure of how to classify, select "Other")
(1) Please specify	__________________________________
(1) In what type of place did you live?	◯ Commercial farm<br>◯ Game farm/nature reserve<br>◯ Mine<br>◯ Military base<br>◯ City (inner)<br>◯ City (suburb)<br>◯ Town<br>◯ Village (trust land)<br>◯ Informal settlement (city)<br>◯ Informal settlement (rural)<br>◯ Township (urban)<br>◯ Township (rural)<br>◯ Other<br>◯ Don't know
(1) Please specify	__________________________________

(1) What type of dwelling did you live in?	◯ House, brick or concrete structure on a separate stand<br>◯ Traditional dwelling, hut or structure made of traditional materials<br>◯ Flat or apartment in a block of flats/complex<br>◯ Cluster house in a complex<br>◯ Townhouse<br>◯ Semi-detached house not in a complex<br>◯ House, flat or room separate from main dwelling in backyard<br>◯ Informal dwelling or shack in backyard<br>◯ Informal dwelling NOT in backyard, e.g. in informal squatter settlement or on a farm<br>◯ Room or flat which is part of main dwelling or property<br>◯ Caravan or tent<br>◯ Unit in a retirement home or barracks etc.<br>◯ Other<br>◯ Don't know
(1) Please specify	__________________________________
(1) With?whom did you live at this residence?	☐ Parent(s)<br>☐ Spouse/partner/boyfriend/girlfriend<br>☐ Spouse and children<br>☐ Parent(s) in-law<br>☐ Friends<br>☐ Relatives<br>☐ Countrymen<br>☐ Alone<br>☐ Second family<br>☐ Other<br>☐ Don't know
(1) Please specify	__________________________________
(1) When did you stop living there? MONTH	◯ January<br>◯ February<br>◯ March<br>◯ April<br>◯ May<br>◯ June<br>◯ July<br>◯ August<br>◯ September<br>◯ October<br>◯ November<br>◯ December<br>◯ Don't know

(1) When did you stop living there? YEAR	◯ 2019<br>◯ 2018<br>◯ 2017<br>◯ 2016<br>◯ 2015<br>◯ 2014<br>◯ 2013<br>◯ 2012<br>◯ 2011<br>◯ 2010<br>◯ 2009<br>◯ 2008<br>◯ 2007<br>◯ 2006<br>◯ 2005<br>◯ 2004<br>◯ 2003<br>◯ 2002<br>◯ 2001<br>◯ 2000<br>◯ 1999<br>◯ 1998<br>◯ 1997<br>◯ 1996<br>◯ 1995<br>◯ 1994<br>◯ 1993<br>◯ 1992<br>◯ 1991<br>◯ 1990<br>◯ 1989<br>◯ 1988<br>◯ 1987<br>◯ 1986<br>◯ 1985<br>◯ 1984<br>◯ 1983<br>◯ 1982<br>◯ 1981<br>◯ 1980<br>◯ Don't Know
The following questions are for the place you lived before that	
What province?	◯ Mpumalanga<br>◯ Gauteng<br>◯ Limpopo<br>◯ Free State<br>◯ KwaZulu Natal<br>◯ Eastern Cape<br>◯ Western Cape<br>◯ Northern Cape<br>◯ North West<br>◯ Other
Please specify:	__________________________________

Where within Mpumalanga?	◯ Agincourt study site<br>◯ Barberton<br>◯ Bushbuckridge<br>◯ Dwarsloop<br>◯ Game park Mpumalanga<br>◯ Gemu / Hazyview<br>◯ Graskop/ Pilgrim's rest<br>◯ Kangwane (former homeland)<br>◯ Kiepersol<br>◯ Komatipoort<br>◯ Machadodorp<br>◯ Malelane<br>◯ Mapulaneng / Bushbuckridge<br>◯ Marite / Alexandria<br>◯ Matsulu<br>◯ Middleburg<br>◯ Mkhuhlu<br>◯ Nelspruit<br>◯ Pienaar (Nelspruit)<br>◯ Sabie<br>◯ Thulamahashe<br>◯ White River<br>◯ Witbank<br>◯ Zwelitsha<br>◯ Other Mpumalanga<br>◯ Don't know
Please specify:	__________________________________

Where within Gauteng

- ◯ Akasia
- ◯ Alberton
- ◯ Alexandra
- ◯ Atteridgeville
- ◯ Babelegi
- ◯ Bapsfontein
- ◯ Bashewa
- ◯ Baviaanspoort
- ◯ Bekkersdal
- ◯ Benoni
- ◯ Bhongweni
- ◯ Blue Hills
- ◯ Boipatong
- ◯ Boksburg
- ◯ Bon Accord
- ◯ Bonamanzi Marina and Country Club
- ◯ Bophelong
- ◯ Boschkop
- ◯ Bosplaas Mathabe
- ◯ Bothasgeluk
- ◯ Brakpan
- ◯ Breswol
- ◯ Bronkhorstspruit
- ◯ Bultfontein
- ◯ Carletonville
- ◯ Centurion
- ◯ Chartwell
- ◯ Cheetah Park
- ◯ Chief A Luthuli Park
- ◯ City of Johannesburg NU
- ◯ Clayville
- ◯ Cullinan
- ◯ Dainfern
- ◯ Daveyton
- ◯ Deelkraal Gold Mine
- ◯ Derdepoort
- ◯ Devon A
- ◯ Devon B
- ◯ Diepsloot
- ◯ Dilopye
- ◯ Donkerhoek
- ◯ Doornfontein
- ◯ Doornkraal
- ◯ Downbern
- ◯ Drie Ziek
- ◯ Duduza
- ◯ Dukathole
- ◯ East Driefontein
- ◯ East Village
- ◯ Ebony Park
- ◯ Edenvale
- ◯ Eersterust
- ◯ Ekangala
- ◯ Ekurhuleni NU
- ◯ Elandsfontein
- ◯ Elandsrand
- ◯ Elandsridge
- ◯ Elsburg Gold Mine
- ◯ Emfuleni NU
- ◯ Endicott
- ◯ Ennerdale
- ◯ Etwatwa
- ◯ Evaton
- ◯ Farmall
- ◯ Fochville
- ◯ Ga-Rankuwa
- ◯ Geluksdal
- ◯ Germiston
- ◯ Glen Harvie

◯ Golden Gardens
◯ Goudvlakte West
◯ Green Park
◯ Grootfontein
◯ Grootvlei
◯ Haakdoornboom
◯ Hallgate
◯ Hammanskraal
◯ Harry Gwala
◯ Hartebeesfontein
◯ Hebron
◯ Heidelberg
◯ Hillshaven
◯ Holfontein
◯ Impumelelo
◯ Itsoseng
◯ Ivory Park
◯ Johandeo
◯ Johannesburg
◯ Kaalfontein
◯ Kagiso
◯ Kameeldrift
◯ Kameelfontein
◯ Kameelkraal
◯ Kanana
◯ Kanana Park
◯ Katlehong
◯ Other
◯ Keinfontein
◯ Kekana Garden
◯ Kempton Park
◯ Khutsong
◯ Kleinfontein
◯ Kokosi
◯ Krugersdorp
◯ Kungwini Part 2
◯ Kwa-Thema
◯ Lakeside
◯ Langaville
◯ Lanseria
◯ Laudium
◯ Lawley
◯ Leeufontein
◯ Leeuport
◯ Lehae
◯ Lenasia
◯ Lenasia South
◯ Lenz
◯ Lesedi NU
◯ Letsatsing
◯ Lindelani Village
◯ Lucky 7
◯ Mabopane
◯ Magaliesburg
◯ Majaneng
◯ Makanyaneng
◯ Malatjie
◯ Mamello
◯ Mamelodi
◯ Mandela Village
◯ Marokolong
◯ Mashemong
◯ Mayibuye
◯ Merafong City NU
◯ Meyerton
◯ Midrand
◯ Midvaal NU
◯ Millgate Farm
◯ Mogale City NU
◯ Mohlakeng
◯ Mooiplaas

◯ Munsieville
◯ Nellmapius
◯ New Eersterus
◯ Nigel
◯ Nooitgecht
◯ Nufcor
◯ Oberholzer
◯ Olievenhoutbos
◯ Onverwacht
◯ Orange Farm
◯ Orient Hills
◯ Pebble Rock Golf Village
◯ Phomolong
◯ Poortjie
◯ Pretoria
◯ Rabie Ridge
◯ Ramotse
◯ Randburg
◯ Randfontein
◯ Randfontein NU
◯ Randvaal
◯ Ratanda
◯ Rayton
◯ Refilwe
◯ Rethabiseng
◯ Rietfontein
◯ Rietvallei
◯ Roodeplaat
◯ Roodepoort
◯ Sable Hills Waterfront Estate
◯ Sandton
◯ Saulsville
◯ Seberuberung
◯ Sebokeng
◯ Sharpeville
◯ Simunye
◯ Sonstraal
◯ Soshanguve
◯ Southdene
◯ Soutpan
◯ Soweto
◯ Spaarwater
◯ Springs
◯ Stinkwater
◯ Stretford
◯ Suurman
◯ Temba
◯ Tembisa
◯ The Carousel Casino and Entertainment World
◯ The Hill
◯ The Village
◯ Thembisile
◯ Thinasonke
◯ Tierpoort
◯ Tokoza
◯ Tsakane
◯ Tsebe
◯ Tshepiso
◯ Tshepisong
◯ Tshwane NU
◯ Tweedracht
◯ Tweefontein
◯ Vaal Marina
◯ Vaal Oewer
◯ Vaalbank
◯ Vanderbijlpark
◯ Vastfontein
◯ Venterspost
◯ Vereeniging
◯ Viskuil
◯ Vlakfontein

◯ Vosloorus
◯ Wageenbietjieskop
◯ Walkerville
◯ Wallis Haven
◯ Wallmannsthal
◯ Waterpan
◯ Waterval
◯ Waterworks
◯ Wattville
◯ Wedela
◯ Welverdiend
◯ West Village
◯ West-Driefontein
◯ Western Areas Gold Mine
◯ Western Deep Levels Mine
◯ Westonaria
◯ Westonaria NU
◯ Winterveld
◯ Wolwekrans
◯ Zakariyya Park
◯ Zenzele
◯ Zevenfontein
◯ Zithobeni
◯ Zwartkop
◯ Zwavelpoort

Please specify:

____

Where within Limpopo?

◯ Aganang
◯ Ba-Phalaborwa
◯ Bela-Bela
◯ Blouberg
◯ Elias Motsoaledi
◯ Fetakgomo
◯ Greater Giyani
◯ Greater Letaba
◯ Greater Marble Hall
◯ Greater Tubatse
◯ Greater Tzaneen
◯ Lepelle-Nkumpi
◯ Lephalale
◯ Makhado
◯ Makhuduthamaga
◯ Malamulele
◯ Maruleng
◯ Modimolle
◯ Mogalakwena
◯ Molemole
◯ Mookgopong
◯ Musina
◯ Polokwane
◯ Thabazimbi
◯ Thulamela
◯ Other
◯ Don't know

Please specify:

____

Where within Western Cape?

____

Where within Eastern Cape?

____

Where within North West?

____

Where within KwaZulu Natal?

____

Where within Northern Cape?

____

Where within Free State?

____

(2) Was it rural or urban?

◯ Rural
◯ Urban (city or town)
◯ Don't know
(Rural meaning locations like villages, agricultural areas, or game farms)

(2) When did you start living there? MONTH

◯ January
◯ February
◯ March
◯ April
◯ May
◯ June
◯ July
◯ August
◯ September
◯ October
◯ November
◯ December
◯ Don't know

(2) When did you start living there? YEAR

◯ 2019
◯ 2018
◯ 2017
◯ 2016
◯ 2015
◯ 2014
◯ 2013
◯ 2012
◯ 2011
◯ 2010
◯ 2009
◯ 2008
◯ 2007
◯ 2006
◯ 2005
◯ 2004
◯ 2003
◯ 2002
◯ 2001
◯ 2000
◯ 1999
◯ 1998
◯ 1997
◯ 1996
◯ 1995
◯ 1994
◯ 1993
◯ 1992
◯ 1991
◯ 1990
◯ 1989
◯ 1988
◯ 1987
◯ 1986
◯ 1985
◯ 1984
◯ 1983
◯ 1982
◯ 1981
◯ 1980
◯ Don't Know

(2) What was your main reason for moving to this place of residence?	◯ Job assignment/transfer<br>◯ Retrenchment<br>◯ Better employment opportunities<br>◯ Better accommodation prospects<br>◯ Better educational/training opportunities<br>◯ Closer to school or work<br>◯ Better public/social services<br>◯ Moved with family<br>◯ Joined family or friends<br>◯ Marriage<br>◯ Divorce<br>◯ Death of a family member<br>◯ Family responsibility<br>◯ Attraction to urban life style<br>◯ Evicted from previous residence<br>◯ Not able to pay rent<br>◯ Government resettlement<br>◯ Left to escape violence<br>◯ Other (specify)<br>◯ Dont know
(2) Please specify	__________________________________

(2) What was your main formal/informal occupation in the month BEFORE you moved?

- ◯ None
- ◯ Farm work
- ◯ Domestic work
- ◯ Construction work
- ◯ Security work
- ◯ Cleaning work
- ◯ Small business owner
- ◯ Mine work
- ◯ Teacher
- ◯ Traditional healer
- ◯ Health sector (formal)
- ◯ Game farm/game reserve (e.g. ranger)
- ◯ Driver
- ◯ Skilled worker (e.g. plumber, mechanic, electrician)
- ◯ Cook/ chef/ catering
- ◯ Unskilled worker (e.g. general labourer)
- ◯ Artisan (e.g. carpenter, wood carver, weaver)
- ◯ Waiter/ barman
- ◯ Informal selling
- ◯ Small business assistant
- ◯ Clerical and office work
- ◯ Cattle herder
- ◯ Sewing, hairdressing, baking, brewing
- ◯ Police, soldier, fireman
- ◯ Petrol attendant
- ◯ Timber, sawmill, poles
- ◯ Gardening services
- ◯ Fieldworker - NGO or university
- ◯ Art, craft, photography, fashion design
- ◯ Senior administrator, manager, professional
- ◯ Priest/pastor
- ◯ Retail/clerk/sales
- ◯ Student
- ◯ Other
- ◯ Don't know

(If unsure of how to classify, select "Other")

(2) Please specify

____

(2) What was your main formal/informal occupation in the month AFTER you moved?	◯ None<br>◯ Farm work<br>◯ Domestic work<br>◯ Construction work<br>◯ Security work<br>◯ Cleaning work<br>◯ Small business owner<br>◯ Mine work<br>◯ Teacher<br>◯ Traditional healer<br>◯ Health sector (formal)<br>◯ Game farm/game reserve (e.g. ranger)<br>◯ Driver<br>◯ Skilled worker (e.g. plumber, mechanic, electrician)<br>◯ Cook/ chef/ catering<br>◯ Unskilled worker (e.g. general labourer)<br>◯ Artisan (e.g. carpenter, wood carver, weaver)<br>◯ Waiter/ barman<br>◯ Informal selling<br>◯ Small business assistant<br>◯ Clerical and office work<br>◯ Cattle herder<br>◯ Sewing, hairdressing, baking, brewing<br>◯ Police, soldier, fireman<br>◯ Petrol attendant<br>◯ Timber, sawmill, poles<br>◯ Gardening services<br>◯ Fieldworker - NGO or university<br>◯ Art, craft, photography, fashion design<br>◯ Senior administrator, manager, professional<br>◯ Priest/pastor<br>◯ Retail/clerk/sales<br>◯ Student<br>◯ Other<br>◯ Don't know<br>(If unsure of how to classify, select "Other")
(2) Please specify	__________________________________
(2) In what type of place did you live?	◯ Commercial farm<br>◯ Game farm/nature reserve<br>◯ Mine<br>◯ Military base<br>◯ City (inner)<br>◯ City (suburb)<br>◯ Town<br>◯ Village (trust land)<br>◯ Informal settlement (city)<br>◯ Informal settlement (rural)<br>◯ Township (urban)<br>◯ Township (rural)<br>◯ Other<br>◯ Don't know
(2) Please specify	__________________________________

(2) What type of dwelling did you live in?

- ◯ House, brick or concrete structure on a separate stand
- ◯ Traditional dwelling, hut or structure made of traditional materials
- ◯ Flat or apartment in a block of flats/complex
- ◯ Cluster house in a complex
- ◯ Townhouse
- ◯ Semi-detached house not in a complex
- ◯ House, flat or room separate from main dwelling in backyard
- ◯ Informal dwelling or shack in backyard
- ◯ Informal dwelling NOT in backyard, e.g. in informal squatter settlement or on a farm
- ◯ Room or flat which is part of main dwelling or property
- ◯ Caravan or tent
- ◯ Unit in a retirement home or barracks etc.
- ◯ Other
- ◯ Don't know

(2) Please specify

____

(2) With?whom did you live at this residence?

- ☐ Parent(s)
- ☐ Spouse/partner/boyfriend/girlfriend
- ☐ Spouse and children
- ☐ Parent(s) in-law
- ☐ Friends
- ☐ Relatives
- ☐ Countrymen
- ☐ Alone
- ☐ Second family
- ☐ Other
- ☐ Don't know

(2) Please specify

____

(2) When did you stop living there? MONTH

- ◯ January
- ◯ February
- ◯ March
- ◯ April
- ◯ May
- ◯ June
- ◯ July
- ◯ August
- ◯ September
- ◯ October
- ◯ November
- ◯ December
- ◯ Don't know

(2) When did you stop living there? YEAR

- ◯ 2019
- ◯ 2018
- ◯ 2017
- ◯ 2016
- ◯ 2015
- ◯ 2014
- ◯ 2013
- ◯ 2012
- ◯ 2011
- ◯ 2010
- ◯ 2009
- ◯ 2008
- ◯ 2007
- ◯ 2006
- ◯ 2005
- ◯ 2004
- ◯ 2003
- ◯ 2002
- ◯ 2001
- ◯ 2000
- ◯ 1999
- ◯ 1998
- ◯ 1997
- ◯ 1996
- ◯ 1995
- ◯ 1994
- ◯ 1993
- ◯ 1992
- ◯ 1991
- ◯ 1990
- ◯ 1989
- ◯ 1988
- ◯ 1987
- ◯ 1986
- ◯ 1985
- ◯ 1984
- ◯ 1983
- ◯ 1982
- ◯ 1981
- ◯ 1980
- ◯ Don't Know

The following questions are for the place you lived before that

---

What province?

- ◯ Mpumalanga
- ◯ Gauteng
- ◯ Limpopo
- ◯ Free State
- ◯ KwaZulu Natal
- ◯ Eastern Cape
- ◯ Western Cape
- ◯ Northern Cape
- ◯ North West
- ◯ Other

Please specify: ______________________________

Where within Mpumalanga?	◯ Agincourt study site<br>◯ Barberton<br>◯ Bushbuckridge<br>◯ Dwarsloop<br>◯ Game park Mpumalanga<br>◯ Gemu / Hazyview<br>◯ Graskop/ Pilgrim's rest<br>◯ Kangwane (former homeland)<br>◯ Kiepersol<br>◯ Komatipoort<br>◯ Machadodorp<br>◯ Malelane<br>◯ Mapulaneng / Bushbuckridge<br>◯ Marite / Alexandria<br>◯ Matsulu<br>◯ Middleburg<br>◯ Mkhuhlu<br>◯ Nelspruit<br>◯ Pienaar (Nelspruit)<br>◯ Sabie<br>◯ Thulamahashe<br>◯ White River<br>◯ Witbank<br>◯ Zwelitsha<br>◯ Other Mpumalanga<br>◯ Don't know
Please specify:	__________________________________

Where within Gauteng

- ◯ Akasia
- ◯ Alberton
- ◯ Alexandra
- ◯ Atteridgeville
- ◯ Babelegi
- ◯ Bapsfontein
- ◯ Bashewa
- ◯ Baviaanspoort
- ◯ Bekkersdal
- ◯ Benoni
- ◯ Bhongweni
- ◯ Blue Hills
- ◯ Boipatong
- ◯ Boksburg
- ◯ Bon Accord
- ◯ Bonamanzi Marina and Country Club
- ◯ Bophelong
- ◯ Boschkop
- ◯ Bosplaas Mathabe
- ◯ Bothasgeluk
- ◯ Brakpan
- ◯ Breswol
- ◯ Bronkhorstspruit
- ◯ Bultfontein
- ◯ Carletonville
- ◯ Centurion
- ◯ Chartwell
- ◯ Cheetah Park
- ◯ Chief A Luthuli Park
- ◯ City of Johannesburg NU
- ◯ Clayville
- ◯ Cullinan
- ◯ Dainfern
- ◯ Daveyton
- ◯ Deelkraal Gold Mine
- ◯ Derdepoort
- ◯ Devon A
- ◯ Devon B
- ◯ Diepsloot
- ◯ Dilopye
- ◯ Donkerhoek
- ◯ Doornfontein
- ◯ Doornkraal
- ◯ Downbern
- ◯ Drie Ziek
- ◯ Duduza
- ◯ Dukathole
- ◯ East Driefontein
- ◯ East Village
- ◯ Ebony Park
- ◯ Edenvale
- ◯ Eersterust
- ◯ Ekangala
- ◯ Ekurhuleni NU
- ◯ Elandsfontein
- ◯ Elandsrand
- ◯ Elandsridge
- ◯ Elsburg Gold Mine
- ◯ Emfuleni NU
- ◯ Endicott
- ◯ Ennerdale
- ◯ Etwatwa
- ◯ Evaton
- ◯ Farmall
- ◯ Fochville
- ◯ Ga-Rankuwa
- ◯ Geluksdal
- ◯ Germiston
- ◯ Glen Harvie

◯ Golden Gardens
◯ Goudvlakte West
◯ Green Park
◯ Grootfontein
◯ Grootvlei
◯ Haakdoornboom
◯ Hallgate
◯ Hammanskraal
◯ Harry Gwala
◯ Hartebeesfontein
◯ Hebron
◯ Heidelberg
◯ Hillshaven
◯ Holfontein
◯ Impumelelo
◯ Itsoseng
◯ Ivory Park
◯ Johandeo
◯ Johannesburg
◯ Kaalfontein
◯ Kagiso
◯ Kameeldrift
◯ Kameelfontein
◯ Kameelkraal
◯ Kanana
◯ Kanana Park
◯ Katlehong
◯ Other
◯ Keinfontein
◯ Kekana Garden
◯ Kempton Park
◯ Khutsong
◯ Kleinfontein
◯ Kokosi
◯ Krugersdorp
◯ Kungwini Part 2
◯ Kwa-Thema
◯ Lakeside
◯ Langaville
◯ Lanseria
◯ Laudium
◯ Lawley
◯ Leeufontein
◯ Leeuport
◯ Lehae
◯ Lenasia
◯ Lenasia South
◯ Lenz
◯ Lesedi NU
◯ Letsatsing
◯ Lindelani Village
◯ Lucky 7
◯ Mabopane
◯ Magaliesburg
◯ Majaneng
◯ Makanyaneng
◯ Malatjie
◯ Mamello
◯ Mamelodi
◯ Mandela Village
◯ Marokolong
◯ Mashemong
◯ Mayibuye
◯ Merafong City NU
◯ Meyerton
◯ Midrand
◯ Midvaal NU
◯ Millgate Farm
◯ Mogale City NU
◯ Mohlakeng
◯ Mooiplaas

◯ Munsieville
◯ Nellmapius
◯ New Eersterus
◯ Nigel
◯ Nooitgecht
◯ Nufcor
◯ Oberholzer
◯ Olievenhoutbos
◯ Onverwacht
◯ Orange Farm
◯ Orient Hills
◯ Pebble Rock Golf Village
◯ Phomolong
◯ Poortjie
◯ Pretoria
◯ Rabie Ridge
◯ Ramotse
◯ Randburg
◯ Randfontein
◯ Randfontein NU
◯ Randvaal
◯ Ratanda
◯ Rayton
◯ Refilwe
◯ Rethabiseng
◯ Rietfontein
◯ Rietvallei
◯ Roodeplaat
◯ Roodepoort
◯ Sable Hills Waterfront Estate
◯ Sandton
◯ Saulsville
◯ Seberuberung
◯ Sebokeng
◯ Sharpeville
◯ Simunye
◯ Sonstraal
◯ Soshanguve
◯ Southdene
◯ Soutpan
◯ Soweto
◯ Spaarwater
◯ Springs
◯ Stinkwater
◯ Stretford
◯ Suurman
◯ Temba
◯ Tembisa
◯ The Carousel Casino and Entertainment World
◯ The Hill
◯ The Village
◯ Thembisile
◯ Thinasonke
◯ Tierpoort
◯ Tokoza
◯ Tsakane
◯ Tsebe
◯ Tshepiso
◯ Tshepisong
◯ Tshwane NU
◯ Tweedracht
◯ Tweefontein
◯ Vaal Marina
◯ Vaal Oewer
◯ Vaalbank
◯ Vanderbijlpark
◯ Vastfontein
◯ Venterspost
◯ Vereeniging
◯ Viskuil
◯ Vlakfontein

◯ Vosloorus
◯ Wageenbietjieskop
◯ Walkerville
◯ Wallis Haven
◯ Wallmannsthal
◯ Waterpan
◯ Waterval
◯ Waterworks
◯ Wattville
◯ Wedela
◯ Welverdiend
◯ West Village
◯ West-Driefontein
◯ Western Areas Gold Mine
◯ Western Deep Levels Mine
◯ Westonaria
◯ Westonaria NU
◯ Winterveld
◯ Wolwekrans
◯ Zakariyya Park
◯ Zenzele
◯ Zevenfontein
◯ Zithobeni
◯ Zwartkop
◯ Zwavelpoort

Please specify:

____

Where within Limpopo?

◯ Aganang
◯ Ba-Phalaborwa
◯ Bela-Bela
◯ Blouberg
◯ Elias Motsoaledi
◯ Fetakgomo
◯ Greater Giyani
◯ Greater Letaba
◯ Greater Marble Hall
◯ Greater Tubatse
◯ Greater Tzaneen
◯ Lepelle-Nkumpi
◯ Lephalale
◯ Makhado
◯ Makhuduthamaga
◯ Malamulele
◯ Maruleng
◯ Modimolle
◯ Mogalakwena
◯ Molemole
◯ Mookgopong
◯ Musina
◯ Polokwane
◯ Thabazimbi
◯ Thulamela
◯ Other
◯ Don't know

Please specify:

____

Where within Western Cape?

____

Where within Eastern Cape?	__________________________
Where within North West?	__________________________
Where within KwaZulu Natal?	__________________________
Where within Northern Cape?	__________________________
Where within Free State?	__________________________
(3) Was it rural or urban?	◯ Rural<br>◯ Urban (city or town)<br>◯ Don't know<br>(Rural meaning locations like villages, agricultural areas, or game farms)
(3) When did you start living there? MONTH	◯ January<br>◯ February<br>◯ March<br>◯ April<br>◯ May<br>◯ June<br>◯ July<br>◯ August<br>◯ September<br>◯ October<br>◯ November<br>◯ December<br>◯ Don't know

(3) When did you start living there? YEAR

◯ 2019
◯ 2018
◯ 2017
◯ 2016
◯ 2015
◯ 2014
◯ 2013
◯ 2012
◯ 2011
◯ 2010
◯ 2009
◯ 2008
◯ 2007
◯ 2006
◯ 2005
◯ 2004
◯ 2003
◯ 2002
◯ 2001
◯ 2000
◯ 1999
◯ 1998
◯ 1997
◯ 1996
◯ 1995
◯ 1994
◯ 1993
◯ 1992
◯ 1991
◯ 1990
◯ 1989
◯ 1988
◯ 1987
◯ 1986
◯ 1985
◯ 1984
◯ 1983
◯ 1982
◯ 1981
◯ 1980
◯ Don't Know

(3) What was your main reason for moving to this place of residence?	◯ Job assignment/transfer<br>◯ Retrenchment<br>◯ Better employment opportunities<br>◯ Better accommodation prospects<br>◯ Better educational/training opportunities<br>◯ Closer to school or work<br>◯ Better public/social services<br>◯ Moved with family<br>◯ Joined family or friends<br>◯ Marriage<br>◯ Divorce<br>◯ Death of a family member<br>◯ Family responsibility<br>◯ Attraction to urban life style<br>◯ Evicted from previous residence<br>◯ Not able to pay rent<br>◯ Government resettlement<br>◯ Left to escape violence<br>◯ Other (specify)<br>◯ Dont know
(3) Please specify	__________________________________

(3) What was your main formal/informal occupation in the month BEFORE you moved?

◯ None
◯ Farm work
◯ Domestic work
◯ Construction work
◯ Security work
◯ Cleaning work
◯ Small business owner
◯ Mine work
◯ Teacher
◯ Traditional healer
◯ Health sector (formal)
◯ Game farm/game reserve (e.g. ranger)
◯ Driver
◯ Skilled worker (e.g. plumber, mechanic, electrician)
◯ Cook/ chef/ catering
◯ Unskilled worker (e.g. general labourer)
◯ Artisan (e.g. carpenter, wood carver, weaver)
◯ Waiter/ barman
◯ Informal selling
◯ Small business assistant
◯ Clerical and office work
◯ Cattle herder
◯ Sewing, hairdressing, baking, brewing
◯ Police, soldier, fireman
◯ Petrol attendant
◯ Timber, sawmill, poles
◯ Gardening services
◯ Fieldworker - NGO or university
◯ Art, craft, photography, fashion design
◯ Senior administrator, manager, professional
◯ Priest/pastor
◯ Retail/clerk/sales
◯ Student
◯ Other
◯ Don't know
(If unsure of how to classify, select "Other")

(3) Please specify

____

(3) What was your main formal/informal occupation in the month AFTER you moved?	◯ None<br>◯ Farm work<br>◯ Domestic work<br>◯ Construction work<br>◯ Security work<br>◯ Cleaning work<br>◯ Small business owner<br>◯ Mine work<br>◯ Teacher<br>◯ Traditional healer<br>◯ Health sector (formal)<br>◯ Game farm/game reserve (e.g. ranger)<br>◯ Driver<br>◯ Skilled worker (e.g. plumber, mechanic, electrician)<br>◯ Cook/ chef/ catering<br>◯ Unskilled worker (e.g. general labourer)<br>◯ Artisan (e.g. carpenter, wood carver, weaver)<br>◯ Waiter/ barman<br>◯ Informal selling<br>◯ Small business assistant<br>◯ Clerical and office work<br>◯ Cattle herder<br>◯ Sewing, hairdressing, baking, brewing<br>◯ Police, soldier, fireman<br>◯ Petrol attendant<br>◯ Timber, sawmill, poles<br>◯ Gardening services<br>◯ Fieldworker - NGO or university<br>◯ Art, craft, photography, fashion design<br>◯ Senior administrator, manager, professional<br>◯ Priest/pastor<br>◯ Retail/clerk/sales<br>◯ Student<br>◯ Other<br>◯ Don't know<br>(If unsure of how to classify, select "Other")
(3) Please specify	__________________________________
(3) In what type of place did you live?	◯ Commercial farm<br>◯ Game farm/nature reserve<br>◯ Mine<br>◯ Military base<br>◯ City (inner)<br>◯ City (suburb)<br>◯ Town<br>◯ Village (trust land)<br>◯ Informal settlement (city)<br>◯ Informal settlement (rural)<br>◯ Township (urban)<br>◯ Township (rural)<br>◯ Other<br>◯ Don't know
(3) Please specify	__________________________________

(3) What type of dwelling did you live in?	◯ House, brick or concrete structure on a separate stand<br>◯ Traditional dwelling, hut or structure made of traditional materials<br>◯ Flat or apartment in a block of flats/complex<br>◯ Cluster house in a complex<br>◯ Townhouse<br>◯ Semi-detached house not in a complex<br>◯ House, flat or room separate from main dwelling in backyard<br>◯ Informal dwelling or shack in backyard<br>◯ Informal dwelling NOT in backyard, e.g. in informal squatter settlement or on a farm<br>◯ Room or flat which is part of main dwelling or property<br>◯ Caravan or tent<br>◯ Unit in a retirement home or barracks etc.<br>◯ Other<br>◯ Don't know
(3) Please specify	__________________________________
(3) With?whom did you live at this residence?	☐ Parent(s)<br>☐ Spouse/partner/boyfriend/girlfriend<br>☐ Spouse and children<br>☐ Parent(s) in-law<br>☐ Friends<br>☐ Relatives<br>☐ Countrymen<br>☐ Alone<br>☐ Second family<br>☐ Other<br>☐ Don't know
(3) Please specify	__________________________________
(3) When did you stop living there? MONTH	◯ January<br>◯ February<br>◯ March<br>◯ April<br>◯ May<br>◯ June<br>◯ July<br>◯ August<br>◯ September<br>◯ October<br>◯ November<br>◯ December<br>◯ Don't know

(3) When did you stop living there? YEAR	◯ 2019<br>◯ 2018<br>◯ 2017<br>◯ 2016<br>◯ 2015<br>◯ 2014<br>◯ 2013<br>◯ 2012<br>◯ 2011<br>◯ 2010<br>◯ 2009<br>◯ 2008<br>◯ 2007<br>◯ 2006<br>◯ 2005<br>◯ 2004<br>◯ 2003<br>◯ 2002<br>◯ 2001<br>◯ 2000<br>◯ 1999<br>◯ 1998<br>◯ 1997<br>◯ 1996<br>◯ 1995<br>◯ 1994<br>◯ 1993<br>◯ 1992<br>◯ 1991<br>◯ 1990<br>◯ 1989<br>◯ 1988<br>◯ 1987<br>◯ 1986<br>◯ 1985<br>◯ 1984<br>◯ 1983<br>◯ 1982<br>◯ 1981<br>◯ 1980<br>◯ Don't Know
The following questions are for the place you lived before that	
What province?	◯ Mpumalanga<br>◯ Gauteng<br>◯ Limpopo<br>◯ Free State<br>◯ KwaZulu Natal<br>◯ Eastern Cape<br>◯ Western Cape<br>◯ Northern Cape<br>◯ North West<br>◯ Other
Please specify:	__________________________________

Where within Mpumalanga?

◯ Agincourt study site
◯ Barberton
◯ Bushbuckridge
◯ Dwarsloop
◯ Game park Mpumalanga
◯ Gemu / Hazyview
◯ Graskop/ Pilgrim's rest
◯ Kangwane (former homeland)
◯ Kiepersol
◯ Komatipoort
◯ Machadodorp
◯ Malelane
◯ Mapulaneng / Bushbuckridge
◯ Marite / Alexandria
◯ Matsulu
◯ Middleburg
◯ Mkhuhlu
◯ Nelspruit
◯ Pienaar (Nelspruit)
◯ Sabie
◯ Thulamahashe
◯ White River
◯ Witbank
◯ Zwelitsha
◯ Other Mpumalanga
◯ Don't know

Please specify: __________________________________

Where within Gauteng

〇 Akasia
〇 Alberton
〇 Alexandra
〇 Atteridgeville
〇 Babelegi
〇 Bapsfontein
〇 Bashewa
〇 Baviaanspoort
〇 Bekkersdal
〇 Benoni
〇 Bhongweni
〇 Blue Hills
〇 Boipatong
〇 Boksburg
〇 Bon Accord
〇 Bonamanzi Marina and Country Club
〇 Bophelong
〇 Boschkop
〇 Bosplaas Mathabe
〇 Bothasgeluk
〇 Brakpan
〇 Breswol
〇 Bronkhorstspruit
〇 Bultfontein
〇 Carletonville
〇 Centurion
〇 Chartwell
〇 Cheetah Park
〇 Chief A Luthuli Park
〇 City of Johannesburg NU
〇 Clayville
〇 Cullinan
〇 Dainfern
〇 Daveyton
〇 Deelkraal Gold Mine
〇 Derdepoort
〇 Devon A
〇 Devon B
〇 Diepsloot
〇 Dilopye
〇 Donkerhoek
〇 Doornfontein
〇 Doornkraal
〇 Downbern
〇 Drie Ziek
〇 Duduza
〇 Dukathole
〇 East Driefontein
〇 East Village
〇 Ebony Park
〇 Edenvale
〇 Eersterust
〇 Ekangala
〇 Ekurhuleni NU
〇 Elandsfontein
〇 Elandsrand
〇 Elandsridge
〇 Elsburg Gold Mine
〇 Emfuleni NU
〇 Endicott
〇 Ennerdale
〇 Etwatwa
〇 Evaton
〇 Farmall
〇 Fochville
〇 Ga-Rankuwa
〇 Geluksdal
〇 Germiston
〇 Glen Harvie

◯ Golden Gardens
◯ Goudvlakte West
◯ Green Park
◯ Grootfontein
◯ Grootvlei
◯ Haakdoornboom
◯ Hallgate
◯ Hammanskraal
◯ Harry Gwala
◯ Hartebeesfontein
◯ Hebron
◯ Heidelberg
◯ Hillshaven
◯ Holfontein
◯ Impumelelo
◯ Itsoseng
◯ Ivory Park
◯ Johandeo
◯ Johannesburg
◯ Kaalfontein
◯ Kagiso
◯ Kameeldrift
◯ Kameelfontein
◯ Kameelkraal
◯ Kanana
◯ Kanana Park
◯ Katlehong
◯ Other
◯ Keinfontein
◯ Kekana Garden
◯ Kempton Park
◯ Khutsong
◯ Kleinfontein
◯ Kokosi
◯ Krugersdorp
◯ Kungwini Part 2
◯ Kwa-Thema
◯ Lakeside
◯ Langaville
◯ Lanseria
◯ Laudium
◯ Lawley
◯ Leeufontein
◯ Leeuport
◯ Lehae
◯ Lenasia
◯ Lenasia South
◯ Lenz
◯ Lesedi NU
◯ Letsatsing
◯ Lindelani Village
◯ Lucky 7
◯ Mabopane
◯ Magaliesburg
◯ Majaneng
◯ Makanyaneng
◯ Malatjie
◯ Mamello
◯ Mamelodi
◯ Mandela Village
◯ Marokolong
◯ Mashemong
◯ Mayibuye
◯ Merafong City NU
◯ Meyerton
◯ Midrand
◯ Midvaal NU
◯ Millgate Farm
◯ Mogale City NU
◯ Mohlakeng
◯ Mooiplaas

- ◯ Munsieville
- ◯ Nellmapius
- ◯ New Eersterus
- ◯ Nigel
- ◯ Nooitgecht
- ◯ Nufcor
- ◯ Oberholzer
- ◯ Olievenhoutbos
- ◯ Onverwacht
- ◯ Orange Farm
- ◯ Orient Hills
- ◯ Pebble Rock Golf Village
- ◯ Phomolong
- ◯ Poortjie
- ◯ Pretoria
- ◯ Rabie Ridge
- ◯ Ramotse
- ◯ Randburg
- ◯ Randfontein
- ◯ Randfontein NU
- ◯ Randvaal
- ◯ Ratanda
- ◯ Rayton
- ◯ Refilwe
- ◯ Rethabiseng
- ◯ Rietfontein
- ◯ Rietvallei
- ◯ Roodeplaat
- ◯ Roodepoort
- ◯ Sable Hills Waterfront Estate
- ◯ Sandton
- ◯ Saulsville
- ◯ Seberuberung
- ◯ Sebokeng
- ◯ Sharpeville
- ◯ Simunye
- ◯ Sonstraal
- ◯ Soshanguve
- ◯ Southdene
- ◯ Soutpan
- ◯ Soweto
- ◯ Spaarwater
- ◯ Springs
- ◯ Stinkwater
- ◯ Stretford
- ◯ Suurman
- ◯ Temba
- ◯ Tembisa
- ◯ The Carousel Casino and Entertainment World
- ◯ The Hill
- ◯ The Village
- ◯ Thembisile
- ◯ Thinasonke
- ◯ Tierpoort
- ◯ Tokoza
- ◯ Tsakane
- ◯ Tsebe
- ◯ Tshepiso
- ◯ Tshepisong
- ◯ Tshwane NU
- ◯ Tweedracht
- ◯ Tweefontein
- ◯ Vaal Marina
- ◯ Vaal Oewer
- ◯ Vaalbank
- ◯ Vanderbijlpark
- ◯ Vastfontein
- ◯ Venterspost
- ◯ Vereeniging
- ◯ Viskuil
- ◯ Vlakfontein

◯ Vosloorus
◯ Wageenbietjieskop
◯ Walkerville
◯ Wallis Haven
◯ Wallmannsthal
◯ Waterpan
◯ Waterval
◯ Waterworks
◯ Wattville
◯ Wedela
◯ Welverdiend
◯ West Village
◯ West-Driefontein
◯ Western Areas Gold Mine
◯ Western Deep Levels Mine
◯ Westonaria
◯ Westonaria NU
◯ Winterveld
◯ Wolwekrans
◯ Zakariyya Park
◯ Zenzele
◯ Zevenfontein
◯ Zithobeni
◯ Zwartkop
◯ Zwavelpoort

Please specify:

____

Where within Limpopo?

◯ Aganang
◯ Ba-Phalaborwa
◯ Bela-Bela
◯ Blouberg
◯ Elias Motsoaledi
◯ Fetakgomo
◯ Greater Giyani
◯ Greater Letaba
◯ Greater Marble Hall
◯ Greater Tubatse
◯ Greater Tzaneen
◯ Lepelle-Nkumpi
◯ Lephalale
◯ Makhado
◯ Makhuduthamaga
◯ Malamulele
◯ Maruleng
◯ Modimolle
◯ Mogalakwena
◯ Molemole
◯ Mookgopong
◯ Musina
◯ Polokwane
◯ Thabazimbi
◯ Thulamela
◯ Other
◯ Don't know

Please specify:

____

Where within Western Cape?

____

Where within Eastern Cape? __________________________________

Where within North West? __________________________________

Where within KwaZulu Natal? __________________________________

Where within Northern Cape? __________________________________

Where within Free State? __________________________________

(4) Was it rural or urban?

- ◯ Rural
- ◯ Urban (city or town)
- ◯ Don't know

(Rural meaning locations like villages, agricultural areas, or game farms)

(4) When did you start living there? MONTH

- ◯ January
- ◯ February
- ◯ March
- ◯ April
- ◯ May
- ◯ June
- ◯ July
- ◯ August
- ◯ September
- ◯ October
- ◯ November
- ◯ December
- ◯ Don't know

(4) When did you start living there? YEAR

◯ 2019
◯ 2018
◯ 2017
◯ 2016
◯ 2015
◯ 2014
◯ 2013
◯ 2012
◯ 2011
◯ 2010
◯ 2009
◯ 2008
◯ 2007
◯ 2006
◯ 2005
◯ 2004
◯ 2003
◯ 2002
◯ 2001
◯ 2000
◯ 1999
◯ 1998
◯ 1997
◯ 1996
◯ 1995
◯ 1994
◯ 1993
◯ 1992
◯ 1991
◯ 1990
◯ 1989
◯ 1988
◯ 1987
◯ 1986
◯ 1985
◯ 1984
◯ 1983
◯ 1982
◯ 1981
◯ 1980
◯ Don't Know

(4) What was your main reason for moving to this place of residence?	◯ Job assignment/transfer<br>◯ Retrenchment<br>◯ Better employment opportunities<br>◯ Better accommodation prospects<br>◯ Better educational/training opportunities<br>◯ Closer to school or work<br>◯ Better public/social services<br>◯ Moved with family<br>◯ Joined family or friends<br>◯ Marriage<br>◯ Divorce<br>◯ Death of a family member<br>◯ Family responsibility<br>◯ Attraction to urban life style<br>◯ Evicted from previous residence<br>◯ Not able to pay rent<br>◯ Government resettlement<br>◯ Left to escape violence<br>◯ Other (specify)<br>◯ Dont know
(4) Please specify	__________________________________

(4) What was your main formal/informal occupation in the month BEFORE you moved?	◯ None<br>◯ Farm work<br>◯ Domestic work<br>◯ Construction work<br>◯ Security work<br>◯ Cleaning work<br>◯ Small business owner<br>◯ Mine work<br>◯ Teacher<br>◯ Traditional healer<br>◯ Health sector (formal)<br>◯ Game farm/game reserve (e.g. ranger)<br>◯ Driver<br>◯ Skilled worker (e.g. plumber, mechanic, electrician)<br>◯ Cook/ chef/ catering<br>◯ Unskilled worker (e.g. general labourer)<br>◯ Artisan (e.g. carpenter, wood carver, weaver)<br>◯ Waiter/ barman<br>◯ Informal selling<br>◯ Small business assistant<br>◯ Clerical and office work<br>◯ Cattle herder<br>◯ Sewing, hairdressing, baking, brewing<br>◯ Police, soldier, fireman<br>◯ Petrol attendant<br>◯ Timber, sawmill, poles<br>◯ Gardening services<br>◯ Fieldworker - NGO or university<br>◯ Art, craft, photography, fashion design<br>◯ Senior administrator, manager, professional<br>◯ Priest/pastor<br>◯ Retail/clerk/sales<br>◯ Student<br>◯ Other<br>◯ Don't know<br>(If unsure of how to classify, select "Other")
(4) Please specify	__________________________________

(4) What was your main formal/informal occupation in the month AFTER you moved?	◯ None<br>◯ Farm work<br>◯ Domestic work<br>◯ Construction work<br>◯ Security work<br>◯ Cleaning work<br>◯ Small business owner<br>◯ Mine work<br>◯ Teacher<br>◯ Traditional healer<br>◯ Health sector (formal)<br>◯ Game farm/game reserve (e.g. ranger)<br>◯ Driver<br>◯ Skilled worker (e.g. plumber, mechanic, electrician)<br>◯ Cook/ chef/ catering<br>◯ Unskilled worker (e.g. general labourer)<br>◯ Artisan (e.g. carpenter, wood carver, weaver)<br>◯ Waiter/ barman<br>◯ Informal selling<br>◯ Small business assistant<br>◯ Clerical and office work<br>◯ Cattle herder<br>◯ Sewing, hairdressing, baking, brewing<br>◯ Police, soldier, fireman<br>◯ Petrol attendant<br>◯ Timber, sawmill, poles<br>◯ Gardening services<br>◯ Fieldworker - NGO or university<br>◯ Art, craft, photography, fashion design<br>◯ Senior administrator, manager, professional<br>◯ Priest/pastor<br>◯ Retail/clerk/sales<br>◯ Student<br>◯ Other<br>◯ Don't know<br>(If unsure of how to classify, select "Other")
(4) Please specify	__________________________________
(4) In what type of place did you live?	◯ Commercial farm<br>◯ Game farm/nature reserve<br>◯ Mine<br>◯ Military base<br>◯ City (inner)<br>◯ City (suburb)<br>◯ Town<br>◯ Village (trust land)<br>◯ Informal settlement (city)<br>◯ Informal settlement (rural)<br>◯ Township (urban)<br>◯ Township (rural)<br>◯ Other<br>◯ Don't know
(4) Please specify	__________________________________

(4) What type of dwelling did you live in?

- ◯ House, brick or concrete structure on a separate stand
- ◯ Traditional dwelling, hut or structure made of traditional materials
- ◯ Flat or apartment in a block of flats/complex
- ◯ Cluster house in a complex
- ◯ Townhouse
- ◯ Semi-detached house not in a complex
- ◯ House, flat or room separate from main dwelling in backyard
- ◯ Informal dwelling or shack in backyard
- ◯ Informal dwelling NOT in backyard, e.g. in informal squatter settlement or on a farm
- ◯ Room or flat which is part of main dwelling or property
- ◯ Caravan or tent
- ◯ Unit in a retirement home or barracks etc.
- ◯ Other
- ◯ Don't know

(4) Please specify

____

(4) With?whom did you live at this residence?

- ☐ Parent(s)
- ☐ Spouse/partner/boyfriend/girlfriend
- ☐ Spouse and children
- ☐ Parent(s) in-law
- ☐ Friends
- ☐ Relatives
- ☐ Countrymen
- ☐ Alone
- ☐ Second family
- ☐ Other
- ☐ Don't know

(4) Please specify

____

(4) When did you stop living there? MONTH

- ◯ January
- ◯ February
- ◯ March
- ◯ April
- ◯ May
- ◯ June
- ◯ July
- ◯ August
- ◯ September
- ◯ October
- ◯ November
- ◯ December
- ◯ Don't know

(4) When did you stop living there? YEAR	◯ 2019<br>◯ 2018<br>◯ 2017<br>◯ 2016<br>◯ 2015<br>◯ 2014<br>◯ 2013<br>◯ 2012<br>◯ 2011<br>◯ 2010<br>◯ 2009<br>◯ 2008<br>◯ 2007<br>◯ 2006<br>◯ 2005<br>◯ 2004<br>◯ 2003<br>◯ 2002<br>◯ 2001<br>◯ 2000<br>◯ 1999<br>◯ 1998<br>◯ 1997<br>◯ 1996<br>◯ 1995<br>◯ 1994<br>◯ 1993<br>◯ 1992<br>◯ 1991<br>◯ 1990<br>◯ 1989<br>◯ 1988<br>◯ 1987<br>◯ 1986<br>◯ 1985<br>◯ 1984<br>◯ 1983<br>◯ 1982<br>◯ 1981<br>◯ 1980<br>◯ Don't Know
The following questions are for the place you lived before that	
What province?	◯ Mpumalanga<br>◯ Gauteng<br>◯ Limpopo<br>◯ Free State<br>◯ KwaZulu Natal<br>◯ Eastern Cape<br>◯ Western Cape<br>◯ Northern Cape<br>◯ North West<br>◯ Other
Please specify:	__________________________________

Where within Mpumalanga?

- ◯ Agincourt study site
- ◯ Barberton
- ◯ Bushbuckridge
- ◯ Dwarsloop
- ◯ Game park Mpumalanga
- ◯ Gemu / Hazyview
- ◯ Graskop/ Pilgrim's rest
- ◯ Kangwane (former homeland)
- ◯ Kiepersol
- ◯ Komatipoort
- ◯ Machadodorp
- ◯ Malelane
- ◯ Mapulaneng / Bushbuckridge
- ◯ Marite / Alexandria
- ◯ Matsulu
- ◯ Middleburg
- ◯ Mkhuhlu
- ◯ Nelspruit
- ◯ Pienaar (Nelspruit)
- ◯ Sabie
- ◯ Thulamahashe
- ◯ White River
- ◯ Witbank
- ◯ Zwelitsha
- ◯ Other Mpumalanga
- ◯ Don't know

Please specify: __________________________

Where within Gauteng

- ◯ Akasia
- ◯ Alberton
- ◯ Alexandra
- ◯ Atteridgeville
- ◯ Babelegi
- ◯ Bapsfontein
- ◯ Bashewa
- ◯ Baviaanspoort
- ◯ Bekkersdal
- ◯ Benoni
- ◯ Bhongweni
- ◯ Blue Hills
- ◯ Boipatong
- ◯ Boksburg
- ◯ Bon Accord
- ◯ Bonamanzi Marina and Country Club
- ◯ Bophelong
- ◯ Boschkop
- ◯ Bosplaas Mathabe
- ◯ Bothasgeluk
- ◯ Brakpan
- ◯ Breswol
- ◯ Bronkhorstspruit
- ◯ Bultfontein
- ◯ Carletonville
- ◯ Centurion
- ◯ Chartwell
- ◯ Cheetah Park
- ◯ Chief A Luthuli Park
- ◯ City of Johannesburg NU
- ◯ Clayville
- ◯ Cullinan
- ◯ Dainfern
- ◯ Daveyton
- ◯ Deelkraal Gold Mine
- ◯ Derdepoort
- ◯ Devon A
- ◯ Devon B
- ◯ Diepsloot
- ◯ Dilopye
- ◯ Donkerhoek
- ◯ Doornfontein
- ◯ Doornkraal
- ◯ Downbern
- ◯ Drie Ziek
- ◯ Duduza
- ◯ Dukathole
- ◯ East Driefontein
- ◯ East Village
- ◯ Ebony Park
- ◯ Edenvale
- ◯ Eersterust
- ◯ Ekangala
- ◯ Ekurhuleni NU
- ◯ Elandsfontein
- ◯ Elandsrand
- ◯ Elandsridge
- ◯ Elsburg Gold Mine
- ◯ Emfuleni NU
- ◯ Endicott
- ◯ Ennerdale
- ◯ Etwatwa
- ◯ Evaton
- ◯ Farmall
- ◯ Fochville
- ◯ Ga-Rankuwa
- ◯ Geluksdal
- ◯ Germiston
- ◯ Glen Harvie

◯ Golden Gardens
◯ Goudvlakte West
◯ Green Park
◯ Grootfontein
◯ Grootvlei
◯ Haakdoornboom
◯ Hallgate
◯ Hammanskraal
◯ Harry Gwala
◯ Hartebeesfontein
◯ Hebron
◯ Heidelberg
◯ Hillshaven
◯ Holfontein
◯ Impumelelo
◯ Itsoseng
◯ Ivory Park
◯ Johandeo
◯ Johannesburg
◯ Kaalfontein
◯ Kagiso
◯ Kameeldrift
◯ Kameelfontein
◯ Kameelkraal
◯ Kanana
◯ Kanana Park
◯ Katlehong
◯ Other
◯ Keinfontein
◯ Kekana Garden
◯ Kempton Park
◯ Khutsong
◯ Kleinfontein
◯ Kokosi
◯ Krugersdorp
◯ Kungwini Part 2
◯ Kwa-Thema
◯ Lakeside
◯ Langaville
◯ Lanseria
◯ Laudium
◯ Lawley
◯ Leeufontein
◯ Leeuport
◯ Lehae
◯ Lenasia
◯ Lenasia South
◯ Lenz
◯ Lesedi NU
◯ Letsatsing
◯ Lindelani Village
◯ Lucky 7
◯ Mabopane
◯ Magaliesburg
◯ Majaneng
◯ Makanyaneng
◯ Malatjie
◯ Mamello
◯ Mamelodi
◯ Mandela Village
◯ Marokolong
◯ Mashemong
◯ Mayibuye
◯ Merafong City NU
◯ Meyerton
◯ Midrand
◯ Midvaal NU
◯ Millgate Farm
◯ Mogale City NU
◯ Mohlakeng
◯ Mooiplaas

◯ Munsieville
◯ Nellmapius
◯ New Eersterus
◯ Nigel
◯ Nooitgecht
◯ Nufcor
◯ Oberholzer
◯ Olievenhoutbos
◯ Onverwacht
◯ Orange Farm
◯ Orient Hills
◯ Pebble Rock Golf Village
◯ Phomolong
◯ Poortjie
◯ Pretoria
◯ Rabie Ridge
◯ Ramotse
◯ Randburg
◯ Randfontein
◯ Randfontein NU
◯ Randvaal
◯ Ratanda
◯ Rayton
◯ Refilwe
◯ Rethabiseng
◯ Rietfontein
◯ Rietvallei
◯ Roodeplaat
◯ Roodepoort
◯ Sable Hills Waterfront Estate
◯ Sandton
◯ Saulsville
◯ Seberuberung
◯ Sebokeng
◯ Sharpeville
◯ Simunye
◯ Sonstraal
◯ Soshanguve
◯ Southdene
◯ Soutpan
◯ Soweto
◯ Spaarwater
◯ Springs
◯ Stinkwater
◯ Stretford
◯ Suurman
◯ Temba
◯ Tembisa
◯ The Carousel Casino and Entertainment World
◯ The Hill
◯ The Village
◯ Thembisile
◯ Thinasonke
◯ Tierpoort
◯ Tokoza
◯ Tsakane
◯ Tsebe
◯ Tshepiso
◯ Tshepisong
◯ Tshwane NU
◯ Tweedracht
◯ Tweefontein
◯ Vaal Marina
◯ Vaal Oewer
◯ Vaalbank
◯ Vanderbijlpark
◯ Vastfontein
◯ Venterspost
◯ Vereeniging
◯ Viskuil
◯ Vlakfontein

- ◯ Vosloorus
- ◯ Wageenbietjieskop
- ◯ Walkerville
- ◯ Wallis Haven
- ◯ Wallmannsthal
- ◯ Waterpan
- ◯ Waterval
- ◯ Waterworks
- ◯ Wattville
- ◯ Wedela
- ◯ Welverdiend
- ◯ West Village
- ◯ West-Driefontein
- ◯ Western Areas Gold Mine
- ◯ Western Deep Levels Mine
- ◯ Westonaria
- ◯ Westonaria NU
- ◯ Winterveld
- ◯ Wolwekrans
- ◯ Zakariyya Park
- ◯ Zenzele
- ◯ Zevenfontein
- ◯ Zithobeni
- ◯ Zwartkop
- ◯ Zwavelpoort

Please specify:

____

Where within Limpopo?

- ◯ Aganang
- ◯ Ba-Phalaborwa
- ◯ Bela-Bela
- ◯ Blouberg
- ◯ Elias Motsoaledi
- ◯ Fetakgomo
- ◯ Greater Giyani
- ◯ Greater Letaba
- ◯ Greater Marble Hall
- ◯ Greater Tubatse
- ◯ Greater Tzaneen
- ◯ Lepelle-Nkumpi
- ◯ Lephalale
- ◯ Makhado
- ◯ Makhuduthamaga
- ◯ Malamulele
- ◯ Maruleng
- ◯ Modimolle
- ◯ Mogalakwena
- ◯ Molemole
- ◯ Mookgopong
- ◯ Musina
- ◯ Polokwane
- ◯ Thabazimbi
- ◯ Thulamela
- ◯ Other
- ◯ Don't know

Please specify:

____

Where within Western Cape?

____

Where within Eastern Cape? __________________________________

Where within North West? __________________________________

Where within KwaZulu Natal? __________________________________

Where within Northern Cape? __________________________________

Where within Free State? __________________________________

(5) Was it rural or urban?

- ◯ Rural
- ◯ Urban (city or town)
- ◯ Don't know

(Rural meaning locations like villages, agricultural areas, or game farms)

(5) When did you start living there? MONTH

- ◯ January
- ◯ February
- ◯ March
- ◯ April
- ◯ May
- ◯ June
- ◯ July
- ◯ August
- ◯ September
- ◯ October
- ◯ November
- ◯ December
- ◯ Don't know

(5) When did you start living there? YEAR

- ◯ 2019
- ◯ 2018
- ◯ 2017
- ◯ 2016
- ◯ 2015
- ◯ 2014
- ◯ 2013
- ◯ 2012
- ◯ 2011
- ◯ 2010
- ◯ 2009
- ◯ 2008
- ◯ 2007
- ◯ 2006
- ◯ 2005
- ◯ 2004
- ◯ 2003
- ◯ 2002
- ◯ 2001
- ◯ 2000
- ◯ 1999
- ◯ 1998
- ◯ 1997
- ◯ 1996
- ◯ 1995
- ◯ 1994
- ◯ 1993
- ◯ 1992
- ◯ 1991
- ◯ 1990
- ◯ 1989
- ◯ 1988
- ◯ 1987
- ◯ 1986
- ◯ 1985
- ◯ 1984
- ◯ 1983
- ◯ 1982
- ◯ 1981
- ◯ 1980
- ◯ Don't Know

(5) What was your main reason for moving to this place of residence?

◯ Job assignment/transfer
◯ Retrenchment
◯ Better employment opportunities
◯ Better accommodation prospects
◯ Better educational/training opportunities
◯ Closer to school or work
◯ Better public/social services
◯ Moved with family
◯ Joined family or friends
◯ Marriage
◯ Divorce
◯ Death of a family member
◯ Family responsibility
◯ Attraction to urban life style
◯ Evicted from previous residence
◯ Not able to pay rent
◯ Government resettlement
◯ Left to escape violence
◯ Other (specify)
◯ Dont know

(5) Please specify

____

(5) What was your main formal/informal occupation in the month BEFORE you moved?

- ◯ None
- ◯ Farm work
- ◯ Domestic work
- ◯ Construction work
- ◯ Security work
- ◯ Cleaning work
- ◯ Small business owner
- ◯ Mine work
- ◯ Teacher
- ◯ Traditional healer
- ◯ Health sector (formal)
- ◯ Game farm/game reserve (e.g. ranger)
- ◯ Driver
- ◯ Skilled worker (e.g. plumber, mechanic, electrician)
- ◯ Cook/ chef/ catering
- ◯ Unskilled worker (e.g. general labourer)
- ◯ Artisan (e.g. carpenter, wood carver, weaver)
- ◯ Waiter/ barman
- ◯ Informal selling
- ◯ Small business assistant
- ◯ Clerical and office work
- ◯ Cattle herder
- ◯ Sewing, hairdressing, baking, brewing
- ◯ Police, soldier, fireman
- ◯ Petrol attendant
- ◯ Timber, sawmill, poles
- ◯ Gardening services
- ◯ Fieldworker - NGO or university
- ◯ Art, craft, photography, fashion design
- ◯ Senior administrator, manager, professional
- ◯ Priest/pastor
- ◯ Retail/clerk/sales
- ◯ Student
- ◯ Other
- ◯ Don't know

(If unsure of how to classify, select "Other")

---

(5) Please specify

____

(5) What was your main formal/informal occupation in the month AFTER you moved?	◯ None<br>◯ Farm work<br>◯ Domestic work<br>◯ Construction work<br>◯ Security work<br>◯ Cleaning work<br>◯ Small business owner<br>◯ Mine work<br>◯ Teacher<br>◯ Traditional healer<br>◯ Health sector (formal)<br>◯ Game farm/game reserve (e.g. ranger)<br>◯ Driver<br>◯ Skilled worker (e.g. plumber, mechanic, electrician)<br>◯ Cook/ chef/ catering<br>◯ Unskilled worker (e.g. general labourer)<br>◯ Artisan (e.g. carpenter, wood carver, weaver)<br>◯ Waiter/ barman<br>◯ Informal selling<br>◯ Small business assistant<br>◯ Clerical and office work<br>◯ Cattle herder<br>◯ Sewing, hairdressing, baking, brewing<br>◯ Police, soldier, fireman<br>◯ Petrol attendant<br>◯ Timber, sawmill, poles<br>◯ Gardening services<br>◯ Fieldworker - NGO or university<br>◯ Art, craft, photography, fashion design<br>◯ Senior administrator, manager, professional<br>◯ Priest/pastor<br>◯ Retail/clerk/sales<br>◯ Student<br>◯ Other<br>◯ Don't know<br>(If unsure of how to classify, select "Other")
(5) Please specify	__________________________________
(5) In what type of place did you live?	◯ Commercial farm<br>◯ Game farm/nature reserve<br>◯ Mine<br>◯ Military base<br>◯ City (inner)<br>◯ City (suburb)<br>◯ Town<br>◯ Village (trust land)<br>◯ Informal settlement (city)<br>◯ Informal settlement (rural)<br>◯ Township (urban)<br>◯ Township (rural)<br>◯ Other<br>◯ Don't know
(5) Please specify	__________________________________

(5) What type of dwelling did you live in?

- ◯ House, brick or concrete structure on a separate stand
- ◯ Traditional dwelling, hut or structure made of traditional materials
- ◯ Flat or apartment in a block of flats/complex
- ◯ Cluster house in a complex
- ◯ Townhouse
- ◯ Semi-detached house not in a complex
- ◯ House, flat or room separate from main dwelling in backyard
- ◯ Informal dwelling or shack in backyard
- ◯ Informal dwelling NOT in backyard, e.g. in informal squatter settlement or on a farm
- ◯ Room or flat which is part of main dwelling or property
- ◯ Caravan or tent
- ◯ Unit in a retirement home or barracks etc.
- ◯ Other
- ◯ Don't know

(5) Please specify

____

(5) With?whom did you live at this residence?

- ☐ Parent(s)
- ☐ Spouse/partner/boyfriend/girlfriend
- ☐ Spouse and children
- ☐ Parent(s) in-law
- ☐ Friends
- ☐ Relatives
- ☐ Countrymen
- ☐ Alone
- ☐ Second family
- ☐ Other
- ☐ Don't know

(5) Please specify

____

(5) When did you stop living there? MONTH

- ◯ January
- ◯ February
- ◯ March
- ◯ April
- ◯ May
- ◯ June
- ◯ July
- ◯ August
- ◯ September
- ◯ October
- ◯ November
- ◯ December
- ◯ Don't know

(5) When did you stop living there? YEAR

- ◯ 2019
- ◯ 2018
- ◯ 2017
- ◯ 2016
- ◯ 2015
- ◯ 2014
- ◯ 2013
- ◯ 2012
- ◯ 2011
- ◯ 2010
- ◯ 2009
- ◯ 2008
- ◯ 2007
- ◯ 2006
- ◯ 2005
- ◯ 2004
- ◯ 2003
- ◯ 2002
- ◯ 2001
- ◯ 2000
- ◯ 1999
- ◯ 1998
- ◯ 1997
- ◯ 1996
- ◯ 1995
- ◯ 1994
- ◯ 1993
- ◯ 1992
- ◯ 1991
- ◯ 1990
- ◯ 1989
- ◯ 1988
- ◯ 1987
- ◯ 1986
- ◯ 1985
- ◯ 1984
- ◯ 1983
- ◯ 1982
- ◯ 1981
- ◯ 1980
- ◯ Don't Know

The following questions are for the place you lived before that

What province?

- ◯ Mpumalanga
- ◯ Gauteng
- ◯ Limpopo
- ◯ Free State
- ◯ KwaZulu Natal
- ◯ Eastern Cape
- ◯ Western Cape
- ◯ Northern Cape
- ◯ North West
- ◯ Other

Please specify: ______________________________

Where within Mpumalanga?	◯ Agincourt study site<br>◯ Barberton<br>◯ Bushbuckridge<br>◯ Dwarsloop<br>◯ Game park Mpumalanga<br>◯ Gemu / Hazyview<br>◯ Graskop/ Pilgrim's rest<br>◯ Kangwane (former homeland)<br>◯ Kiepersol<br>◯ Komatipoort<br>◯ Machadodorp<br>◯ Malelane<br>◯ Mapulaneng / Bushbuckridge<br>◯ Marite / Alexandria<br>◯ Matsulu<br>◯ Middleburg<br>◯ Mkhuhlu<br>◯ Nelspruit<br>◯ Pienaar (Nelspruit)<br>◯ Sabie<br>◯ Thulamahashe<br>◯ White River<br>◯ Witbank<br>◯ Zwelitsha<br>◯ Other Mpumalanga<br>◯ Don't know
Please specify:	__________________________________

Where within Gauteng

◯ Akasia
◯ Alberton
◯ Alexandra
◯ Atteridgeville
◯ Babelegi
◯ Bapsfontein
◯ Bashewa
◯ Baviaanspoort
◯ Bekkersdal
◯ Benoni
◯ Bhongweni
◯ Blue Hills
◯ Boipatong
◯ Boksburg
◯ Bon Accord
◯ Bonamanzi Marina and Country Club
◯ Bophelong
◯ Boschkop
◯ Bosplaas Mathabe
◯ Bothasgeluk
◯ Brakpan
◯ Breswol
◯ Bronkhorstspruit
◯ Bultfontein
◯ Carletonville
◯ Centurion
◯ Chartwell
◯ Cheetah Park
◯ Chief A Luthuli Park
◯ City of Johannesburg NU
◯ Clayville
◯ Cullinan
◯ Dainfern
◯ Daveyton
◯ Deelkraal Gold Mine
◯ Derdepoort
◯ Devon A
◯ Devon B
◯ Diepsloot
◯ Dilopye
◯ Donkerhoek
◯ Doornfontein
◯ Doornkraal
◯ Downbern
◯ Drie Ziek
◯ Duduza
◯ Dukathole
◯ East Driefontein
◯ East Village
◯ Ebony Park
◯ Edenvale
◯ Eersterust
◯ Ekangala
◯ Ekurhuleni NU
◯ Elandsfontein
◯ Elandsrand
◯ Elandsridge
◯ Elsburg Gold Mine
◯ Emfuleni NU
◯ Endicott
◯ Ennerdale
◯ Etwatwa
◯ Evaton
◯ Farmall
◯ Fochville
◯ Ga-Rankuwa
◯ Geluksdal
◯ Germiston
◯ Glen Harvie

◯ Golden Gardens
◯ Goudvlakte West
◯ Green Park
◯ Grootfontein
◯ Grootvlei
◯ Haakdoornboom
◯ Hallgate
◯ Hammanskraal
◯ Harry Gwala
◯ Hartebeesfontein
◯ Hebron
◯ Heidelberg
◯ Hillshaven
◯ Holfontein
◯ Impumelelo
◯ Itsoseng
◯ Ivory Park
◯ Johandeo
◯ Johannesburg
◯ Kaalfontein
◯ Kagiso
◯ Kameeldrift
◯ Kameelfontein
◯ Kameelkraal
◯ Kanana
◯ Kanana Park
◯ Katlehong
◯ Other
◯ Keinfontein
◯ Kekana Garden
◯ Kempton Park
◯ Khutsong
◯ Kleinfontein
◯ Kokosi
◯ Krugersdorp
◯ Kungwini Part 2
◯ Kwa-Thema
◯ Lakeside
◯ Langaville
◯ Lanseria
◯ Laudium
◯ Lawley
◯ Leeufontein
◯ Leeuport
◯ Lehae
◯ Lenasia
◯ Lenasia South
◯ Lenz
◯ Lesedi NU
◯ Letsatsing
◯ Lindelani Village
◯ Lucky 7
◯ Mabopane
◯ Magaliesburg
◯ Majaneng
◯ Makanyaneng
◯ Malatjie
◯ Mamello
◯ Mamelodi
◯ Mandela Village
◯ Marokolong
◯ Mashemong
◯ Mayibuye
◯ Merafong City NU
◯ Meyerton
◯ Midrand
◯ Midvaal NU
◯ Millgate Farm
◯ Mogale City NU
◯ Mohlakeng
◯ Mooiplaas

◯ Munsieville
◯ Nellmapius
◯ New Eersterus
◯ Nigel
◯ Nooitgecht
◯ Nufcor
◯ Oberholzer
◯ Olievenhoutbos
◯ Onverwacht
◯ Orange Farm
◯ Orient Hills
◯ Pebble Rock Golf Village
◯ Phomolong
◯ Poortjie
◯ Pretoria
◯ Rabie Ridge
◯ Ramotse
◯ Randburg
◯ Randfontein
◯ Randfontein NU
◯ Randvaal
◯ Ratanda
◯ Rayton
◯ Refilwe
◯ Rethabiseng
◯ Rietfontein
◯ Rietvallei
◯ Roodeplaat
◯ Roodepoort
◯ Sable Hills Waterfront Estate
◯ Sandton
◯ Saulsville
◯ Seberuberung
◯ Sebokeng
◯ Sharpeville
◯ Simunye
◯ Sonstraal
◯ Soshanguve
◯ Southdene
◯ Soutpan
◯ Soweto
◯ Spaarwater
◯ Springs
◯ Stinkwater
◯ Stretford
◯ Suurman
◯ Temba
◯ Tembisa
◯ The Carousel Casino and Entertainment World
◯ The Hill
◯ The Village
◯ Thembisile
◯ Thinasonke
◯ Tierpoort
◯ Tokoza
◯ Tsakane
◯ Tsebe
◯ Tshepiso
◯ Tshepisong
◯ Tshwane NU
◯ Tweedracht
◯ Tweefontein
◯ Vaal Marina
◯ Vaal Oewer
◯ Vaalbank
◯ Vanderbijlpark
◯ Vastfontein
◯ Venterspost
◯ Vereeniging
◯ Viskuil
◯ Vlakfontein

	◯ Vosloorus<br>◯ Wageenbietjieskop<br>◯ Walkerville<br>◯ Wallis Haven<br>◯ Wallmannsthal<br>◯ Waterpan<br>◯ Waterval<br>◯ Waterworks<br>◯ Wattville<br>◯ Wedela<br>◯ Welverdiend<br>◯ West Village<br>◯ West-Driefontein<br>◯ Western Areas Gold Mine<br>◯ Western Deep Levels Mine<br>◯ Westonaria<br>◯ Westonaria NU<br>◯ Winterveld<br>◯ Wolwekrans<br>◯ Zakariyya Park<br>◯ Zenzele<br>◯ Zevenfontein<br>◯ Zithobeni<br>◯ Zwartkop<br>◯ Zwavelpoort
Please specify:	________________________________
Where within Limpopo?	◯ Aganang<br>◯ Ba-Phalaborwa<br>◯ Bela-Bela<br>◯ Blouberg<br>◯ Elias Motsoaledi<br>◯ Fetakgomo<br>◯ Greater Giyani<br>◯ Greater Letaba<br>◯ Greater Marble Hall<br>◯ Greater Tubatse<br>◯ Greater Tzaneen<br>◯ Lepelle-Nkumpi<br>◯ Lephalale<br>◯ Makhado<br>◯ Makhuduthamaga<br>◯ Malamulele<br>◯ Maruleng<br>◯ Modimolle<br>◯ Mogalakwena<br>◯ Molemole<br>◯ Mookgopong<br>◯ Musina<br>◯ Polokwane<br>◯ Thabazimbi<br>◯ Thulamela<br>◯ Other<br>◯ Don't know
Please specify:	________________________________
Where within Western Cape?	________________________________

Where within Eastern Cape?	__________________________________
Where within North West?	__________________________________
Where within KwaZulu Natal?	__________________________________
Where within Northern Cape?	__________________________________
Where within Free State?	__________________________________
(6) Was it rural or urban?	◯ Rural<br>◯ Urban (city or town)<br>◯ Don't know<br>(Rural meaning locations like villages, agricultural areas, or game farms)
(6) When did you start living there? MONTH	◯ January<br>◯ February<br>◯ March<br>◯ April<br>◯ May<br>◯ June<br>◯ July<br>◯ August<br>◯ September<br>◯ October<br>◯ November<br>◯ December<br>◯ Don't know

(6) When did you start living there? YEAR

◯ 2019
◯ 2018
◯ 2017
◯ 2016
◯ 2015
◯ 2014
◯ 2013
◯ 2012
◯ 2011
◯ 2010
◯ 2009
◯ 2008
◯ 2007
◯ 2006
◯ 2005
◯ 2004
◯ 2003
◯ 2002
◯ 2001
◯ 2000
◯ 1999
◯ 1998
◯ 1997
◯ 1996
◯ 1995
◯ 1994
◯ 1993
◯ 1992
◯ 1991
◯ 1990
◯ 1989
◯ 1988
◯ 1987
◯ 1986
◯ 1985
◯ 1984
◯ 1983
◯ 1982
◯ 1981
◯ 1980
◯ Don't Know

(6) What was your main reason for moving to this place of residence?	◯ Job assignment/transfer<br>◯ Retrenchment<br>◯ Better employment opportunities<br>◯ Better accommodation prospects<br>◯ Better educational/training opportunities<br>◯ Closer to school or work<br>◯ Better public/social services<br>◯ Moved with family<br>◯ Joined family or friends<br>◯ Marriage<br>◯ Divorce<br>◯ Death of a family member<br>◯ Family responsibility<br>◯ Attraction to urban life style<br>◯ Evicted from previous residence<br>◯ Not able to pay rent<br>◯ Government resettlement<br>◯ Left to escape violence<br>◯ Other (specify)<br>◯ Dont know
(6) Please specify	______________________________

(6) What was your main formal/informal occupation in the month BEFORE you moved?

◯ None
◯ Farm work
◯ Domestic work
◯ Construction work
◯ Security work
◯ Cleaning work
◯ Small business owner
◯ Mine work
◯ Teacher
◯ Traditional healer
◯ Health sector (formal)
◯ Game farm/game reserve (e.g. ranger)
◯ Driver
◯ Skilled worker (e.g. plumber, mechanic, electrician)
◯ Cook/ chef/ catering
◯ Unskilled worker (e.g. general labourer)
◯ Artisan (e.g. carpenter, wood carver, weaver)
◯ Waiter/ barman
◯ Informal selling
◯ Small business assistant
◯ Clerical and office work
◯ Cattle herder
◯ Sewing, hairdressing, baking, brewing
◯ Police, soldier, fireman
◯ Petrol attendant
◯ Timber, sawmill, poles
◯ Gardening services
◯ Fieldworker - NGO or university
◯ Art, craft, photography, fashion design
◯ Senior administrator, manager, professional
◯ Priest/pastor
◯ Retail/clerk/sales
◯ Student
◯ Other
◯ Don't know

(If unsure of how to classify, select "Other")

(6) Please specify

____

(6) What was your main formal/informal occupation in the month AFTER you moved?	◯ None<br>◯ Farm work<br>◯ Domestic work<br>◯ Construction work<br>◯ Security work<br>◯ Cleaning work<br>◯ Small business owner<br>◯ Mine work<br>◯ Teacher<br>◯ Traditional healer<br>◯ Health sector (formal)<br>◯ Game farm/game reserve (e.g. ranger)<br>◯ Driver<br>◯ Skilled worker (e.g. plumber, mechanic, electrician)<br>◯ Cook/ chef/ catering<br>◯ Unskilled worker (e.g. general labourer)<br>◯ Artisan (e.g. carpenter, wood carver, weaver)<br>◯ Waiter/ barman<br>◯ Informal selling<br>◯ Small business assistant<br>◯ Clerical and office work<br>◯ Cattle herder<br>◯ Sewing, hairdressing, baking, brewing<br>◯ Police, soldier, fireman<br>◯ Petrol attendant<br>◯ Timber, sawmill, poles<br>◯ Gardening services<br>◯ Fieldworker - NGO or university<br>◯ Art, craft, photography, fashion design<br>◯ Senior administrator, manager, professional<br>◯ Priest/pastor<br>◯ Retail/clerk/sales<br>◯ Student<br>◯ Other<br>◯ Don't know<br>(If unsure of how to classify, select "Other")
(6) Please specify	__________________________________
(6) In what type of place did you live?	◯ Commercial farm<br>◯ Game farm/nature reserve<br>◯ Mine<br>◯ Military base<br>◯ City (inner)<br>◯ City (suburb)<br>◯ Town<br>◯ Village (trust land)<br>◯ Informal settlement (city)<br>◯ Informal settlement (rural)<br>◯ Township (urban)<br>◯ Township (rural)<br>◯ Other<br>◯ Don't know
(6) Please specify	__________________________________

(6) What type of dwelling did you live in?	◯ House, brick or concrete structure on a separate stand<br>◯ Traditional dwelling, hut or structure made of traditional materials<br>◯ Flat or apartment in a block of flats/complex<br>◯ Cluster house in a complex<br>◯ Townhouse<br>◯ Semi-detached house not in a complex<br>◯ House, flat or room separate from main dwelling in backyard<br>◯ Informal dwelling or shack in backyard<br>◯ Informal dwelling NOT in backyard, e.g. in informal squatter settlement or on a farm<br>◯ Room or flat which is part of main dwelling or property<br>◯ Caravan or tent<br>◯ Unit in a retirement home or barracks etc.<br>◯ Other<br>◯ Don't know
(6) When did you stop living there? MONTH	◯ January<br>◯ February<br>◯ March<br>◯ April<br>◯ May<br>◯ June<br>◯ July<br>◯ August<br>◯ September<br>◯ October<br>◯ November<br>◯ December<br>◯ Don't know
(6) Please specify	__________________________________
(6) With?whom did you live at this residence?	☐ Parent(s)<br>☐ Spouse/partner/boyfriend/girlfriend<br>☐ Spouse and children<br>☐ Parent(s) in-law<br>☐ Friends<br>☐ Relatives<br>☐ Countrymen<br>☐ Alone<br>☐ Second family<br>☐ Other<br>☐ Don't know
(6) Please specify	__________________________________

(6) When did you stop living there? YEAR

- ◯ 2019
- ◯ 2018
- ◯ 2017
- ◯ 2016
- ◯ 2015
- ◯ 2014
- ◯ 2013
- ◯ 2012
- ◯ 2011
- ◯ 2010
- ◯ 2009
- ◯ 2008
- ◯ 2007
- ◯ 2006
- ◯ 2005
- ◯ 2004
- ◯ 2003
- ◯ 2002
- ◯ 2001
- ◯ 2000
- ◯ 1999
- ◯ 1998
- ◯ 1997
- ◯ 1996
- ◯ 1995
- ◯ 1994
- ◯ 1993
- ◯ 1992
- ◯ 1991
- ◯ 1990
- ◯ 1989
- ◯ 1988
- ◯ 1987
- ◯ 1986
- ◯ 1985
- ◯ 1984
- ◯ 1983
- ◯ 1982
- ◯ 1981
- ◯ 1980
- ◯ Don't Know

When you've completed [q_6_10] residence history loops, select "Save and go to next instrument"

Short Term Absences

____

SHORT TERM ABSENCES (for non-migrants)

In the past 12 months, have you been away from [q_1_5] for more than one month?
- ◯ Yes
- ◯ No
- ◯ Dont know

During your most recent trip away, how long did you spend away from your home village?
- ◯ 1-2 months
- ◯ 2-3 months
- ◯ 3-6 months
- ◯ Dont know

Why were you away?
- ◯ Taking up employment/work
- ◯ Looking for work
- ◯ School/Training
- ◯ To be with spouse/partner
- ◯ Holiday
- ◯ Health reasons
- ◯ Look after/assist family members
- ◯ Family conflict
- ◯ Looking for better housing
- ◯ Escape unfavorable conditions
- ◯ Other
- ◯ Don't know

Please specify ______________________________

Where did you live/stay during this time away from [q_1_5]? What province?
- ◯ Mpumalanga
- ◯ Gauteng
- ◯ Limpopo
- ◯ Free State
- ◯ KwaZulu Natal
- ◯ Eastern Cape
- ◯ Western Cape
- ◯ Northern Cape
- ◯ North West
- ◯ Other

Please specify: ______________________________

Where within Mpumalanga?

◯ Agincourt study site
◯ Barberton
◯ Bushbuckridge
◯ Dwarsloop
◯ Game park Mpumalanga
◯ Gemu / Hazyview
◯ Graskop/ Pilgrim's rest
◯ Kangwane (former homeland)
◯ Kiepersol
◯ Komatipoort
◯ Machadodorp
◯ Malelane
◯ Mapulaneng / Bushbuckridge
◯ Marite / Alexandria
◯ Matsulu
◯ Middleburg
◯ Mkhuhlu
◯ Nelspruit
◯ Pienaar (Nelspruit)
◯ Sabie
◯ Thulamahashe
◯ White River
◯ Witbank
◯ Zwelitsha
◯ Other Mpumalanga
◯ Don't know

Please specify: __________________________________

Where within Gauteng

◯ Akasia
◯ Alberton
◯ Alexandra
◯ Atteridgeville
◯ Babelegi
◯ Bapsfontein
◯ Bashewa
◯ Baviaanspoort
◯ Bekkersdal
◯ Benoni
◯ Bhongweni
◯ Blue Hills
◯ Boipatong
◯ Boksburg
◯ Bon Accord
◯ Bonamanzi Marina and Country Club
◯ Bophelong
◯ Boschkop
◯ Bosplaas Mathabe
◯ Bothasgeluk
◯ Brakpan
◯ Breswol
◯ Bronkhorstspruit
◯ Bultfontein
◯ Carletonville
◯ Centurion
◯ Chartwell
◯ Cheetah Park
◯ Chief A Luthuli Park
◯ City of Johannesburg NU
◯ Clayville
◯ Cullinan
◯ Dainfern
◯ Daveyton
◯ Deelkraal Gold Mine
◯ Derdepoort
◯ Devon A
◯ Devon B
◯ Diepsloot
◯ Dilopye
◯ Donkerhoek
◯ Doornfontein
◯ Doornkraal
◯ Downbern
◯ Drie Ziek
◯ Duduza
◯ Dukathole
◯ East Driefontein
◯ East Village
◯ Ebony Park
◯ Edenvale
◯ Eersterust
◯ Ekangala
◯ Ekurhuleni NU
◯ Elandsfontein
◯ Elandsrand
◯ Elandsridge
◯ Elsburg Gold Mine
◯ Emfuleni NU
◯ Endicott
◯ Ennerdale
◯ Etwatwa
◯ Evaton
◯ Farmall
◯ Fochville
◯ Ga-Rankuwa
◯ Geluksdal
◯ Germiston
◯ Glen Harvie

◯ Golden Gardens
◯ Goudvlakte West
◯ Green Park
◯ Grootfontein
◯ Grootvlei
◯ Haakdoornboom
◯ Hallgate
◯ Hammanskraal
◯ Harry Gwala
◯ Hartebeesfontein
◯ Hebron
◯ Heidelberg
◯ Hillshaven
◯ Holfontein
◯ Impumelelo
◯ Itsoseng
◯ Ivory Park
◯ Johandeo
◯ Johannesburg
◯ Kaalfontein
◯ Kagiso
◯ Kameeldrift
◯ Kameelfontein
◯ Kameelkraal
◯ Kanana
◯ Kanana Park
◯ Katlehong
◯ Other
◯ Keinfontein
◯ Kekana Garden
◯ Kempton Park
◯ Khutsong
◯ Kleinfontein
◯ Kokosi
◯ Krugersdorp
◯ Kungwini Part 2
◯ Kwa-Thema
◯ Lakeside
◯ Langaville
◯ Lanseria
◯ Laudium
◯ Lawley
◯ Leeufontein
◯ Leeuport
◯ Lehae
◯ Lenasia
◯ Lenasia South
◯ Lenz
◯ Lesedi NU
◯ Letsatsing
◯ Lindelani Village
◯ Lucky 7
◯ Mabopane
◯ Magaliesburg
◯ Majaneng
◯ Makanyaneng
◯ Malatjie
◯ Mamello
◯ Mamelodi
◯ Mandela Village
◯ Marokolong
◯ Mashemong
◯ Mayibuye
◯ Merafong City NU
◯ Meyerton
◯ Midrand
◯ Midvaal NU
◯ Millgate Farm
◯ Mogale City NU
◯ Mohlakeng
◯ Mooiplaas

◯ Munsieville
◯ Nellmapius
◯ New Eersterus
◯ Nigel
◯ Nooitgecht
◯ Nufcor
◯ Oberholzer
◯ Olievenhoutbos
◯ Onverwacht
◯ Orange Farm
◯ Orient Hills
◯ Pebble Rock Golf Village
◯ Phomolong
◯ Poortjie
◯ Pretoria
◯ Rabie Ridge
◯ Ramotse
◯ Randburg
◯ Randfontein
◯ Randfontein NU
◯ Randvaal
◯ Ratanda
◯ Rayton
◯ Refilwe
◯ Rethabiseng
◯ Rietfontein
◯ Rietvallei
◯ Roodeplaat
◯ Roodepoort
◯ Sable Hills Waterfront Estate
◯ Sandton
◯ Saulsville
◯ Seberuberung
◯ Sebokeng
◯ Sharpeville
◯ Simunye
◯ Sonstraal
◯ Soshanguve
◯ Southdene
◯ Soutpan
◯ Soweto
◯ Spaarwater
◯ Springs
◯ Stinkwater
◯ Stretford
◯ Suurman
◯ Temba
◯ Tembisa
◯ The Carousel Casino and Entertainment World
◯ The Hill
◯ The Village
◯ Thembisile
◯ Thinasonke
◯ Tierpoort
◯ Tokoza
◯ Tsakane
◯ Tsebe
◯ Tshepiso
◯ Tshepisong
◯ Tshwane NU
◯ Tweedracht
◯ Tweefontein
◯ Vaal Marina
◯ Vaal Oewer
◯ Vaalbank
◯ Vanderbijlpark
◯ Vastfontein
◯ Venterspost
◯ Vereeniging
◯ Viskuil
◯ Vlakfontein

◯ Vosloorus
◯ Wageenbietjieskop
◯ Walkerville
◯ Wallis Haven
◯ Wallmannsthal
◯ Waterpan
◯ Waterval
◯ Waterworks
◯ Wattville
◯ Wedela
◯ Welverdiend
◯ West Village
◯ West-Driefontein
◯ Western Areas Gold Mine
◯ Western Deep Levels Mine
◯ Westonaria
◯ Westonaria NU
◯ Winterveld
◯ Wolwekrans
◯ Zakariyya Park
◯ Zenzele
◯ Zevenfontein
◯ Zithobeni
◯ Zwartkop
◯ Zwavelpoort

Please specify:

____

Where within Limpopo?

◯ Aganang
◯ Ba-Phalaborwa
◯ Bela-Bela
◯ Blouberg
◯ Elias Motsoaledi
◯ Fetakgomo
◯ Greater Giyani
◯ Greater Letaba
◯ Greater Marble Hall
◯ Greater Tubatse
◯ Greater Tzaneen
◯ Lepelle-Nkumpi
◯ Lephalale
◯ Makhado
◯ Makhuduthamaga
◯ Malamulele
◯ Maruleng
◯ Modimolle
◯ Mogalakwena
◯ Molemole
◯ Mookgopong
◯ Musina
◯ Polokwane
◯ Thabazimbi
◯ Thulamela
◯ Other
◯ Don't know

Please specify:

____

Where within Western Cape?

____

Where within Eastern Cape?	__________________________
Where within North West?	__________________________
Where within KwaZulu Natal?	__________________________
Where within Northern Cape?	__________________________
Where within Free State?	__________________________
In what type of place did you live during this time?	◯ Commercial farm<br>◯ Game farm/nature reserve<br>◯ Mine<br>◯ Military base<br>◯ City (inner)<br>◯ City (suburb)<br>◯ Town<br>◯ Village (trust land)<br>◯ Informal settlement (city)<br>◯ Informal settlement (rural)<br>◯ Township (urban)<br>◯ Township (rural)<br>◯ Other<br>◯ Don't know
Please specify	__________________________
Was it rural or urban	◯ Rural<br>◯ Urban (city or town)<br>◯ Don't know<br>(Rural meaning locations like villages, agricultural areas, or game farms)
What type of dwelling did you stay in during this time?	◯ House, brick or concrete structure on a separate stand<br>◯ Traditional dwelling, hut or structure made of traditional materials<br>◯ Flat or apartment in a block of flats/complex<br>◯ Cluster house in a complex<br>◯ Townhouse<br>◯ Semi-detached house not in a complex<br>◯ House, flat or room separate from main dwelling in backyard<br>◯ Informal dwelling or shack in backyard<br>◯ Informal dwelling NOT in backyard, e.g. in informal squatter settlement or on a farm<br>◯ Room or flat which is part of main dwelling or property<br>◯ Caravan or tent<br>◯ Unit in a retirement home or barracks etc.<br>◯ Other<br>◯ Don't know

Please specify

____

Mark as "Complete" and select "Save and go to next instrument" when complete

Remittances

REMITTANCES

Do you typically send money or goods back to [q_1_5] while you are away?	◯ Yes<br>◯ No<br>◯ Don't know
During the past 12 months, who is the main person that you normally send your money to?	◯ Mother<br>◯ Father<br>◯ Spouse/Partner<br>◯ Son<br>◯ Daughter<br>◯ Maternal Grandmother<br>◯ Maternal Grandfather<br>◯ Paternal Grandmother<br>◯ Paternal Grandfather<br>◯ Maternal Aunt<br>◯ Maternal Uncle<br>◯ Paternal Aunt<br>◯ Paternal Uncle<br>◯ Other (specify)<br>◯ Dont know
Please specify	__________________________
In the past 12 months, what was the value of the money or goods you sent back?	◯ Less than R199<br>◯ R200-R499<br>◯ R500-R999<br>◯ R1 000-R1 499<br>◯ R1 500-R2 499<br>◯ More than R2 500<br>◯ Don't know
In the past 12 months, did you personally buy any of the following for the household in [q_1_5]?	☐ Food<br>☐ Clothing<br>☐ Cell phone<br>☐ Airtime<br>☐ Household appliances (e.g. fridge, microwave, etc.)<br>☐ Livestock<br>☐ Furniture<br>☐ Building materials<br>☐ Vehicle<br>☐ Other<br>☐ Don't know
Please specify	__________________________

In the past 12 months, when did you typically visit [q_1_5]?	◯ I do not visit home<br>◯ Most weekends<br>◯ Month ends<br>◯ Month ends plus holiday(s)<br>◯ One long period/holiday<br>◯ Two periods/holidays<br>◯ School holidays<br>◯ Christmas and Easter<br>◯ Christmas only<br>◯ Irregularly<br>◯ Other<br>◯ Don't know
Please specify	__________________________________
When did you last visit [q_1_5]?	◯ Never<br>◯ In the last month<br>◯ 2-3 months ago<br>◯ 4-5 months ago<br>◯ 6 months to 1 year ago<br>◯ 1 to 2 years ago<br>◯ More than 2 years ago<br>◯ Don't know
When was the most recent communication between you and somebody in the household in [q_1_5]?	◯ Within the last week<br>◯ Within the last month (but more than a week)<br>◯ 2-3 months ago<br>◯ 4-5 months ago<br>◯ 6 months to 1 year ago<br>◯ 1 to 2 years ago<br>◯ More than 2 years ago<br>◯ Never<br>◯ Don't know
What mode was used for this recent communication?	◯ In person at home<br>◯ In person at work<br>◯ Telephone (call)<br>◯ Telephone (text/sms)<br>◯ Verbal message<br>◯ Email<br>◯ Social media (Facebook, Twitter, etc.)<br>◯ WhatsApp<br>◯ Written message/letter<br>◯ Other<br>◯ Don't know
Please specify	__________________________________

Mark as "Complete" and select "Save and go to next instrument" when complete

Social Capital

SOCIAL CAPITAL

Do you have a family member(s) or relative(s) living near you?

◯ Yes ◯ No ◯ Dont know

Did you personally get any assistance (money, food, childcare, or other material help) from your family member in the last month?

◯ Yes
◯ No
◯ Dont know

What type of assistance did you get?

☐ Food
☐ Money
☐ Other household groceries
☐ Uniform
☐ Clothing, excluding uniform
☐ Child care
☐ Transport
☐ Care when sick
☐ Care of the elderly or sick
☐ Fuel (e.g. wood, petrol, paraffin)
☐ Help with household chores
☐ Guidance/advice
☐ Assistance finding work/networking
☐ Other
☐ Don't know

Please specify

____

Do you have a friend(s) living near you?

◯ Yes ◯ No ◯ Dont know

Did you personally get any assistance (money, food, childcare, or other material help) from your friend in the last month?

◯ Yes
◯ No
◯ Dont know

What type of assistance did you get?

☐ Food
☐ Money
☐ Other household groceries
☐ Uniform
☐ Clothing, excluding uniform
☐ Child care
☐ Transport
☐ Care when sick
☐ Care of the elderly or sick
☐ Fuel (e.g. wood, petrol, paraffin)
☐ Help with household chores
☐ Guidance/advice
☐ Assistance finding work/networking
☐ Other
☐ Don't know

Please specify

____

Mark as "Complete" and select "Save and go to next instrument" when complete

General Health

GENERAL HEALTH

In general, how would you rate your health today?	◯ Very poor<br>◯ Poor<br>◯ Average<br>◯ Good<br>◯ Very good
Compared to this time last year, would you say your health is:	◯ Much worse<br>◯ Worse<br>◯ Same<br>◯ Better<br>◯ Much better
Compared to most people of your age and gender in your village/neighbourhood, would you say your health is generally:	◯ Much worse<br>◯ Worse<br>◯ Same<br>◯ Better<br>◯ Much better<br>(Here we mean where the respondent is CURRENTLY LIVING.)
Overall in the last 30 days, how much difficulty did you have with work or household activities?	◯ None<br>◯ Mild<br>◯ Moderate<br>◯ Severe<br>◯ Extreme/Cannot do<br>(e.g. your job, cooking, cleaning, toileting, etc)
In the past year, were you ever sick so that you couldn't work or attend school?	◯ Yes<br>◯ No<br>◯ Dont know
For how long?	◯ A day<br>◯ 2-3 days<br>◯ 4-7 days<br>◯ 1-2 weeks<br>◯ More than 2 weeks<br>◯ Dont know
In the past year, have you been sick continuously for longer than a month?	◯ Yes<br>◯ No<br>◯ Dont know
Have you ever been told by a doctor, nurse, or other medical professional that you have a chronic illness?	◯ Yes<br>◯ No<br>◯ Don't know<br>(A chronic illness is a disease or illness that lasts for 6 months or more)

Which of the following chronic illnesses have you been diagnosed with?	☐ High blood pressure or hypertension<br>☐ Diabetes<br>☐ High cholesterol<br>☐ HIV<br>☐ TB<br>☐ Asthma<br>☐ Chronic Obstructive Pulmonary (COPD)/Emphysema Disease<br>☐ Depression or other mental health condition<br>☐ Stroke<br>☐ Other<br>☐ Dont know
Please specify	__________________________________
Are you on medication for a chronic illness (medication repeated for more than 3 months)?	◯ Yes<br>◯ No<br>◯ Dont know
What are the conditions for which you are currently using prescribed medication?	☐ High blood pressure or hypertension<br>☐ Diabetes<br>☐ High cholesterol<br>☐ HIV<br>☐ TB<br>☐ Asthma<br>☐ Chronic Obstructive Pulmonary (COPD)/Emphysema Disease<br>☐ Depression or other mental health condition<br>☐ Stroke<br>☐ Other<br>☐ Dont know<br>(A respondent may have been diagnosed with a chronic illness but is not taking medication for it.)
Please specify	__________________________________
When were you diagnosed with high blood pressure or hypertension? (MONTH)	◯ January<br>◯ February<br>◯ March<br>◯ April<br>◯ May<br>◯ June<br>◯ July<br>◯ August<br>◯ September<br>◯ October<br>◯ November<br>◯ December<br>◯ Don't know

When were you diagnosed with high blood pressure or hypertension? (YEAR)	◯ 2019<br>◯ 2018<br>◯ 2017<br>◯ 2016<br>◯ 2015<br>◯ 2014<br>◯ 2013<br>◯ 2012<br>◯ 2011<br>◯ 2010<br>◯ 2009<br>◯ 2008<br>◯ 2007<br>◯ 2006<br>◯ 2005<br>◯ 2004<br>◯ 2003<br>◯ 2002<br>◯ 2001<br>◯ 2000<br>◯ 1999<br>◯ 1998<br>◯ 1997<br>◯ 1996<br>◯ 1995<br>◯ 1994<br>◯ 1993<br>◯ 1992<br>◯ 1991<br>◯ 1990<br>◯ 1989<br>◯ 1988<br>◯ 1987<br>◯ 1986<br>◯ 1985<br>◯ 1984<br>◯ 1983<br>◯ 1982<br>◯ 1981<br>◯ 1980
Where were you living when you were diagnosed with high blood pressure or hypertension?	◯ Same village/neighbourhood in [q_1_5]<br>◯ Village/neighbourhood nearby [q_1_5]<br>◯ Same village/neighbourhood in [current_residence]<br>◯ Village/neighbourhood nearby [current_residence]<br>◯ Other<br>◯ Don't know<br>(If the answer categories repeat (i.e. origin location and current residence are the same), select the answer from the first two options (unless other or don't know))
Please specify	__________________________________
In the last 12 months have you sought and/or needed treatment for your high blood pressure or hypertension?	◯ Yes<br>◯ No<br>◯ Dont know
In the last 12 months have you experienced a lapse or difficulty in accessing your treatment for high blood pressure or hypertension?	◯ Yes<br>◯ No<br>◯ Dont know

Why was there a lapse or difficulty in accessing your treatment for high blood pressure or hypertension?	◯ Could not afford the cost of the visit<br>◯ No transport available<br>◯ Could not afford the cost of transport<br>◯ Treated poorly during a previous visit<br>◯ Could not take time off of work; busy<br>◯ Drugs or equipment seemingly inadequate<br>◯ Health providers skills seemingly inadequate<br>◯ Did not know where to go<br>◯ Tried but was denied health care<br>◯ Did not think you were sick enough<br>◯ Difficulties due to moving/relocating<br>◯ Could not find health care facilities or doctor<br>◯ Other<br>◯ Don't know
Please specify	________________________
When were you diagnosed with diabetes (MONTH)	◯ January<br>◯ February<br>◯ March<br>◯ April<br>◯ May<br>◯ June<br>◯ July<br>◯ August<br>◯ September<br>◯ October<br>◯ November<br>◯ December<br>◯ Don't know

When were you diagnosed with diabetes? (YEAR)	◯ 2019<br>◯ 2018<br>◯ 2017<br>◯ 2016<br>◯ 2015<br>◯ 2014<br>◯ 2013<br>◯ 2012<br>◯ 2011<br>◯ 2010<br>◯ 2009<br>◯ 2008<br>◯ 2007<br>◯ 2006<br>◯ 2005<br>◯ 2004<br>◯ 2003<br>◯ 2002<br>◯ 2001<br>◯ 2000<br>◯ 1999<br>◯ 1998<br>◯ 1997<br>◯ 1996<br>◯ 1995<br>◯ 1994<br>◯ 1993<br>◯ 1992<br>◯ 1991<br>◯ 1990<br>◯ 1989<br>◯ 1988<br>◯ 1987<br>◯ 1986<br>◯ 1985<br>◯ 1984<br>◯ 1983<br>◯ 1982<br>◯ 1981<br>◯ 1980
Where were you living when you were diagnosed with diabetes?	◯ Same village/neighbourhood in [q_1_5]<br>◯ Village/neighbourhood nearby [q_1_5]<br>◯ Same village/neighbourhood in [current_residence]<br>◯ Village/neighbourhood nearby [current_residence]<br>◯ Other<br>◯ Don't know<br>(If the answer categories repeat (i.e. origin location and current residence are the same), select the answer from the first two options (unless other or don't know))
Please specify	__________________________________
In the last 12 months have you sought and/or needed treatment for your diabetes?	◯ Yes<br>◯ No<br>◯ Dont know
In the last 12 months have you experienced a lapse or difficulty in accessing your treatment for diabetes?	◯ Yes<br>◯ No<br>◯ Dont know

Why was there a lapse or difficulty in accessing your treatment for diabetes?	◯ Could not afford the cost of the visit<br>◯ No transport available<br>◯ Could not afford the cost of transport<br>◯ Treated poorly during a previous visit<br>◯ Could not take time off of work; busy<br>◯ Drugs or equipment seemingly inadequate<br>◯ Health providers skills seemingly inadequate<br>◯ Did not know where to go<br>◯ Tried but was denied health care<br>◯ Did not think you were sick enough<br>◯ Difficulties due to moving/relocating<br>◯ Could not find health care facilities or doctor<br>◯ Other<br>◯ Don't know
Please specify	______________________________
When were you told by a doctor, nurse, or medical professional that you had a stroke? (MONTH)	◯ January<br>◯ February<br>◯ March<br>◯ April<br>◯ May<br>◯ June<br>◯ July<br>◯ August<br>◯ September<br>◯ October<br>◯ November<br>◯ December<br>◯ Don't know

When were you told by a doctor, nurse, or medical professional that you had a stroke? (YEAR)	◯ 2019<br>◯ 2018<br>◯ 2017<br>◯ 2016<br>◯ 2015<br>◯ 2014<br>◯ 2013<br>◯ 2012<br>◯ 2011<br>◯ 2010<br>◯ 2009<br>◯ 2008<br>◯ 2007<br>◯ 2006<br>◯ 2005<br>◯ 2004<br>◯ 2003<br>◯ 2002<br>◯ 2001<br>◯ 2000<br>◯ 1999<br>◯ 1998<br>◯ 1997<br>◯ 1996<br>◯ 1995<br>◯ 1994<br>◯ 1993<br>◯ 1992<br>◯ 1991<br>◯ 1990<br>◯ 1989<br>◯ 1988<br>◯ 1987<br>◯ 1986<br>◯ 1985<br>◯ 1984<br>◯ 1983<br>◯ 1982<br>◯ 1981<br>◯ 1980
Where were you living when you were told by a doctor, nurse, or medical professional that you had a stroke?	◯ Same village/neighbourhood in [q_1_5]<br>◯ Village/neighbourhood nearby [q_1_5]<br>◯ Same village/neighbourhood in [current_residence]<br>◯ Village/neighbourhood nearby [current_residence]<br>◯ Other<br>◯ Don't know<br>(If the answer categories repeat (i.e. origin location and current residence are the same), select the answer from the first two options (unless other or don't know))
Please specify	__________________________________
In the last 12 months have you sought and/or needed treatment for your stroke?	◯ Yes<br>◯ No<br>◯ Dont know
In the last 12 months have you experienced a lapse or difficulty in accessing your treatment for your stroke?	◯ Yes<br>◯ No<br>◯ Dont know

Why was there a lapse or difficulty in accessing your treatment for your stroke?	◯ Could not afford the cost of the visit<br>◯ No transport available<br>◯ Could not afford the cost of transport<br>◯ Treated poorly during a previous visit<br>◯ Could not take time off of work; busy<br>◯ Drugs or equipment seemingly inadequate<br>◯ Health providers skills seemingly inadequate<br>◯ Did not know where to go<br>◯ Tried but was denied health care<br>◯ Did not think you were sick enough<br>◯ Difficulties due to moving/relocating<br>◯ Could not find health care facilities or doctor<br>◯ Other<br>◯ Don't know
Please specify	__________________________________
When were you diagnosed with TB (MONTH)	◯ January<br>◯ February<br>◯ March<br>◯ April<br>◯ May<br>◯ June<br>◯ July<br>◯ August<br>◯ September<br>◯ October<br>◯ November<br>◯ December<br>◯ Don't know

When were you diagnosed with TB? (YEAR)	◯ 2019<br>◯ 2018<br>◯ 2017<br>◯ 2016<br>◯ 2015<br>◯ 2014<br>◯ 2013<br>◯ 2012<br>◯ 2011<br>◯ 2010<br>◯ 2009<br>◯ 2008<br>◯ 2007<br>◯ 2006<br>◯ 2005<br>◯ 2004<br>◯ 2003<br>◯ 2002<br>◯ 2001<br>◯ 2000<br>◯ 1999<br>◯ 1998<br>◯ 1997<br>◯ 1996<br>◯ 1995<br>◯ 1994<br>◯ 1993<br>◯ 1992<br>◯ 1991<br>◯ 1990<br>◯ 1989<br>◯ 1988<br>◯ 1987<br>◯ 1986<br>◯ 1985<br>◯ 1984<br>◯ 1983<br>◯ 1982<br>◯ 1981<br>◯ 1980
Where were you living when you were diagnosed with TB?	◯ Same village/neighbourhood in [q_1_5]<br>◯ Village/neighbourhood nearby [q_1_5]<br>◯ Same village/neighbourhood in [current_residence]<br>◯ Village/neighbourhood nearby [current_residence]<br>◯ Other<br>◯ Don't know<br>(If the answer categories repeat (i.e. origin location and current residence are the same), select the answer from the first two options (unless other or don't know))
Please specify	__________________________________
In the last 12 months have you sought and/or needed treatment for your TB?	◯ Yes<br>◯ No<br>◯ Dont know
In the last 12 months have you experienced a lapse or difficulty in accessing your treatment for your TB?	◯ Yes<br>◯ No<br>◯ Dont know

Why was there a lapse or difficulty in accessing your treatment for your TB?	◯ Could not afford the cost of the visit<br>◯ No transport available<br>◯ Could not afford the cost of transport<br>◯ Treated poorly during a previous visit<br>◯ Could not take time off of work; busy<br>◯ Drugs or equipment seemingly inadequate<br>◯ Health providers skills seemingly inadequate<br>◯ Did not know where to go<br>◯ Tried but was denied health care<br>◯ Did not think you were sick enough<br>◯ Difficulties due to moving/relocating<br>◯ Could not find health care facilities or doctor<br>◯ Other<br>◯ Don't know
Please specify	______________________________
When were you diagnosed with HIV (MONTH)	◯ January<br>◯ February<br>◯ March<br>◯ April<br>◯ May<br>◯ June<br>◯ July<br>◯ August<br>◯ September<br>◯ October<br>◯ November<br>◯ December<br>◯ Don't know

When were you diagnosed with HIV? (YEAR)	◯ 2019<br>◯ 2018<br>◯ 2017<br>◯ 2016<br>◯ 2015<br>◯ 2014<br>◯ 2013<br>◯ 2012<br>◯ 2011<br>◯ 2010<br>◯ 2009<br>◯ 2008<br>◯ 2007<br>◯ 2006<br>◯ 2005<br>◯ 2004<br>◯ 2003<br>◯ 2002<br>◯ 2001<br>◯ 2000<br>◯ 1999<br>◯ 1998<br>◯ 1997<br>◯ 1996<br>◯ 1995<br>◯ 1994<br>◯ 1993<br>◯ 1992<br>◯ 1991<br>◯ 1990<br>◯ 1989<br>◯ 1988<br>◯ 1987<br>◯ 1986<br>◯ 1985<br>◯ 1984<br>◯ 1983<br>◯ 1982<br>◯ 1981<br>◯ 1980
Where were you living when you were diagnosed with HIV?	◯ Same village/neighbourhood in [q_1_5]<br>◯ Village/neighbourhood nearby [q_1_5]<br>◯ Same village/neighbourhood in [current_residence]<br>◯ Village/neighbourhood nearby [current_residence]<br>◯ Other<br>◯ Don't know<br>(If the answer categories repeat (i.e. origin location and current residence are the same), select the answer from the first two options (unless other or don't know))
Please specify	__________________________________
In the last 12 months have you sought treatment for your HIV?	◯ Yes<br>◯ No<br>◯ Dont know
In the last 12 months have you experienced a lapse or difficulty in accessing your treatment for HIV?	◯ Yes<br>◯ No<br>◯ Dont know

Why was there a lapse or difficulty in accessing your treatment for your HIV?	◯ Could not afford the cost of the visit<br>◯ No transport available<br>◯ Could not afford the cost of transport<br>◯ Treated poorly during a previous visit<br>◯ Could not take time off of work; busy<br>◯ Drugs or equipment seemingly inadequate<br>◯ Health providers skills seemingly inadequate<br>◯ Did not know where to go<br>◯ Tried but was denied health care<br>◯ Did not think you were sick enough<br>◯ Difficulties due to moving/relocating<br>◯ Could not find health care facilities or doctor<br>◯ Other<br>◯ Don't know
Please specify	__________________________________

Mark as "Complete" and select "Save and go to next instrument" when complete

Healthcare Access

HEALTHCARE ACCESS

I would now like to ask you about your use of the following health services in the last 12 months.

In the last 12 months have you used health services in or nearby [q_1_5]?
- ◯ Yes
- ◯ No
- ◯ Dont know

In the last 12 months, how often have you used the following health services in [q_1_5]?

	0 times	Once	2-4 times	5-7 times	8-12 times	More than 12 times
Government hospital	◯	◯	◯	◯	◯	◯
Government clinic	◯	◯	◯	◯	◯	◯
Private hospital, clinic, or doctor	◯	◯	◯	◯	◯	◯
Traditional healer	◯	◯	◯	◯	◯	◯
Prophet, religious, or spiritual healer	◯	◯	◯	◯	◯	◯

	Within walking distance (less than an hour)	Within walking distance (more than an hour)	Within 30 minutes by taxis/car/bus	30-60 minutes by taxis/car/bus	More than 60 minutes by taxis/car/bus	Dont know
How far away was the government hospital you visited?	◯	◯	◯	◯	◯	◯
How far away was the government clinic you visited?	◯	◯	◯	◯	◯	◯
How far away was the private hospital, clinic, or doctor you visited?	◯	◯	◯	◯	◯	◯
How far away was the traditional healer you visited?	◯	◯	◯	◯	◯	◯
How far away was the prophet, religious, or spiritual healer you visited?	◯	◯	◯	◯	◯	◯

In the last 12 months have you used health services in or nearby [current_residence]?
- ◯ Yes
- ◯ No
- ◯ Don't know

In the last 12 months, how often have you used the following health services near [current_residence]?

	0 times	Once	2-4 times	5-7 times	8-12 times	More than 12 times
Government hospital	◯	◯	◯	◯	◯	◯
Government clinic	◯	◯	◯	◯	◯	◯
Private hospital, clinic, or doctor	◯	◯	◯	◯	◯	◯
Traditional healer	◯	◯	◯	◯	◯	◯
Prophet, religious, or spiritual healer	◯	◯	◯	◯	◯	◯

	Within walking distance (less than an hour)	Within walking distance (more than an hour)	Within 30 minutes by taxis/car/bus	30-60 minutes by taxis/car/bus	More than 60 minutes by taxis/car/bus	Dont know
How far away was the government hospital you visited?	◯	◯	◯	◯	◯	◯
How far away was the government clinic you visited?	◯	◯	◯	◯	◯	◯
How far away was the private hospital, clinic, or doctor you visited?	◯	◯	◯	◯	◯	◯
How far away was the traditional healer you visited?	◯	◯	◯	◯	◯	◯
How far away was the prophet, religious, or spiritual healer you visited?	◯	◯	◯	◯	◯	◯

Mark as "Complete" and select "Save and go to next instrument" when complete

Food Security and Diet

FOOD SECURITY & DIET

In an average week how often do you eat a meal from a fast-food or take-away place such as KFC, Steer's, Wimpy's, Spur, etc.	◯ Never<br>◯ Less than once a week<br>◯ 1-2 times a week<br>◯ 3-4 times a week<br>◯ 5 or more times a week
Have there been any days in the last three months when your household experienced a shortage of food to eat because there was not enough money to buy food?	◯ Yes<br>◯ No<br>◯ Dont know
How often in the last three months did your household not have enough to eat because there was not enough money to buy food?	◯ Never<br>◯ Rarely<br>◯ Sometimes<br>◯ Often<br>◯ Very often
Have there been any days in the last year when your household experienced a shortage of food to eat because there was not enough money to buy food?	◯ Yes<br>◯ No<br>◯ Dont know

Now I want to ask you about how often you eat or drink certain things. Please tell me whether you eat or drink the following: Never, 1-3 times per week, 4-6 times per week, Once a day, 2 times a day, 3 or more times a day

	Never	Once or twice a month	1-3 times per week	4-6 times per week	Once a day	2 times a day	3 or more times a day
Chicken (any)	◯	◯	◯	◯	◯	◯	◯
Red meat (any)	◯	◯	◯	◯	◯	◯	◯
Processed meat (polony, ham, viennas, sausages, bacon, meat pies etc.)	◯	◯	◯	◯	◯	◯	◯
Offal and traditional meats (liver, tripe etc.)	◯	◯	◯	◯	◯	◯	◯
Fish (fresh/tinned)	◯	◯	◯	◯	◯	◯	◯
Eggs (any type)	◯	◯	◯	◯	◯	◯	◯
Milk (including in tea, coffee, cereal etc.)	◯	◯	◯	◯	◯	◯	◯
Non-dairy creamer/Cremora	◯	◯	◯	◯	◯	◯	◯
Yoghurt/maas	◯	◯	◯	◯	◯	◯	◯
Cheese and cottage cheese	◯	◯	◯	◯	◯	◯	◯

Legumes (baked beans, lentils, tinyawa, etc.)	○	○	○	○	○	○	○
Peanuts and nuts (including peanut butter)	○	○	○	○	○	○	○
White and traditional bread	○	○	○	○	○	○	○
Brown and wholemeal bread	○	○	○	○	○	○	○
Breakfast cereals	○	○	○	○	○	○	○
Cooked porridge (pap, maize, sorghum, oat)	○	○	○	○	○	○	○
Fat cakes, samosas and pizza	○	○	○	○	○	○	○
Margarine (hard/soft)	○	○	○	○	○	○	○
Green vegetables	○	○	○	○	○	○	○
Salad vegetables	○	○	○	○	○	○	○
Other vegetables	○	○	○	○	○	○	○
Boiled potatoes	○	○	○	○	○	○	○
Roast potatoes and chips	○	○	○	○	○	○	○
Citrus fruit (naartjies, oranges, grapefruit, lemons)	○	○	○	○	○	○	○
Other fruit	○	○	○	○	○	○	○
Fruit juices	○	○	○	○	○	○	○
Rice and pasta	○	○	○	○	○	○	○
Samp and mielie rice	○	○	○	○	○	○	○
Cakes and biscuits	○	○	○	○	○	○	○
Puddings (including ice-cream, custard etc.)	○	○	○	○	○	○	○
Cooking fats and salad oils	○	○	○	○	○	○	○
Sweets and chocolate	○	○	○	○	○	○	○
Crisps, nik naks, etc	○	○	○	○	○	○	○
Added sugar (teaspoons; tea, coffee, cereal etc.)	○	○	○	○	○	○	○
Tea and coffee	○	○	○	○	○	○	○
High-energy soft drinks (including cool drinks, fizzy drinks, squashes etc.)	○	○	○	○	○	○	○
Diet soft drinks	○	○	○	○	○	○	○
Sauces (mayonnaise, tomato sauce, chutney, stock, etc.)	○	○	○	○	○	○	○
Alcoholic beverages (beer, wine, spirits, fermented cider, traditional beer, etc)	○	○	○	○	○	○	○

Mark as "Complete" and select "Save and go to next instrument" when complete

Tobacco and Alcohol Use

TOBACCO AND ALCOHOL USE

Now I am going to ask you some questions about various health behaviours. This includes things like smoking, drinking alcohol, and physical activity. Lets start with tobacco.

Have you ever smoked tobacco regularly?
- ◯ No, never
- ◯ Yes, daily
- ◯ Yes, less than daily
- ◯ Yes, but not now
- ◯ Don't know

Do you currently smoke/chew/use (you will use if you have the possibility) any tobacco products, such as cigarettes, cigars, pipes, chewing tobacco, or snuff?
- ◯ Yes
- ◯ No
- ◯ Dont know

How often do you smoke or use any tobacco products?
- ◯ Never in the past month
- ◯ 1-3 days per month
- ◯ 1-4 days per week
- ◯ 5-6 days per week
- ◯ Daily
- ◯ Don't know

How old were you when you first started smoking?
- ◯ < 10 yrs old
- ◯ 10-13
- ◯ 14-17
- ◯ 18-21
- ◯ 22-25
- ◯ 26-30
- ◯ 31-35
- ◯ > 35 yrs old
- ◯ Don't know

On average, how many cigarettes, pipes of tobacco, cigars, cheroots, etc do you smoke each day?
- ◯ 0
- ◯ 1-2
- ◯ 3-4
- ◯ 5-6
- ◯ 7 or more

When did you stop smoking? MONTH
- ◯ January
- ◯ February
- ◯ March
- ◯ April
- ◯ May
- ◯ June
- ◯ July
- ◯ August
- ◯ September
- ◯ October
- ◯ November
- ◯ December
- ◯ Don't know

When did you stop smoking? YEAR	◯ 2019<br>◯ 2018<br>◯ 2017<br>◯ 2016<br>◯ 2015<br>◯ 2014<br>◯ 2013<br>◯ 2012<br>◯ 2011<br>◯ 2010<br>◯ 2009<br>◯ 2008<br>◯ 2007<br>◯ 2006<br>◯ 2005<br>◯ 2004<br>◯ 2003<br>◯ 2002<br>◯ 2001<br>◯ 2000<br>◯ 1999<br>◯ 1998<br>◯ 1997<br>◯ 1996<br>◯ 1995<br>◯ 1994<br>◯ 1993<br>◯ 1992<br>◯ 1991<br>◯ 1990<br>◯ 1989<br>◯ 1988<br>◯ 1987<br>◯ 1986<br>◯ 1985<br>◯ 1984<br>◯ 1983<br>◯ 1982<br>◯ 1981<br>◯ 1980
The next questions ask about the consumption of alcohol.	
Have you consumed an alcoholic drink such as beer, wine, spirits, fermented cider, or traditional beer in the last 12 months?	◯ Yes<br>◯ No<br>◯ Dont know
How often do you have at least one alcoholic drink?	◯ Never<br>◯ Less than once a month<br>◯ 1-3 days a month<br>◯ 1-4 days a week<br>◯ 5-6 days a week<br>◯ Daily<br>◯ Don't know<br>(One drink is equal to 350ml beer, 150ml of wine, or a shot of spirits.)

On days you drink, how many alcoholic drinks do you have?	◯ 1-2 drinks<br>◯ 3-4 drinks<br>◯ 5-6 drinks<br>◯ 7-10 drinks<br>◯ More than 10 drinks<br>(One drink is equal to 350ml beer, 150ml of wine, or a shot of spirits.)
In the past month, did you ever drink enough alcohol to become drunk?	◯ Yes<br>◯ No<br>◯ Dont know
How many times did this happen in the last month?	◯ 1<br>◯ 2-3<br>◯ 4-5<br>◯ 5-6<br>◯ 7-10<br>◯ 11-15<br>◯ 15 or more

Mark as "Complete" and select "Save and go to next instrument" when complete

Sedentary Behaviour and Sleep

SEDENTARY BEHAVIOUR AND SLEEP

The following question is about sitting or reclining at work, at home, or with friends including time spent sitting at a desk, sitting with friends, travelling in car, bus, reading, playing cards or watching television, but do not include time spent sleeping.

How much time do you usually spend sitting or reclining on a typical day?

- ◯ Less than an hour
- ◯ 1-2 hours
- ◯ 2-4 hours
- ◯ 4-6 hours
- ◯ 6-10 hours
- ◯ More than 10 hours
- ◯ Dont know

How many days a week do you exercise (strenuous physical activity or sport) for at least 10 min?

- ◯ Never
- ◯ 1-3 days a week
- ◯ 4-5 days a week
- ◯ 6-7 days a week

On days you exercise, how long do you usually spend exercising (strenuous physical activity or sport)?

- ◯ None
- ◯ 0-14 minutes
- ◯ 15-29 minutes
- ◯ 30-59 minutes
- ◯ 60-90 minutes
- ◯ More than 90 minutes
- ◯ Don't know

SLEEP

The following questions relate to your usual sleep habits during the past month only. Your answers should indicate the most accurate reply for the majority of days and nights in the past month.

During the past month, what time have you usually gone to bed at night?	◯ 12am (midnight)<br>◯ 1am<br>◯ 2am<br>◯ 3am<br>◯ 4am<br>◯ 5am<br>◯ 6am<br>◯ 7am<br>◯ 8am<br>◯ 9am<br>◯ 10am<br>◯ 11am<br>◯ 12pm<br>◯ 1pm<br>◯ 2pm<br>◯ 3pm<br>◯ 4pm<br>◯ 5pm<br>◯ 6pm<br>◯ 7pm<br>◯ 8pm<br>◯ 9pm<br>◯ 10pm<br>◯ 11pm
During the past month, how long (in minutes) has it usually taken you to fall asleep each night?	◯ 0-10min<br>◯ 11-20min<br>◯ 21-30min<br>◯ 31-45min<br>◯ 46-59 min<br>◯ 1-1.5 hours<br>◯ Over 1.5 hours<br>◯ Don't know
During the past month, what time have you usually gotten up in the morning?	◯ 12am<br>◯ 1am<br>◯ 2am<br>◯ 3am<br>◯ 4am<br>◯ 5am<br>◯ 6am<br>◯ 7am<br>◯ 8am<br>◯ 9am<br>◯ 10am<br>◯ 11am<br>◯ 12pm<br>◯ 1pm<br>◯ 2pm<br>◯ 3pm<br>◯ 4pm<br>◯ 5pm<br>◯ 6pm<br>◯ 7pm<br>◯ 8pm<br>◯ 9pm<br>◯ 10pm<br>◯ 11pm

During the past month, how many hours of actual sleep did you get at night?(This may be different than the number of hours you spent in bed.)

◯ less than 4 hours
◯ 4-5 hours
◯ 5-6 hours
◯ 6-7 hours
◯ 7-8 hours
◯ 8-9 hours
◯ over 9 hours

During the past month, how often have you had trouble sleeping because you . . .

SLEEP

	Not during the past month	Less than once a week	Once or twice a week	Three or more times a week	Dont know
Cannot get to sleep within 30 minutes	◯	◯	◯	◯	◯
Wake up in the middle of the night or early morning	◯	◯	◯	◯	◯
Cannot breathe comfortably	◯	◯	◯	◯	◯
Cough or snore loudly	◯	◯	◯	◯	◯
Have pain	◯	◯	◯	◯	◯

Mark as "Complete" and select "Save and go to next instrument" when complete

Anthropometrics and Biomarkers

____

BLOOD PRESSURE & PULSE

Now we are going to take the measurements that we were talking about at the beginning. We will give you these measurements if you would like them. We will take your blood pressure 3 times and need to wait 2-3 minutes between measurements.

Reading 1: Systolic (SYS) (Hg) ______________________

Reading 1: Diastolic (DIA) (Hg) ______________________

Reading 1: Pulse (bpm) ______________________

Reading 2: Systolic (SYS) (Hg) ______________________

Reading 2: Diastolic (DIA) (Hg) ______________________

Reading 2: Pulse (bpm) ______________________

Reading 3: Systolic (SYS) (Hg) ______________________

Reading 3: Diastolic (DIA) (Hg) ______________________

Reading 3: Pulse (bpm) ______________________

HEIGHT, WEIGHT, AND WAIST MEASUREMENTS

Height (cm) ______________________
(Must include one decimal place (i.e. 145.0))

Are you pregnant? (Only ask of women)
◯ Yes
◯ No
◯ Don't know

Weight (kgs) ______________________
(Weight must include one decimal (e.g. 97.8). Enter 111.1 if too heavy for scale; make a note in the interview notes as well))

Body Mass Index: __________________________________

Waist circumference (cm): __________________________________

DRY BLOOD SPOTS

(FOR INTERVIEWER) Collect the Dry Blood Spots now.

(FOR INTERVIEWER) Have the dried blood spots been collected?
◯ Yes
◯ No

Does the respondent want the results of the blood glucose test reported back to them?
◯ Yes
◯ No
(Note: respondent must give a phone number and/or email address to have results reported back)

Scan DBS Barcode __________________________________

Manually enter barcode if barcode scanner is not working: __________________________________

Confirm manually entered barcode: __________________________________

Mark as "Complete" and select "Save and go to next instrument" when complete

Sexual Partnership

SEXUAL PARTNERSHIP

The following section asks questions about your sexual practices over the last 12 months. Because a broad range of men and women are participating in this study, some of the questions may not apply to you. However, we ask the same questions to all participants. Your answers are completely confidential.

During the last 12 months, how many sexual partners have you had? __________________

Of the [q_21_1] of partners you've had in the last 12 months, how many lived in or near [q_1_5]? __________________

Of the [q_21_1] of partners you've had in the last 12 months, how many live in [current_residence] or outside [q_1_5]? __________________

Note to interviewer: Total is [q_21_1_3]. Total should be [q_21_1] . __________________

How many TOTAL sexual partners have you had in your lifetime? __________________

When was the last time you had sexual intercourse? MONTH

- ◯ January
- ◯ February
- ◯ March
- ◯ April
- ◯ May
- ◯ June
- ◯ July
- ◯ August
- ◯ September
- ◯ October
- ◯ November
- ◯ December
- ◯ Don't know

When was the last time you had sexual intercourse? YEAR

- ◯ 2019
- ◯ 2018
- ◯ 2017
- ◯ 2016
- ◯ 2015
- ◯ 2014
- ◯ 2013
- ◯ 2012
- ◯ 2011
- ◯ 2010
- ◯ 2009
- ◯ 2008
- ◯ 2007
- ◯ 2006
- ◯ 2005
- ◯ 2004
- ◯ 2003
- ◯ 2002
- ◯ 2001
- ◯ 2000
- ◯ 1999
- ◯ 1998
- ◯ 1997
- ◯ 1996
- ◯ 1995
- ◯ 1994
- ◯ 1993
- ◯ 1992
- ◯ 1991
- ◯ 1990
- ◯ 1989
- ◯ 1988
- ◯ 1987
- ◯ 1986
- ◯ 1985
- ◯ 1984
- ◯ 1983
- ◯ 1982
- ◯ 1981
- ◯ 1980

For the next question(s), follow these definitions:
Regular partner: is someone who is special to you, like a husband / wife / boyfriend / girlfriend, or lover.
Casual partner: is a person you know but are not in a relationship with, whether you had sex only once or many times.
Anonymous partner: is someone whose name you did not know the day before you had sex. A sex partner who is a prostitute could be either casual or anonymous

---

Was your most recent sexual partner:

- ◯ Regular
- ◯ Casual
- ◯ Anonymous
- ◯ Don't know

Where was your most recent sexual intercourse with this partner?	◯ Same village/neighbourhood in [q_1_5]<br>◯ Village/neighbourhood nearby [q_1_5]<br>◯ Same village/neighbourhood in [current_residence]<br>◯ Village/neighbourhood nearby [current_residence]<br>◯ Other<br>◯ Don't know<br>(If the answer categories repeat (i.e. origin location and current residence are the same), select the answer from the first two options (unless other or don't know))
Please specify	__________________________
Did you use a condom the last time you had sexual intercourse?	◯ Yes<br>◯ No<br>◯ Dont know
Why did you not use a condom?	◯ Didn't have one<br>◯ Convenience<br>◯ Decreased pleasure<br>◯ Didn't want to ruin the "mood"<br>◯ Trusted partner<br>◯ Trying to get pregnant<br>◯ Other<br>◯ Dont know
Please specify	__________________________
The first time you had sex with this partner, did you know his or her HIV status?	◯ Yes<br>◯ No<br>◯ Dont know
What is your relationship with this partner?	◯ Boyfriend<br>◯ Girlfriend<br>◯ Living together without lobola<br>◯ Spouse (civil married) without lobola<br>◯ Spouse with lobola done<br>◯ Don't know
When did you get married or start living together? MONTH	◯ January<br>◯ February<br>◯ March<br>◯ April<br>◯ May<br>◯ June<br>◯ July<br>◯ August<br>◯ September<br>◯ October<br>◯ November<br>◯ December<br>◯ Don't know

When did you get married or start living together?<br>YEAR	◯ 2019<br>◯ 2018<br>◯ 2017<br>◯ 2016<br>◯ 2015<br>◯ 2014<br>◯ 2013<br>◯ 2012<br>◯ 2011<br>◯ 2010<br>◯ 2009<br>◯ 2008<br>◯ 2007<br>◯ 2006<br>◯ 2005<br>◯ 2004<br>◯ 2003<br>◯ 2002<br>◯ 2001<br>◯ 2000<br>◯ 1999<br>◯ 1998<br>◯ 1997<br>◯ 1996<br>◯ 1995<br>◯ 1994<br>◯ 1993<br>◯ 1992<br>◯ 1991<br>◯ 1990<br>◯ 1989<br>◯ 1988<br>◯ 1987<br>◯ 1986<br>◯ 1985<br>◯ 1984<br>◯ 1983<br>◯ 1982<br>◯ 1981<br>◯ 1980
Is this relationship ongoing?	◯ Yes<br>◯ No<br>◯ Dont know
In the last 12 months have you had symptoms of, been diagnosed with, or have you been treated for a sexually transmitted disease? [such as trichomonas, gonorrhea or Chlamydia, drop, or any bad smelling/abnormal vaginal or penile discharge, sore or ulcer]	◯ Yes<br>◯ No<br>◯ Dont know

Mark as "Complete" and select "Save and go to next instrument" when complete

HIV

HIV

Have you ever had an HIV test?	◯ Yes<br>◯ No<br>◯ Dont know
When were you tested the last time? MONTH	◯ January<br>◯ February<br>◯ March<br>◯ April<br>◯ May<br>◯ June<br>◯ July<br>◯ August<br>◯ September<br>◯ October<br>◯ November<br>◯ December<br>◯ Don't know<br>(If respondent does not know exact date, use best estimate)

When were you tested the last time? YEAR	◯ 2019<br>◯ 2018<br>◯ 2017<br>◯ 2016<br>◯ 2015<br>◯ 2014<br>◯ 2013<br>◯ 2012<br>◯ 2011<br>◯ 2010<br>◯ 2009<br>◯ 2008<br>◯ 2007<br>◯ 2006<br>◯ 2005<br>◯ 2004<br>◯ 2003<br>◯ 2002<br>◯ 2001<br>◯ 2000<br>◯ 1999<br>◯ 1998<br>◯ 1997<br>◯ 1996<br>◯ 1995<br>◯ 1994<br>◯ 1993<br>◯ 1992<br>◯ 1991<br>◯ 1990<br>◯ 1989<br>◯ 1988<br>◯ 1987<br>◯ 1986<br>◯ 1985<br>◯ 1984<br>◯ 1983<br>◯ 1982<br>◯ 1981<br>◯ 1980<br>(If respondent does not know exact date, use best estimate)
Where was your most recent test done?	◯ Same village/neighbourhood in [q_1_5]<br>◯ Village/neighbourhood nearby [q_1_5]<br>◯ Same village/neighbourhood in [current_residence]<br>◯ Village/neighbourhood nearby [current_residence]<br>◯ Other<br>◯ Don't know<br>(If the answer categories repeat (i.e. origin location and current residence are the same), select the answer from the first two options (unless other or don't know))
Please specify	__________________________________
Did you receive your results?	◯ Yes<br>◯ No<br>◯ Dont know

If you wouldn't mind sharing, what was the result of your most recent HIV test?	◯ Positive<br>◯ Negative<br>◯ Don't want to say/Refuse<br>◯ Don't know
Have you ever informed anyone about your HIV status?	◯ Yes<br>◯ No<br>◯ Dont know
Who did you inform about your HIV status?	☐ Spouse/current partner<br>☐ Girlfriend/boyfriend<br>☐ Parent<br>☐ Brother/sister<br>☐ Relative<br>☐ Friend<br>☐ Religious leader<br>☐ Other<br>☐ Don't know
Please specify	________________________________
Have you ever been on antiretrovirals/ARTs?	◯ Yes<br>◯ No<br>◯ Dont know
Are you currently on antiretrovirals/ARTs?	◯ Yes<br>◯ No<br>◯ Dont know

Mark as "Complete" and select "Save and go to next instrument" when complete

Conclusion

CONCLUSION

END: Many thanks for your time and contribution to the study. We will be contacting you again a year from now to follow up. Can you please provide the best way to get in touch with you a year from now.

Telephone # ______________________

Email address ______________________

Telephone # of a friend or relative #1 ______________________

Email address of a friend or relative #1 ______________________

Telephone # of a friend or relative #2 ______________________

Email address of a friend or relative #2 ______________________

End time of interview ______________________

Interview Length ______________________
